# Cancer Therapy-Related Cardiac Dysfunction: Pooled Incidence of Subclinical and Clinical Presentations Using Multimodal Multi-Parametric Imaging—A Systematic Review and Meta Analysis

**DOI:** 10.3390/jcm15124520

**Published:** 2026-06-11

**Authors:** Mohamad Altamimi, Elfatih A. Hasabo, Ammar Elgadi, Abdullatif Yasir H. Eissa, Salma S. Alrawa, Amira A. Aboali, Ibrahim M. Mahgoub, Malaz M. Abdalmotalib, Tibyan Noorallah Mohammed, Sanaa Ali, Esraa S. A. Alfadul, Muhammed Ali Jawed, Osama Soliman

**Affiliations:** 1 Royal College of Surgeons in Ireland (RCSI), University of Medicine and Health Sciences, D02 YN77 Dublin, Ireland; mdt2019@gmail.com (M.A.); elfatih.ahmed.hasabo@gmail.com (E.A.H.); sana100571@gmail.com (S.A.); dr_ali147@yahoo.co.uk (M.A.J.); 2 Cardiovascular Research Institute Dublin (CVRI), Mater Private Network, Eccles Street, D07 KWR1 Dublin, Ireland; 3 Faculty of Medicine, University of Khartoum, Khartoum 11115, Sudan; ammartarig56@gmail.com (A.E.); eissaabdullatif55@gmail.com (A.Y.H.E.); salmalrwa@gmail.com (S.S.A.); ibrahimberkiah@gmail.com (I.M.M.); malaz.abdalmotalib@gmail.com (M.M.A.); tibyan.noorallah@gmail.com (T.N.M.); esraaalfadul.uofk@gmail.com (E.S.A.A.); 4 Damanhour Teaching Hospital, General Organization for Teaching Hospitals and Institutes, Damanhour 22511, Egypt; amiraaboali4@gmail.com; 5 Insight Research Institute, Insight Hospital and Medical Center, Chicago, IL 60616, USA

**Keywords:** CTRCD, cardiotoxicity, global longitudinal strain, left ventricular ejection fraction, echocardiography, cardiac magnetic resonance

## Abstract

**Objective:** To estimate the pooled incidence of cancer therapy-related cardiac dysfunction (CTRCD), assess longitudinal changes in global longitudinal strain (GLS) and left ventricular ejection fraction (LVEF), and summarise the available evidence comparing echocardiography and cardiac magnetic resonance (CMR) for serial functional assessment. **Methods:** We performed a systematic review and meta-analysis of observational studies reporting CTRCD in adults receiving cancer therapy. Pooled CTRCD incidence was estimated, study-level meta-regression explored associations between baseline mean GLS and LVEF and CTRCD event rates, and longitudinal changes in GLS and LVEF were compared between CTRCD and non-CTRCD cohorts across follow-up visits. Exploratory subgroup analyses compared findings derived from echocardiography and CMR. **Results:** Thirty-three studies were included (total enrolled *n* = 2083; analysed at follow-up *n* = 1973), including 27 echocardiography-only studies, 4 CMR-only studies, and 2 studies reporting both modalities at baseline. The pooled incidence of CTRCD was 27% (95% CI 0.20–0.35), with substantial heterogeneity across studies. In study-level meta-regression, baseline mean GLS (*p* = 0.195) and baseline mean LVEF (*p* = 0.668) were not significantly associated with CTRCD event rates. Compared with non-CTRCD cohorts, CTRCD cohorts showed greater worsening in GLS across all analysed follow-up intervals. Within CTRCD cohorts, both GLS and LVEF deteriorated over time, whereas non-CTRCD cohorts showed smaller changes overall. Exploratory subgroup analyses did not detect statistically significant differences between echocardiography and CMR in the available datasets. **Conclusions:** CTRCD is reported in approximately one-quarter of patients across published studies, although estimates vary substantially by population, therapy, follow-up schedule, and CTRCD definition. Longitudinal deterioration in GLS appears earlier and more consistently than decline in LVEF, supporting the role of serial deformation imaging in surveillance. Baseline study-level mean GLS and LVEF were not significantly associated with CTRCD event rates, and direct comparative evidence between echocardiography and CMR remains limited.

## 1. Introduction 

The landscape of oncological care has undergone a remarkable transformation over the past decade, with substantial improvements in cancer survival rates attributable to advances in early diagnostic imaging and the development of novel chemotherapeutic agents. This therapeutic success has been tempered by an increasingly recognised burden of treatment related cardiovascular complications, which represent a significant source of morbidity and mortality among cancer survivors [1,2]. Cancer therapy-related cardiac dysfunction (CTRCD) remains a critical challenge in contemporary cardio-oncology and often necessitates treatment interruption or discontinuation, with implications for cancer recurrence and overall survival [2]. CTRCD definitions vary across studies and contemporary guidelines, but commonly include a decline in LVEF beyond predefined thresholds, often supported by changes in GLS, symptoms, or cardiac biomarkers [3]. The incidence of CTRCD varies by chemotherapeutic regimen, with estimates suggesting that up to 40% of patients may develop cardiac dysfunction within five years following chemotherapy completion [2]. Anthracyclines and anti-human epidermal growth factor receptor 2 (anti-HER2)-targeted agents such as trastuzumab are associated with elevated CTRCD risk [4,5]. Contemporary practice uses CTRCD to denote impaired cardiac contractile function, typically diagnosed through echocardiographic assessment or elevation of cardiac biomarkers such as troponin and B-type natriuretic peptide (BNP) [3,6].

Left ventricular ejection fraction (LVEF) has traditionally served as the principal metric for cardiac function assessment and monitoring in patients receiving cardiotoxic chemotherapy [7,8]. LVEF is routinely evaluated through echocardiography and remains central to many surveillance pathways [7]. However, LVEF has limitations for early detection of CTRCD. Measurement accuracy and sensitivity at early stages are suboptimal, with substantial inter- and intra-observer variability [4,5]. By the time LVEF reductions become apparent, myocardial damage has frequently progressed and may be irreversible, which limits the therapeutic window for cardioprotective interventions [4,5].

Advances in cardiac imaging have introduced more sensitive techniques for detecting subclinical myocardial dysfunction. Left ventricular deformation can be evaluated through speckle tracking echocardiography, which assesses myocardial strain and strain rate [9]. Global longitudinal strain (GLS) is a promising parameter with superior reproducibility compared to LVEF, and enhanced sensitivity for detecting early myocardial injury [10]. In patients with preserved LVEF greater than 50 percent, a relative reduction in GLS exceeding 15 percent from baseline has been proposed as a criterion for subclinical myocardial injury [3,11,12,13]. GLS abnormalities may manifest prior to LVEF decline, enabling earlier identification of CTRCD and supporting timely cardioprotective strategies [11,14].

Substantial heterogeneity exists in GLS measurement techniques, CTRCD definitions, patient populations, treatment regimens, and follow-up schedules. In addition, the available evidence directly comparing echocardiographic and CMR-based strain assessment in cardio-oncology remains limited. Accordingly, this systematic review and meta-analysis aimed to estimate the pooled incidence of CTRCD, evaluate longitudinal changes in GLS and LVEF in patients with and without CTRCD, and summarise the available evidence across echocardiography- and CMR-based imaging studies.

## 2. Methods

This systematic review was conducted and reported in accordance with the Preferred Reporting Items for Systematic Reviews and Meta-Analyses (PRISMA) 2020 reporting guideline [15] and the Cochrane Handbook for Systematic Reviews of Interventions [16], and was prospectively registered in PROSPERO, University of York (registration number CRD420251043707). The primary aim of this review was to estimate the proportion of patients who developed CTRCD during longitudinal follow-up after exposure to cancer therapy.

### 2.1. Search Strategy

A comprehensive literature search was conducted on the 29 October 2025, using the following electronic databases: PubMed, Scopus, Web of Science (WoS), Cochrane, and Embase. The search combined terms related to myocardial strain imaging, cardiotoxicity, cancer therapies, and cardiac imaging modalities, using the following keywords: [(“global longitudinal strain” OR GLS OR “circumferential strain” OR “strain rate” OR strain) AND (cardiotoxicity OR cardiomyopathy OR “cancer therapy” OR chemotherapy OR chemotherap* OR trastuzumab OR anthracycline OR doxorubicin OR adriamycin OR idarubicin OR epirubicin OR daunorubicin OR mitoxantrone OR 5-fluorouracil OR paclitaxel OR cyclophosphamide) AND (“echocardiography” OR “cardiac magnetic resonance” OR “CMR”)]. The conceptual search strategy was adapted to the syntax and indexing structure of each database. Full details of the database-specific search strategies are provided in (Supplementary Table S1).

### 2.2. Eligibility Criteria and Screening

We included observational studies enrolling adults (≥18 years) receiving potentially cardiotoxic cancer therapy in whom CTRCD was reported using echocardiography, CMR, or both. Eligible study designs comprised prospective and retrospective observational studies. We excluded case reports, systematic reviews, meta-analyses, narrative reviews, consensus statements, commentaries, and studies that did not provide a clear definition of CTRCD.

Study selection was performed in two stages: title and abstract screening, followed by full-text review. Title/abstract screening and full-text review were performed independently by two reviewers for each record. Any discrepancies arising during the screening process were resolved through discussion and consensus; if disagreement persisted, a final decision was made by MA.

### 2.3. Data Extraction

Data extraction was performed using a predefined extraction form by multiple reviewers, with verification and consensus review of extracted variables before analysis. Extracted baseline and study-level characteristics included study design, type of cancer, chemotherapy agent, definition of CTRCD, follow-up duration, age, proportion of female participants, baseline LVEF, baseline GLS, imaging modality, and analysis software used. Outcome data extracted from each study comprised the incidence of CTRCD following chemotherapy, all-cause mortality among patients with and without CTRCD, and changes in GLS in patients who developed CTRCD. Both clinical and subclinical CTRCD, as reported by the original studies, were captured during data extraction. CTRCD was extracted according to the definition reported in each included study. Because CTRCD definitions varied substantially, each study was classified according to the operational definition used, into: LVEF-only, GLS-only, combined imaging, imaging plus biomarkers, guideline-based composite, or definitions not reported. These categories were summarised and were used to contextualise heterogeneity in pooled CTRCD incidence.

### 2.4. Risk of Bias Assessment

Risk of bias was assessed independently by two reviewers using the ROBINS-I tool [17]. Overall, most included studies were judged to be at low risk of bias across the assessed domains. However, a small number of studies showed moderate or high risk of bias in selected domains, particularly confounding and missing data, and a few studies were also considered to have a higher overall risk of bias (Supplementary Figure S1).

### 2.5. Statistical Analysis

Data from the included studies were analysed using meta-analytic methods [18]. All statistical analyses were conducted using R software version 4.5.1 with the “meta” and “dmetar” packages. Continuous outcomes, including (GLS) and (LVEF), were pooled using the metacont function and were reported as mean differences (MD) with 95% confidence intervals (CI) and prediction intervals (PI) [18]. Hedges’ method was applied to correct for potential small-sample bias. Proportions (CTRCD) were synthesized using the “metaprop” function and were reported as pooled proportions with 95% CI and PI [18]. For proportions, we used a Logit transformation (PLOGIT) via the inverse variance method, and studies with zero events were handled using a standard continuity correction.

Results for all outcomes were displayed using forest plots. To account for the anticipated heterogeneity between studies, the random-effects model was used for most comparisons. The restricted maximum likelihood (REML) estimator was used for tau2, and the Hartung-Knapp (HK) adjustment was applied to yield more conservative 95% confidence intervals. The fixed-effect model was specifically used for ECHO/CMR comparisons due to the low overall heterogeneity. For GLS, less negative values indicate worse myocardial function; therefore, a positive change from baseline reflects deterioration in longitudinal systolic function. Heterogeneity was assessed using I^2^ and Cochran’s Q. Pre-specified follow-up windows were used to harmonise reported assessment timepoints across studies into Visit 1, Visit 2, Visit 3, and Last Visit categories, with study-level follow-up type and duration provided in Supplementary Table S2. Subgroup meta-analyses (Supplementary Figures S2 and S3) were performed according to CTRCD (clinical and subclinical) and follow-up duration. Then, a leave-one-out sensitivity analysis (Supplementary Figures S4 and S8) was conducted using the “metainf” function to evaluate the sources of heterogeneity and the stability of the pooled estimates. Studies with missing standard deviations or primary mean values for specific time points were excluded from those specific analyses using listwise deletion. No imputation was performed. Also, as the primary analysis focused on continuous mean differences (MD) rather than binary outcomes, logit transformations and zero-event handling were not required for the primary GLS and EF comparisons.

## 3. Results

### 3.1. Search in Databases

**Figure 1:**
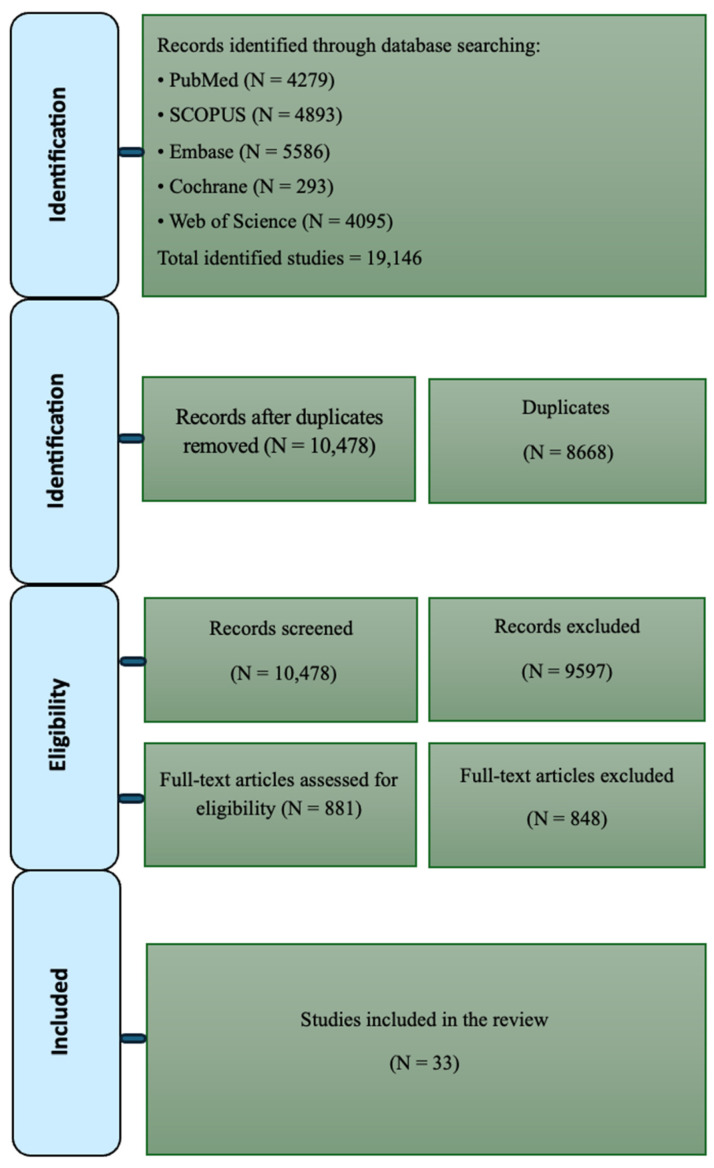
PRISMA Flow Diagram.

The database search identified 19,148 records across PubMed, Scopus, Web of Science, Embase, and Cochrane. After removing 8668 duplicates, 10,478 records underwent title and abstract screening. Of these, 881 full-text articles were assessed, and 33 studies [19,20,21,22,23,24,25,26,27,28,29,30,31,32,33,34,35,36,37,38,39,40,41,42,43,44,45,46,47,48,49] met the inclusion criteria. A summary of these steps was reported in the PRISMA flow diagram of the study (Figure 1).

### 3.2. Baseline Characteristics of Included Studies

Across 33 included studies (echocardiography-only *n* = 27; CMR-only *n* = 4; both modalities at baseline *n* = 2), the total sample size was 2083; age ranged from 42 (20–69) to 61 (51–69.5) years and the female proportion ranged from 16% to 100%. Baseline GLS (echo) ranged from −22.77 ± 2.45 to −17.6 ± 1.3 and baseline LVEF (echo) ranged from 58.0 ± 3.0 to 69.76 ± 6.13. Baseline GLS (CMR) ranged from −20.0 ± 2.0 to −14.9 ± 2.1 and baseline LVEF (CMR) ranged from 61 ± 5 to 65.2 ± 6.8; studies reporting both modalities at baseline reported GLS (echo) of −21.8 ± 1.61 and −18.6 ± 2.6 with LVEF (echo) of 65.2 ± 6.19 and 58.5 ± 6.0, and GLS (CMR) of −19.16 ± 2.71 and −18.8 ± 1.5 with LVEF (CMR) of 72.5 ± 6.36 and 58.0 ± 5.6. Breast cancer was the most frequently reported cancer type across the included studies. Anthracyclines were the most frequently reported cancer therapy (Table 1).

### 3.3. Imaging-Based CTRCD Outcomes

Across the three imaging strata, the total analysed population was 1973. In echocardiography-only studies (*n* = 27), the analysed population (N) ranged from 26 to 169, follow-up ranged from 3 to 15 months, subclinical CTRCD ranged from 8 (16%) to 52 (78%) patients, clinical CTRCD ranged from 0 (0%) to 18 (29.5%) patients, and total CTRCD ranged from 5 (10%) to 52 (78%) patients. In CMR-only studies (*n* = 4), the analysed population (N) ranged from 24 to 66 and follow-up ranged from 3 to 13 months; where reported, subclinical CTRCD ranged from 4 (16.7%) to 24 (41%) patients and clinical CTRCD ranged from 2 (8.3%) to 9 (15%) patients, while total CTRCD across the 4 studies was 6 (25%), 18 (56.3%), 24 (41%), and 9 (14%). In studies reporting both modalities at baseline (*n* = 2), the analysed population (N) ranged from 11 to 32, follow-up was 6 months, and total CTRCD was 9 (28.1%) (Table 2).

### 3.4. Definitions of Cardiotoxicity by Imaging Modality

Cardiotoxicity definitions varied across the included studies and were summarised by imaging subgroup (echocardiography-only, CMR-only, and studies reporting both modalities at baseline). This approach supports consistent reporting and comparison of study-specific criteria (Table 3).

### 3.5. Pooled Incidence of CTRCD

**Figure 2:**
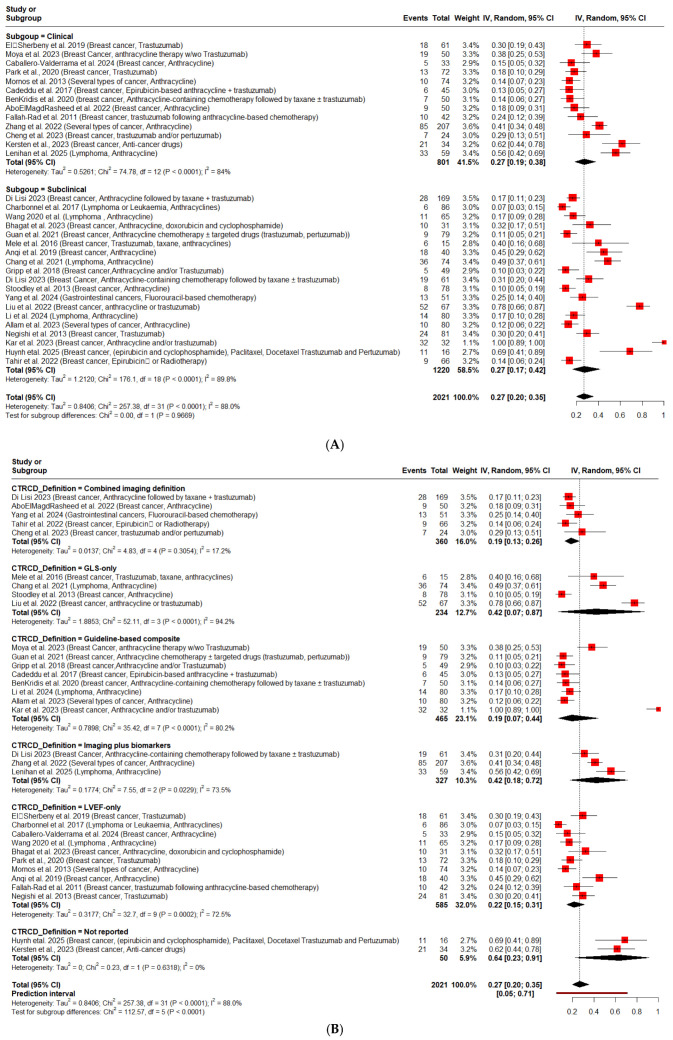
(**A**). Forest plot showing the pooled incidence of cancer therapy-related cardiac dysfunction (CTRCD) stratified by clinical phenotype (clinical vs. subclinical) using a random-effects (inverse-variance) model. (**B**). Forest plot showing the pooled incidence of cancer therapy-related cardiac dysfunction (CTRCD) stratified by definition of CTRCD using a random-effects (inverse-variance) model. [7,14,19,20,21,22,23,24,25,26,27,28,29,30,31,32,33,35,36,37,38,39,40,41,42,43,44,45,46,47,48,49].

A single-group meta-analysis was conducted to determine the overall pooled incidence of CTRCD across the included cohorts. The overall pooled proportion of patients who developed CTRCD was 27% (proportion = 0.27; 95% CI: 0.20 to 0.35). Subgroup analysis by CTRCD classification yielded similar pooled event rates for clinically defined CTRCD and subclinical CTRCD, although heterogeneity remained high in both subgroups. The clinical subgroup included 801 participants (I^2^ = 84%), the subclinical subgroup included 1220 participants (I^2^ = 89.8%), and the overall pooled analysis included 2021 participants. Substantial statistical heterogeneity was observed within the overall pooled analysis (I^2^ = 88.0%, *p* < 0.0001) (Figure 2A). Subgroup analysis by CTRCD definition showed that the guideline-based composite subgroup yielded a pooled proportion of 0.19 (95% CI 0.07 to 0.44). A similar result of 0.19 (95% CI 0.13 to 0.26) was found for the combined imaging subgroup. The pooled proportion was 0.42 (95% CI 0.07 to 0.87) for the GLS-only subgroup; a similar result was reported for the combined imaging plus biomarkers subgroup, with a proportion of 0.42 (95% CI 0.18 to 0.72). Utilizing the LVEF-only definition, the pooled proportion was 0.22 (95% CI 0.15 to 0.31) (Figure 2B).

### 3.6. Meta-Regression Analysis of Baseline Parameters

A study-level meta-regression was performed to explore whether baseline mean GLS and LVEF were associated with CTRCD event rates across studies. Baseline mean GLS was not significantly associated with CTRCD event rate (β = 0.16; 95% CI −0.09 to 0.42; *p* = 0.195), and baseline mean LVEF was likewise not significantly associated with CTRCD event rate (β = 0.03; 95% CI −0.12 to 0.19; *p* = 0.668) Table 4. These analyses are underpowered and should be considered hypothesis-generating only. They should therefore be interpreted as exploratory study-level associations rather than patient-level prognostic effects.

### 3.7. Primary Comparison of Changes in GLS and LVEF from Baseline (CTRCD vs. Non-CTRCD)

**Figure 3:**
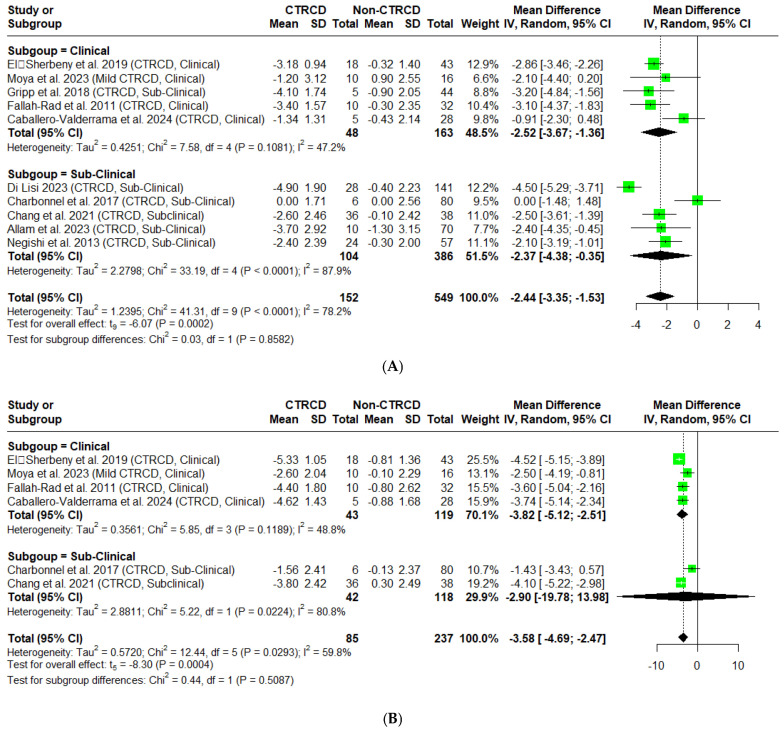

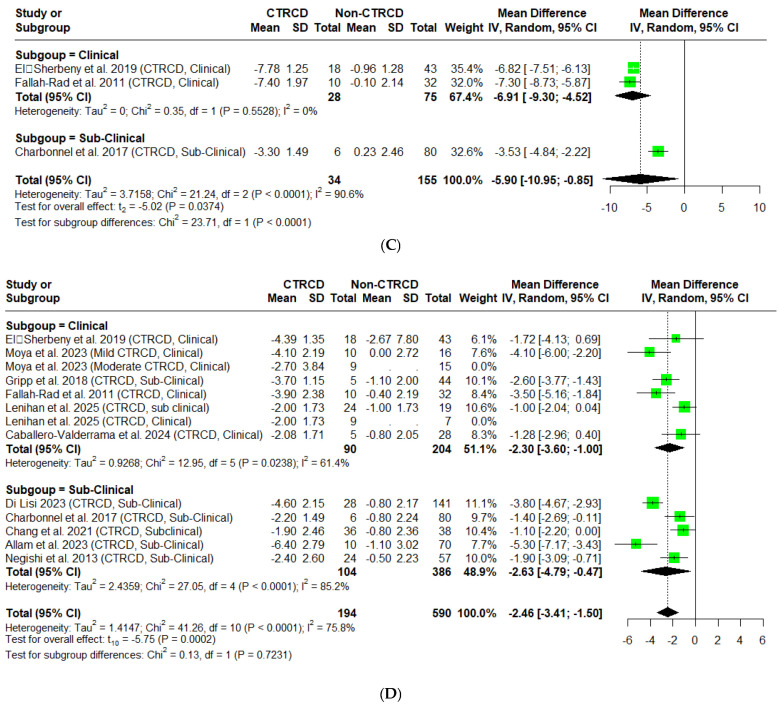
Change in GLS from baseline with and without CTRCD. (**A**) Change in GLS at visit 1 in patients with and without CTRCD. (**B**) Change in GLS at visit 2 in patients with and without CTRCD. (**C**) Change in GLS at visit 3 in patients with and without CTRCD. (**D**) Change in GLS at the last visit in patients with and without CTRCD [7,20,24,26,27,29,31,32,37,42,43].

The meta-analysis evaluated the change from baseline in GLS between patients who developed CTRCD and those who did not. Across all analysed follow-up intervals, the CTRCD cohort consistently demonstrated a statistically significant, more negative change in GLS compared with the non-CTRCD cohort, indicating greater functional impairment. Mean difference (MD) in GLS was −2.44 (95% CI: −3.35 to −1.53; *p* = 0.0002) at visit one, −3.58 (95% CI: −4.69 to −2.47; *p* = 0.0004) at visit two, −5.90 (95% CI: −10.95 to −0.85; *p* = 0.0374) at visit three, and −2.46 (95% CI: −3.41 to −1.50; *p* = 0.0002) at the last visit (Figure 3).

Because GLS is expressed as a negative value, worsening longitudinal function may appear as either a more positive change from baseline within cohorts or a more negative between-group difference, depending on the comparison structure.

**Figure 4:**
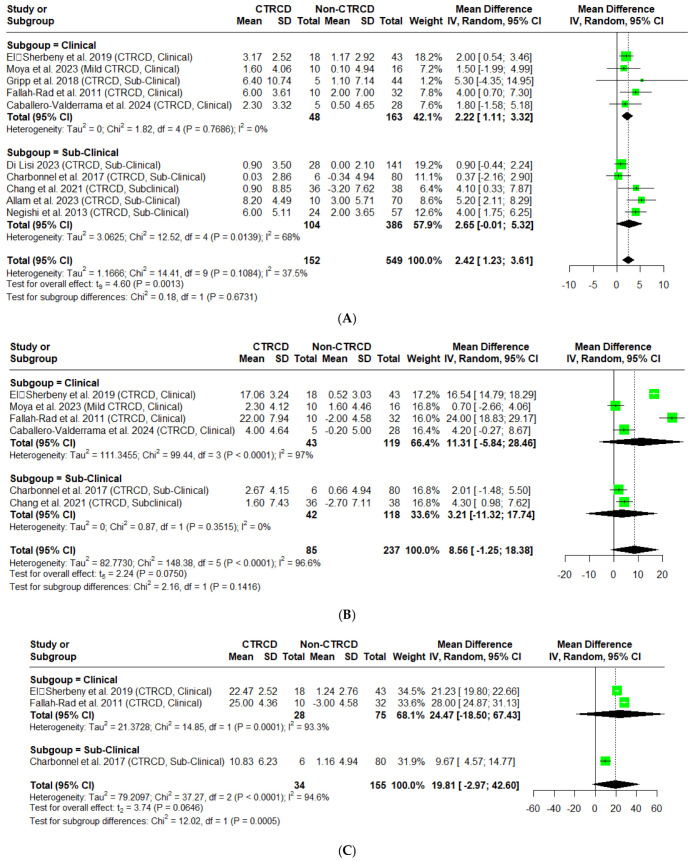

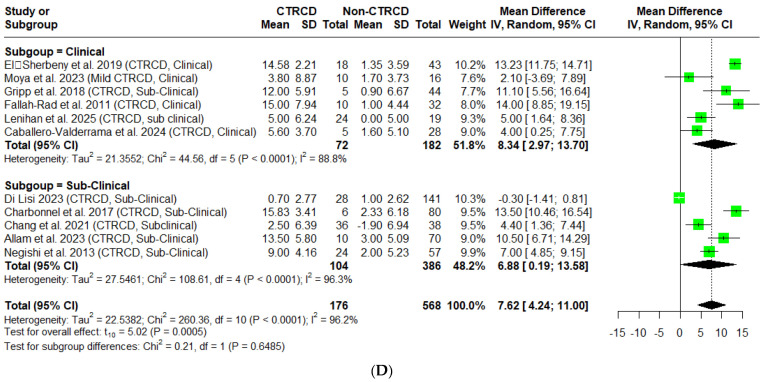
(**A**) Change in LVEF at visit 1 in patients with and without CTRCD. (**B**) Change in LVEF at visit 2 in patients with and without CTRCD. (**C**) Change in LVEF at visit 3 in patients with and without CTRCD. (**D**) Change in LVEF at the last visit in patients with and without CTRCD. [7,20,24,26,27,29,31,32,37,42,43].

The analysis also compared changes in LVEF from baseline between the two cohorts. While the CTRCD group generally experienced a greater change in LVEF, the statistical significance fluctuated across follow-up visits. MD in LVEF was 2.42 (95% CI: 1.23 to 3.61; *p* = 0.0013) at visit one, 8.56 (95% CI: −1.25 to 18.38; *p* = 0.0750) at visit two, 19.81 (95% CI: −2.97 to 42.60; *p* = 0.0646) at visit three, and 7.62 (95% CI: 4.24 to 11.00; *p* = 0.0005) at the last visit (Figure 4).

**Figure 5:**
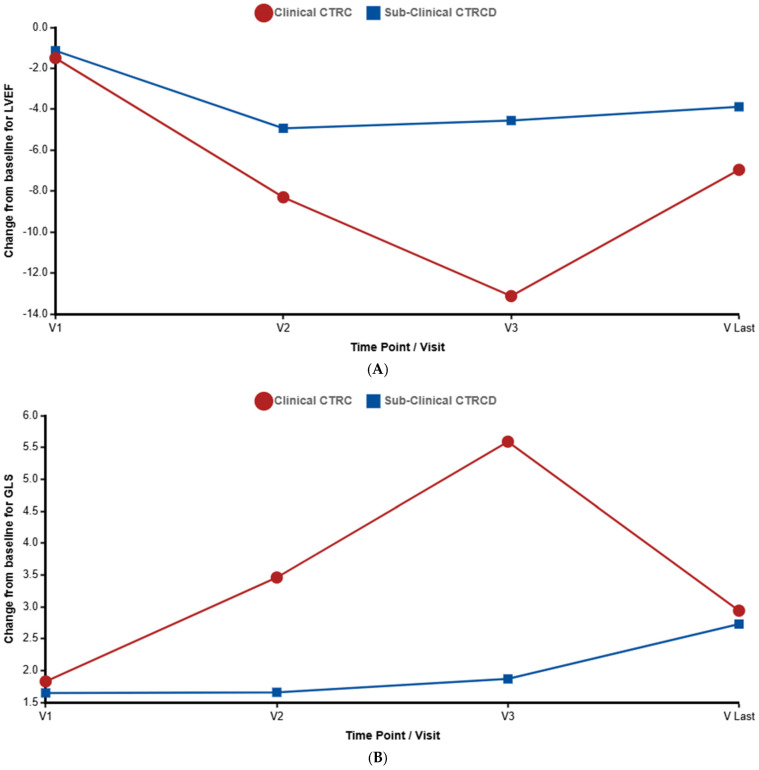
(**A**): Mean change from baseline for LVEF. (**B**): Mean change from baseline for GLS.

Across follow-up visits, the mean change from baseline in both LVEF and GLS indicated greater deterioration in patients with clinical CTRCD than in those with subclinical CTRCD (Figure 5).

### 3.8. Longitudinal Changes Within the CTRCD Cohort

**Figure 6:**
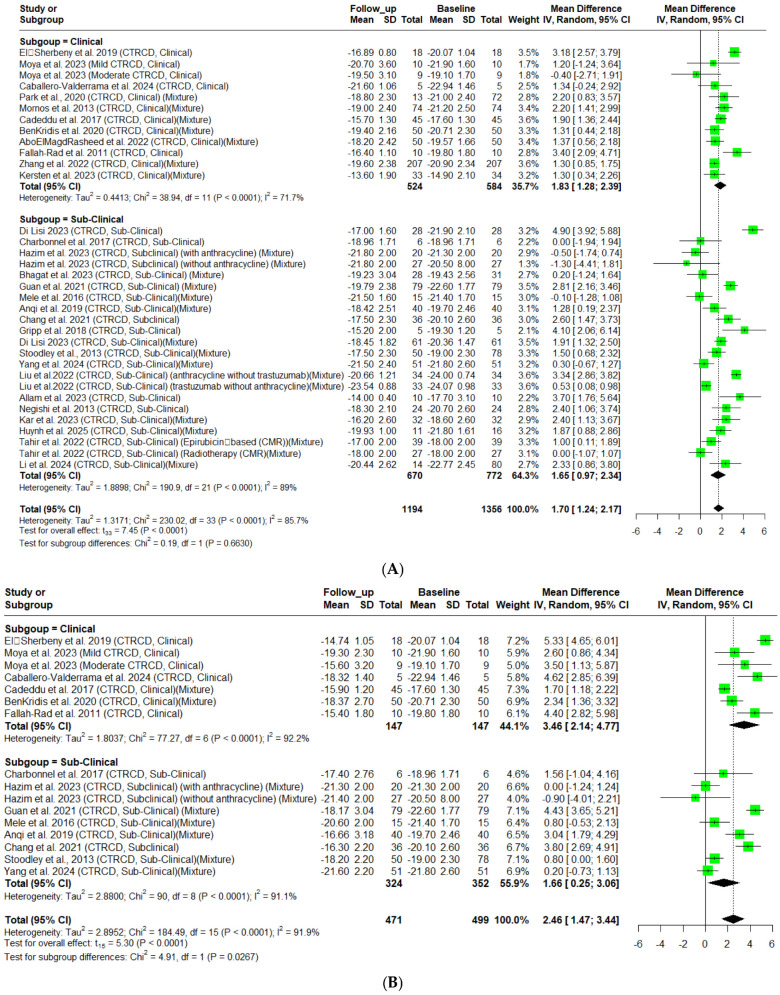

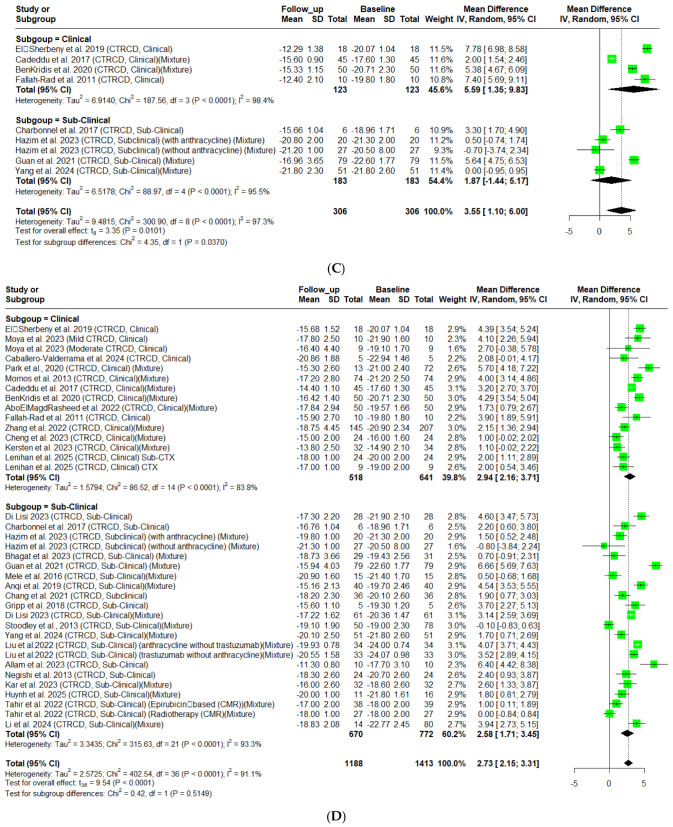
Change in GLS from baseline in CTRCD. (**A**) Change in GLS at visit 1 in CTRCD patients. (**B**) Change in GLS at visit 2 in CTRCD patients. (**C**) Change in GLS at visit 3 in CTRCD patients. (**D**) Change in GLS at last visit in CTRCD patients. [7,14,19,20,21,22,23,24,25,26,27,28,29,30,31,32,33,34,35,36,37,38,39,40,41,42,43,44,45,46,48,49].

A single-group meta-analysis evaluated longitudinal changes specifically within the CTRCD cohort. Patients exhibited a statistically significant positive mean difference in GLS at all follow-up intervals, indicating worsening longitudinal strain. MD in GLS was 1.70 (95% CI: 1.24 to 2.17; *p* < 0.0001) at visit one, 2.46 (95% CI: 1.47 to 3.44; *p* < 0.0001) at visit two, 3.55 (95% CI: 1.10 to 6.00; *p* = 0.0101) at visit three, and 2.73 (95% CI: 2.15 to 3.31; *p* < 0.0001) at the last visit (Figure 6).

### 3.9. Consistent with the GLS Findings, LVEF Significantly Declined Across All Follow-Up Visits in the CTRCD Group

**Figure 7:**
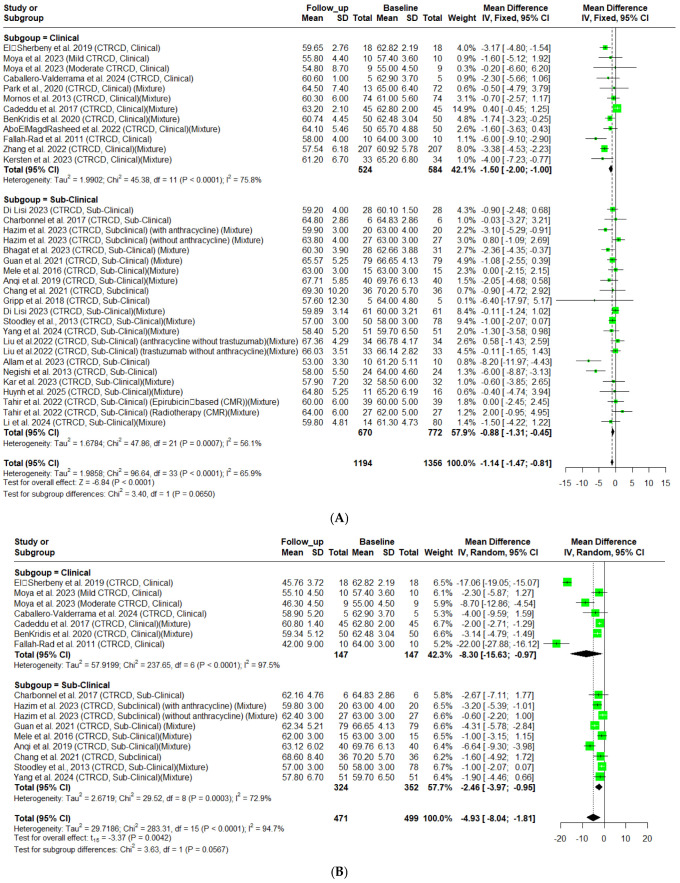

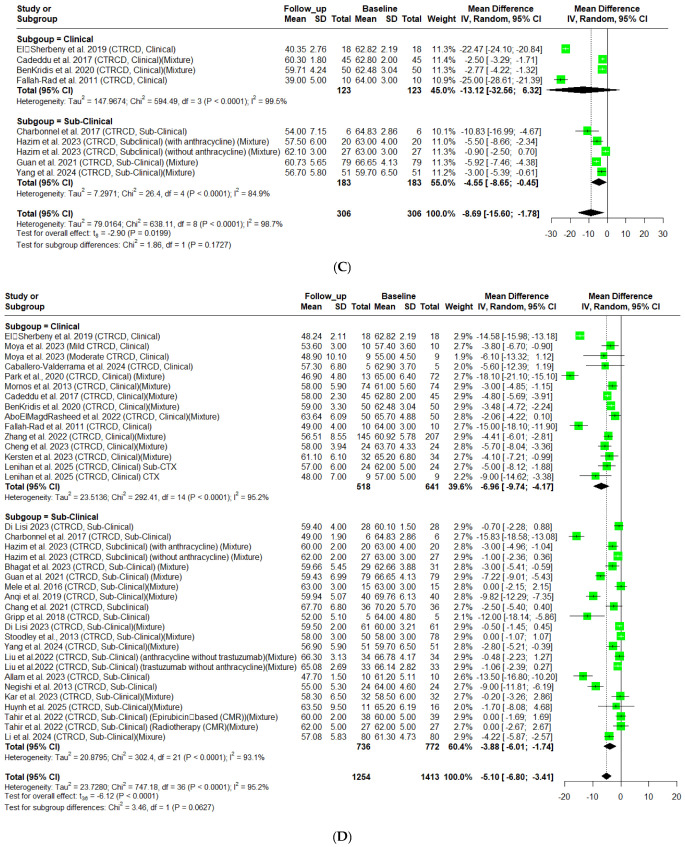
Change in LVEF from baseline in CTRCD. (**A**) Change in LVEF at visit 1 in CTRCD patients. (**B**) Change in LVEF at visit 2 in CTRCD patients. (**C**) Change in LVEF at visit 3 in CTRCD patients. (**D**) Change in LVEF at last visit in CTRCD patients. [7,14,19,20,21,22,23,24,25,26,27,28,29,30,31,32,33,34,35,36,37,38,39,40,41,42,43,44,45,46,48,49].

MD in LVEF was −1.14 (95% CI: −1.47 to −0.81; *p* < 0.0001) at visit one, −4.93 (95% CI: −8.04 to −1.81; *p* = 0.0042) at visit two, −8.69 (95% CI: −15.60 to −1.78; *p* = 0.0199) at visit three, and −5.10 (95% CI: −6.80 to −3.41; *p* < 0.0001) at the last visit (Figure 7).

### 3.10. Longitudinal Changes Within the Non-CTRCD Cohort

**Figure 8:**
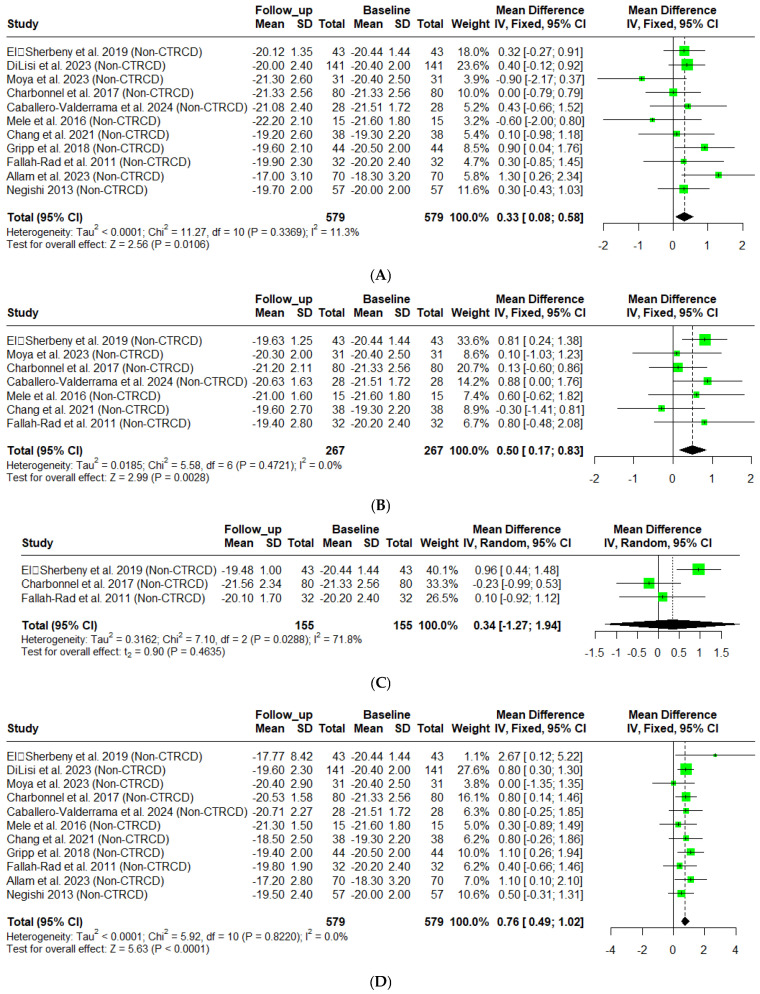
Change in GLS from baseline in non-CTRCD. (**A**) Change in GLS at visit 1 in non-CTRCD patients. (**B**) Change in GLS at visit 2 in non-CTRCD patients. (**C**) Change in GLS at visit 3 in non-CTRCD patients. (**D**) Change in GLS at last visit in non-CTRCD patients. [7,20,24,26,27,29,31,32,40,42,43].

In contrast, non-CTRCD cohorts showed smaller changes in GLS over time, with little evidence of progressive deterioration overall. LVEF remained largely stable, with only small absolute changes across follow-up (visit one: MD = 0.33 [95% CI: 0.08 to 0.58; *p* = 0.0106]; visit two: MD = 0.50 [95% CI: 0.17 to 0.83; *p* = 0.0028]; visit three: MD = 0.34 [95% CI: −1.27 to 1.94; *p* = 0.4635]; last visit: MD = 0.76 [95% CI: 0.49 to 1.02; *p* < 0.0001]) (Figure 8).

**Figure 9:**
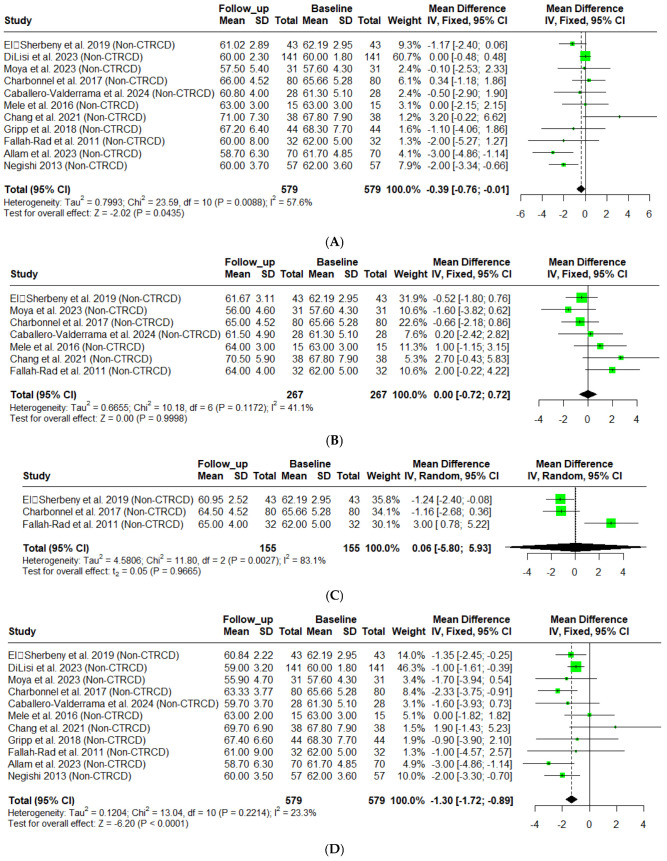
Change in LVEF from baseline in non-CTRCD. (**A**) Change in LVEF at visit 1 in non-CTRCD patients. (**B**) Change in LVEF at visit 2 in non-CTRCD patients. (**C**) Change in LVEF at visit 3 in non-CTRCD patients. (**D**) Change in LVEF at last visit in non-CTRCD patients. [7,20,24,26,27,29,31,32,40,42,43].

Furthermore, LVEF remained largely stable, with only small absolute changes across follow-up (visit one: MD = −0.39 [95% CI: −0.76 to −0.01; *p* = 0.0435]; visit two: MD = 0.00 [95% CI: −0.72 to 0.72; *p* = 0.9998]; visit three: MD = 0.06 [95% CI: −5.80 to 5.93; *p* = 0.9665]; last visit: MD = −1.30 [95% CI: −1.72 to −0.89; *p* < 0.0001]) (Figure 9).

### 3.11. Comparison of Imaging Modalities (Echocardiography vs. CMR)

**Figure 10:**
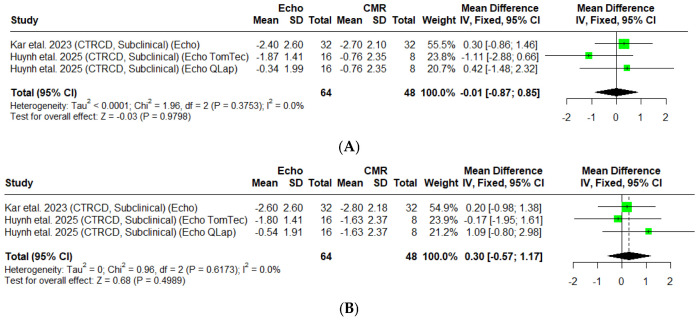
GLS change from baseline in CTRCD: Echo vs. CMR. (**A**) GLS change from baseline at visit 1: Echo vs. CMR. (**B**) GLS change from baseline at last visit: Echo vs. CMR. [35,36].

A subgroup analysis evaluated whether the choice of imaging modality influenced the recorded changes in CTRCD patients. The comparison between echo and CMR for measuring GLS change revealed no statistically significant differences between the two modalities at visit one (MD = −0.01; 95% CI: −0.87 to 0.85; *p* = 0.9798) and at the last visit (MD = 0.30; 95% CI: −0.57 to 1.17; *p* = 0.4989) (Figure 10).

**Figure 11:**
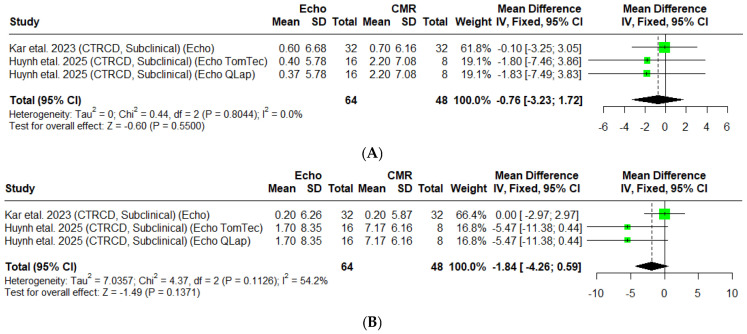
LVEF change from baseline in CTRCD: Echo vs. CMR. (**A**) LVEF change from baseline at visit 1: Echo vs. CMR. (**B**) LVEF change from baseline at last visit: Echo vs. CMR. [35,36].

Similarly, no significant discrepancy between echo and CMR was found in quantifying the change in LVEF from baseline at visit one (MD = −0.76; 95% CI: −3.23 to 1.72; *p* = 0.5500) and at the last visit (MD = −1.84; 95% CI: −4.26 to 0.59; *p* = 0.1371) (Figure 11).

### 3.12. Changes in Cardiac Function Assessed Exclusively by CMR

When evaluated exclusively via CMR, the change in GLS in CTRCD patients showed an overall MD of 1.19 at visit one, which approached but did not reach statistical significance (95% CI: −0.06 to 2.43; *p* = 0.0576), with heterogeneity (I^2^ = 72.1%; χ^2^ = 14.36; df = 4; *p* = 0.0062; τ^2^ = 0.7372).

**Figure 12:**
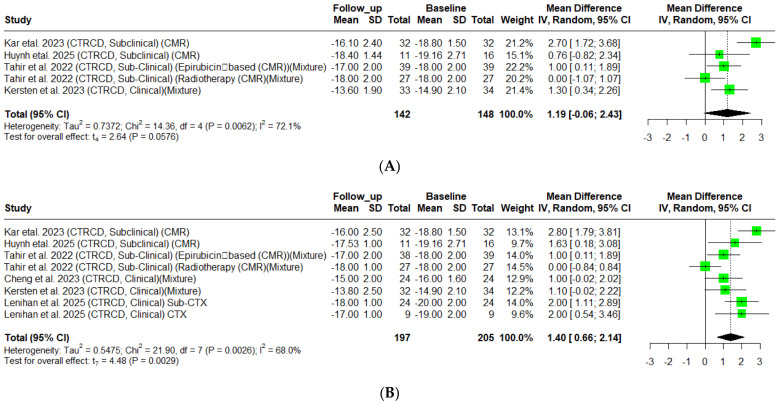
GLS change from baseline in CTRCD: CMR. (**A**) GLS change from baseline at visit 1: CMR. (**B**) GLS change from baseline at last visit: CMR. [14,28,35,36,37,46].

By the final follow-up, a statistically significant deterioration in GLS was confirmed using CMR (MD = 1.40; 95% CI: 0.66 to 2.14; *p* = 0.0029) (Figure 12).

**Figure 13:**
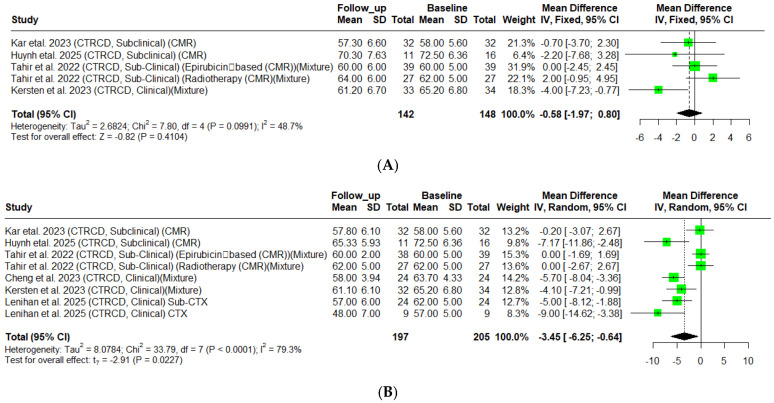
LVEF change from baseline in CTRCD: CMR. (**A**) LVEF change from baseline at visit 1: CMR. (**B**) LVEF change from baseline at last visit: CMR. [14,28,35,36,37,46].

For LVEF measured by CMR, the initial reduction at visit one was not statistically significant (MD = −0.58; 95% CI: −1.97 to 0.80; *p* = 0.4104). However, at the final follow-up, CMR measurements revealed a pronounced and statistically significant decline in LVEF (MD = −3.45; 95% CI: −6.25 to −0.64; *p* = 0.0227) (Figure 13).

### 3.13. Subgroup Analysis Based on a Guideline-Based Definition

In comparative analyses of the studies that follow the guideline-based composite definition of CTRCD, the pooled mean difference (MD) in GLS between CTRCD and non-CTRCD cohorts was −2.69 (95% CI −4.13 to −1.25) at visit one, −2.50 (95% CI −4.19 to −0.81) at visit two, and −3.86 (95% CI −7.30 to −0.42) at the final assessment. For LVEF), the mean differences between cohorts were 3.64 (95% CI −1.95 to 9.23) at visit one, 0.70 (95% CI −2.66 to 4.06) at visit two, and 8.13 (95% CI −4.04 to 20.30) at the final visit.

Evaluating the CTRCD cohort within this definition, the pooled mean change from baseline in GLS progressed from 2.19 (95% CI 1.36 to 3.02) at visit one to 2.86 (95% CI 1.43 to 4.28) at visit two, reaching 4.32 (95% CI −0.75 to 9.38) at visit three and 4.21 (95% CI 3.11 to 5.32) by the last visit. The mean change in LVEF declined, from −0.64 (95% CI −1.25 to −0.03) at visit one, −3.58 (95% CI −6.32 to −0.85) at visit two, −3.67 (95% CI −8.34 to 1.00) at visit three, and −5.83 (95% CI −8.99 to −2.68) at the final assessment.

In the non-CTRCD subgroup, the mean change from baseline in GLS was 0.64 (95% CI 0.05 to 1.23) at visit one, 0.10 (95% CI −1.03 to 1.23) at visit two, and 0.90 (95% CI 0.32 to 1.48) at the final visit. The changes in LVEF for this subgroup were −1.76 (95% CI −3.08 to −0.44) at visit one, −1.60 (95% CI −3.82 to 0.62) at visit two, and −2.18 (95% CI −3.47 to −0.88) at the last visit.

CMR-specific analyses for the guideline-based composite CTRCD definition subgroup in CTRCD patients found a mean GLS change of 2.70 (95% CI 1.72 to 3.68) at visit one and 2.80 (95% CI 1.79 to 3.81) at the final assessment. LVEF mean changes measured via CMR in this cohort were −0.70 (95% CI −3.70 to 2.30) at visit one and −0.20 (95% CI −3.07 to 2.67) at the last visit (Supplementary Figure S9).

## 4. Discussion

### 4.1. Main Findings

This systematic review and meta-analysis, encompassing 2083 patients across 33 observational studies, synthesises the available evidence on longitudinal imaging changes associated with CTRCD. Several key findings emerged. First, compared with non-CTRCD cohorts, patients with CTRCD showed greater worsening in GLS across the analysed follow-up intervals, supporting the role of serial strain assessment in detecting early functional change. Second, LVEF showed less consistent between-group discrimination at earlier timepoints, with clearer separation emerging later in follow-up. Third, in exploratory subgroup analyses, no statistically significant differences were detected between echocardiography-derived and CMR-derived measures of GLS or LVEF change, although the available CMR evidence base was limited. Fourth, study-level meta-regression did not identify significant associations between baseline mean GLS/LVEF and CTRCD event rates across studies.

### 4.2. CTRCD Incidence and Definition-Related Heterogeneity

The pooled CTRCD estimate of 27% suggests a substantial burden of treatment-associated cardiac dysfunction across contemporary oncology cohorts, particularly when both clinical and subclinical definitions are considered. A prior scoping review, which predominantly defined CTRCD using the traditional LVEF criterion, reported pooled prevalences of approximately 17% [50]. The higher 27% estimate observed here may reflect the additional burden of subclinical CTRCD—myocardial dysfunction detected exclusively by GLS criteria (typically a >15% relative decline), as reported in the 2022 ESC Guidelines on cardio-oncology [3]. This subclinical dysfunction is effectively rendered invisible to LVEF-only surveillance strategies [3]. Translated to population-level impact, for every 100 patients receiving chemotherapy-based regimens, approximately 27 will develop measurable cardiac dysfunction; a proportion that is likely to grow further as more sensitive monitoring expands and cancer survivorship continues to improve globally [51].

### 4.3. Interpretation of GLS and LVEF Findings

The greater deterioration in GLS observed in CTRCD cohorts supports the clinical value of serial strain imaging as an early marker of myocardial dysfunction during cancer therapy. Compared with non-CTRCD cohorts, patients who developed CTRCD showed more consistent worsening in GLS across follow-up intervals, whereas between-group differences in LVEF were less consistent at earlier timepoints and became more apparent later in follow-up. This pattern is biologically plausible, as abnormalities in myocardial deformation may precede measurable reductions in volumetric systolic function. These findings are consistent with prior evidence suggesting that GLS may identify subclinical left ventricular dysfunction before overt LVEF decline, particularly in patients receiving anthracyclines or trastuzumab [10], and with trial evidence supporting strain-guided surveillance during potentially cardiotoxic therapy [52].

The higher reproducibility of GLS compared with LVEF may also partly explain its greater sensitivity for early functional change [10]. However, GLS should be interpreted alongside LVEF, clinical risk profile, symptoms, biomarkers, and treatment exposure, rather than as an isolated replacement for conventional surveillance.

### 4.4. Imaging Modality Considerations

The absence of a statistically significant difference between echocardiography- and CMR-derived GLS in CTRCD patients is a key finding of this meta-analysis. CMR feature-tracking (CMR-FT) has been validated as a powerful technique for myocardial deformation analysis, offering operator-independent post-processing, superior signal-to-noise ratio, and the unique capacity for simultaneous characterisation of myocardial tissue properties through T1 mapping, T2 mapping, and late gadolinium enhancement [53]. Ananthapadmanabhan et al. (2021) demonstrated in a head-to-head modality comparison that CMR-FT and speckle-tracking echocardiography (STE) showed good inter-modality agreement for whole-layer GLS (correlation coefficients ranging from 0.660 to 0.687), with minimal systematic bias on Bland–Altman analysis, suggesting that no statistically significant difference was detected in the limited available comparative data [54]. From a health systems perspective, the absence of a statistically significant difference between echocardiography and CMR for serial GLS monitoring in CTRCD may have important resource implications. However, this finding should be interpreted cautiously, as the available evidence remains limited and underpowered. CMR remains expensive, has limited availability, and is contraindicated in patients with certain metallic implants. The present meta-analysis provides supporting evidence for the use of echocardiography as the workhorse modality for cardio-oncology strain surveillance. Given its accessibility, lower cost, and established role in surveillance pathways, echocardiography remains the pragmatic first-line modality for serial functional assessment, while CMR may be particularly useful when echocardiographic image quality is suboptimal or diagnostic uncertainty persists.

### 4.5. Baseline Function and Risk Interpretation

Our study-level meta-regression did not identify significant associations between baseline mean GLS or LVEF and CTRCD event rates across studies. While this finding should not be interpreted as patient-level prognostic evidence, it does support the practical principle that a normal baseline study does not eliminate the need for continued surveillance during treatment. This baseline paradox contrasts the clinical heuristic that a reassuringly normal pre-treatment echocardiogram justifies reduced surveillance intensity during therapy. Terluk et al. (2024) demonstrated that patients with high baseline GLS values may be incorrectly reassured and require re-stratification into a lower-risk group [55]. The 2022 ESC Guidelines on cardio-oncology explicitly recognise this by incorporating GLS change as a component of CTRCD definitions, thereby shifting the paradigm from static “threshold” assessment to dynamic “delta” surveillance [3]. The present meta-regression is consistent with this guidance, but it should be regarded as hypothesis-generating rather than confirmatory.

### 4.6. Clinical Implications

Collectively, these findings support serial GLS assessment alongside LVEF in patients receiving potentially cardiotoxic cancer therapy, particularly in those at higher baseline risk. GLS may help identify early functional deterioration before overt LVEF decline, but it should be interpreted together with LVEF, symptoms, biomarkers, treatment exposure, and the overall clinical risk profile [56]. Echocardiography remains a practical and accessible modality for longitudinal surveillance in many settings, although the comparative evidence with CMR remains limited and underpowered. CMR may therefore be most useful when echocardiographic image quality is suboptimal, when tissue characterisation is needed, or when diagnostic uncertainty remains. In practice, surveillance should include a comprehensive baseline assessment with echocardiographic LVEF and GLS, integrated with clinical risk stratification using validated tools such as the HFA-ICOS baseline risk score [3].

Since baseline study-level GLS and LVEF were not significantly associated with CTRCD event rates, normal baseline imaging should not be used alone to justify reducing surveillance intensity. Follow-up should instead remain guided by treatment exposure, baseline risk, symptoms, biomarkers, and dynamic imaging changes [55,56,57].

### 4.7. Strengths and Limitations

This meta-analysis has several notable methodological strengths. The synthesis spans 2083 patients from 33 studies across multiple tumour types, chemotherapeutic regimens, and imaging platforms, providing a clinically representative and generalisable dataset. Most distinctively, this analysis addresses questions that have been systematically underexplored in prior syntheses: the temporal discriminatory performance of GLS versus LVEF across sequential visits, the direct modality comparison of echo versus CMR for strain surveillance, and the meta-regression of baseline function indices against CTRCD incidence, which provides population-level evidence that directly informs guideline risk stratification frameworks.

Nevertheless, several limitations merit emphasis. First, CTRCD definitions varied substantially across studies, including LVEF-based, GLS-based, and composite definitions incorporating symptoms or biomarkers, which may have influenced pooled incidence estimates. Second, follow-up schedules varied across studies, and harmonising them into Visit 1, Visit 2, Visit 3, and Last Visit categories may have reduced temporal precision. Third, inter-vendor and inter-software variability in GLS measurement may limit the direct comparability of pooled strain values. Fourth, the CMR evidence base was small relative to the echocardiographic literature, limiting confidence in modality comparisons. Finally, the meta-regression analyses were conducted at the study level and should not be interpreted as patient-level prognostic models.

## 5. Conclusions

CTRCD represents a substantial burden in contemporary cancer care, with a pooled estimate of approximately 27% across published studies, although this figure is influenced by marked heterogeneity in populations, therapies, follow-up schedules, and outcome definitions. Across longitudinal analyses, deterioration in GLS appears earlier and more consistently than decline in LVEF, supporting the value of serial deformation imaging during treatment surveillance. However, baseline study-level mean GLS and LVEF were not significantly associated with CTRCD event rates, and the current evidence directly comparing echocardiography with CMR remains limited. Future studies should adopt standardised CTRCD definitions, harmonised follow-up intervals, and prospective head-to-head multimodality designs.

## Figures and Tables

**Table 1:** Baseline characteristics of included studies stratified by imaging modality.

*Panel A. Echocardiography-Only Studies (n = 27)*										
Study	Sample Size (N)	Design	Age (Years)	Female (%)	Baseline GLS (Echo)		Baseline LVEF (Echo)		Cancer Type	Cancer Therapy
AboElMagdRasheed et al. 2022 [19]	50	Prospective	45.3 ± 12.1	38 (76%)	−19.57 ± 1.66		65.7 ± 4.88		Breast/Lymphoma/Others	Anthracyclines
Allam et al. 2023 [20]	80	Prospective	51 ± 11	55 (68.8%)	-		-		Breast/Haematological/Colon/Others	Anthracyclines
Anqi et al. 2019 [21]	40	Prospective	47.3 ± 9.9	40 (100%)	−19.7 ± 2.46		69.76 ± 6.13		Breast	Anthracyclines
BenKridis et al. 2020 [22]	50	Prospective	49.6 ± 8.5	50 (100%)	−20.71 ± 2.30		62.48 ± 3.04		Breast (HER2-positive)	Trastuzumab ± Anthracyclines
Bhagat et al. 2023 [23]	31	Prospective	50 (46–55)	31 (100%)	−19.43 ± 2.56		62.66 ± 3.88		Breast	Anthracyclines
Caballero-Valderrama et al. 2024 [24]	33	Prospective	52.8 ± 10.2	33 (100%)	−21.73 ± 1.74		61.5 ± 4.9		Breast	Anthracyclines (Epirubicin)
Cadeddu et al. 2017 [25]	45	Prospective	51.4 ± 9.1	45 (100%)	−17.6 ± 1.3		62.8 ± 2.0		Breast	Anthracyclines + Trastuzumab
Chang et al. 2021 [26]	74	Prospective	57.9 ± 17.0	32 (43%)	−19.7 ± 2.5		69.1 ± 7.0		Lymphoma	Anthracyclines
Charbonnel et al. 2017 [27]	86	Prospective	48 (30–63.5)	31 (36%)	−21.16 ± 2.86		65.66 ± 5.27		Lymphoma/Leukaemia	Anthracyclines
DiLisi et al. 2023 [29]	61	Prospective	53 ± 9.4	61 (100%)	−20.36 ± 1.47		60.0 ± 3.21		Breast	Anthracyclines ± Trastuzumab
DiLisi et al. 2023 [30]	169	Prospective	55 ± 10.8	169 (100%)	−20.7 ± 2.1		60.0 ± 1.7		Breast	Anthracyclines ± Trastuzumab
El-Sherbeny et al. 2019 [31]	61	Prospective	47.4 ± 9.1	61 (100%)	-		-		Breast (HER2-positive)	Anthracyclines + Trastuzumab
Fallah-Rad et al. 2011 [7]	42	Prospective	47 ± 9	42 (100%)	-		-		Breast (HER2-positive)	Anthracyclines + Trastuzumab
Gripp et al. 2018 [32]	49	Prospective	49.7 ± 12.2	49 (100%)	-		-		Breast	Anthracyclines ± Trastuzumab
Guan et al. 2021 [33]	79	Prospective	48	79 (100%)	−22.6 ± 1.77		66.65 ± 4.13		Breast	Anthracyclines ± Trastuzumab
Hazim et al. 2023 [34]	47	Prospective	52 ± 12	47 (100%)	-		-		Breast (HER2-positive)	HER2-directed therapy ± Anthracyclines
Li et al. 2024 [38]	80	Prospective	54.1 ± 8.5	42 (52.5%)	−22.77 ± 2.45		61.3 ± 4.73		Lymphoma	Anthracyclines
Liu et al. 2022 [39]	67	Prospective	49.5 ± 6.0	67 (100%)	-		-		Breast	Anthracyclines or Trastuzumab
Mele et al. 2016 [40]	30	Prospective	53 ± 11	29 (97%)	−21.5 ± 1.7		63 ± 3		Breast	Anthracyclines ± Trastuzumab
Mornos et al. 2013 [41]	74	Prospective	51 ± 11	43 (58%)	−21.2 ± 2.5		61 ± 5.6		Breast/Lymphoma/Leukaemia/Osteosarcoma	Anthracyclines
Moya et al. 2023 [42]	50	Prospective	56 ± 12	50 (100%)	-		-		Breast	Anthracyclines ± Trastuzumab
Negishi et al. 2013 [43]	81	Prospective	50 ± 11	81 (100%)	-		-		Breast	Trastuzumab ± Anthracyclines
Park et al. 2020 [44]	72	Retrospective	49.0 ± 8.7	72 (100%)	−21.0 ± 2.4		65.0 ± 6.4		Breast (HER2-positive)	Anthracyclines + Trastuzumab
Stoodley et al. 2013 [45]	78	Prospective	52 ± 10	77 (99%)	−19.0 ± 2.3		58.0 ± 3.0		Breast (HER2-negative)	Anthracyclines
Wang et al. 2020 [47]	65	Prospective	51.3 ± 13.5	34 (52.3%)	−19.9 ± 3.2		61.2 ± 5.4		Diffuse large B-cell lymphoma	Anthracyclines (R-CHOP)
Yang et al. 2024 [48]	51	Prospective	57 (51–62)	8 (16%)	−21.8 ± 2.6		59.7 ± 6.5		Gastrointestinal (Gastric/Colorectal)	Fluorouracil-based chemotherapy
Zhang et al. 2022 [49]	207	Prospective	42 (20–69)	178 (86%)	−20.9 ± 2.34		60.92 ± 5.78		Breast/Lymphoma/Sarcoma	Anthracyclines
***Panel B. Cardiac magnetic resonance (CMR)-only studies (n = 4)***										
**Study**	**Sample size (N)**	**Design**	**Age (years)**	**Female (%)**	**Baseline GLS (CMR)**		**Baseline LVEF (CMR)**		**Cancer type**	**Cancer therapy**
Cheng et al. 2023 [28]	24	Prospective	47 ± 11	24 (100%)	−16.0 ± 1.6		63.7 ± 4.33		Breast (HER2-positive)	Anti-HER2 (Trastuzumab ± Pertuzumab)
Kersten et al. 2023 [14]	34	Prospective	50.2 ± 10.3	34 (100%)	−14.9 ± 2.1		65.2 ± 6.8		Breast	Anthracyclines
Lenihan et al. 2025 [37]	59	Prospective	54 ± 14	50 (85%)	−20.0 ± 2.0		61 ± 5		Breast/Lymphoma	Anthracyclines ± Trastuzumab
Tahir et al. 2022 [46]	66	Prospective	53 ± 13	66 (100%)	-		-		Breast	Epirubicin-based chemotherapy ± Radiotherapy
***Panel C. Studies reporting both echocardiography and Cardiac magnetic resonance (CMR) at baseline (n = 2)***										
**Study**	**Sample size (N)**	**Design**	**Age (years)**	**Female (%)**	**GLS (Echo)**	**LVEF (Echo)**	**GLS (CMR)**	**LVEF (CMR)**	**Cancer type**	**Cancer therapy**
Huynh et al. 2025 [35]	16	Prospective	61 (51–69.5)	16 (100%)	−21.8 ± 1.61	65.2 ± 6.19	−19.16 ± 2.71	72.5 ± 6.36	Breast	Cytotoxic chemotherapy (Anthracyclines ± Anti-HER2)
Kar et al. 2023 [36]	32	Prospective	59.4 ± 9.7	32 (100%)	−18.6 ± 2.6	58.5 ± 6.0	−18.8 ± 1.5	58.0 ± 5.6	Breast	Anthracyclines + Trastuzumab

**Table 2:** Study characteristics and CTRCD outcomes stratified by imaging modality.

*Panel A. Echocardiography-Only Studies (n = 27)*							
Study	Analyzed Population (N) *	Follow-Up (Months)	Imaging Modality	Subclinical CTRCD N (%)	Clinical CTRCD N (%)	Total CTRCD N (%)	Outcome CTRCD
AboElMagd et al. 2022 [19]	50	6	Echo	9 (18%)	4 (8%)	9 (18%)	Sub-Clinical/Clinical
Allam et al. 2023 [20]	80	3	Echo	-	10 (12.5%)	10 (12.5%)	Clinical
Anqi et al. 2019 [21]	40	-	Echo	18 (45%)	-	18 (45%)	Sub-Clinical
BenKridis et al. 2020 [22]	50	15	Echo	-	2 (4.0%)	7 (14.0%)	Clinical
Bhagat et al. 2023 [23]	26	6	Echo	10 (38.0%)	7 (26%)	10 (38.0%)	Sub-Clinical/Clinical
Caballero et al. 2024 [24]	33	12	Echo	9 (27.27%)	5 (15.15%)	9 (27.27%)	Sub-Clinical/Clinical
Cadeddu et al. 2017 [25]	45	12	Echo	-	6 (13.3%)	6 (13.3%)	Clinical
Chang et al. 2021 [26]	74	12	Echo	36 (49%)	-	36 (49%)	Sub-Clinical
Charbonnel et al. 2017 [27]	86	12	Echo	-	6 (7.0%)	6 (7.0%)	Clinical
DiLisi et al. 2023 [29]	61	6	Echo	23 (31%)	0 (0%)	23 (31%)	Sub-Clinical
DiLisi et al. 2023 [30]	169	6	Echo	28 (17%)	-	28 (17%)	Sub-Clinical
El-Sherbeny et al. 2019 [31]	61	12	Echo	-	18 (29.5%)	18 (29.5%)	Clinical
Fallah-Rad et al. 2011 [7]	42	12	Echo	-	10 (25%)	10 (25%)	Clinical
Gripp et al. 2018 [32]	49	12	Echo	-	5 (10%)	5 (10%)	Clinical
Guan et al. 2021 [33]	79	6	Echo	-	9 (11.4%)	9 (11.4%)	Clinical
Hazim et al. 2023 [34]	47	12	Echo	-	7 (14.9%)	7 (14.9%)	Clinical
Li et al. 2024 [38]	80	4	Echo	-	14 (17.5%)	14 (17.5%)	Clinical
Liu et al. 2022 [39]	67	-	Echo	52 (78%) **	-	52 (78%)	Sub-Clinical
Mele et al. 2016 [40]	27	-	Echo	6 (20%)	-	6 (20%)	Sub-Clinical
Mornos et al. 2013 [41]	74	12	Echo	-	10 (13.5%)	10 (13.5%)	Clinical
Moya et al. 2023 [42]	50	12	Echo	10 (20%)	9 (18%)	19 (38%)	Sub-Clinical/Clinical
Negishi et al. 2013 [43]	81	12	Echo	24 (30%)	-	24 (30%)	Clinical
Park et al. 2020 [44]	72	-	Echo	-	13 (18.1%)	13 (18.1%)	Clinical
Stoodley et al. 2013 [45]	45	12	Echo	8 (16%)	-	8 (16%)	Sub-Clinical
Wang et al. 2020 [47]	65	10	Echo	-	11 (16.9%)	11 (16.9%)	Clinical
Yang et al. 2024 [48]	51	-	Echo	-	-	6 (11.8%)	-
Zhang et al. 2022 [49]	145	6	Echo	69 (35.0%)	16 (8.8%)	69 (35.0%)	Sub-Clinical/Clinical
***Panel B. Cardiac magnetic resonance (CMR)-only studies (n = 4)***							
**Study**	**Analyzed** **population (N)**	**Follow-up (months)**	**Imaging Modality**	**Subclinical CTRCD N (%)**	**Clinical CTRCD N (%)**	**Total CTRCD N (%)**	**Outcome CTRCD**
Cheng et al. 2023 [28]	24	3	CMR	4 (16.7%)	2 (8.3%)	6 (25%)	Sub-Clinical/Clinical
Kersten et al. 2023 [14]	32	12	CMR	18 (56.3%)	-	18 (56.3%)	Sub-Clinical
Lenihan et al. 2025 [37]	59	12	CMR	24 (41%)	9 (15%)	24 (41%)	Sub-Clinical/Clinical
Tahir et al. 2022 [46]	66	13	CMR	-	-	9 (14%)	-
***Panel C. Studies reporting both echocardiography and Cardiac magnetic resonance (CMR) at baseline (n = 2)***							
**Study**	**Analyzed** **population (N)**	**Follow-up (months)**	**Imaging Modality**	**Subclinical CTRCD N (%)**	**Clinical CTRCD N (%)**	**Total CTRCD N (%)**	**Outcome CTRCD**
Huynh et al. 2025 [35]	11	6	Echo/CMR	-	-	-	-
Kar et al. 2023 [36]	32	6	Echo/CMR	-	-	9 (28.1%)	-

* Analyzed population (N) refers to the number of patients with available outcome data at the relevant follow-up time point and was used as the denominator for reported percentages; therefore, this may differ from the baseline sample size presented in Table 1. ** Calculated from Group A (anthracycline without trastuzumab), 29 (85.3%), and Group B (trastuzumab without anthracycline), 23 (69.7%). This total was calculated from both treatment groups and was not directly reported in the article as a single overall value. Abbreviations: CMR, cardiac magnetic resonance; CTRCD, cancer therapy-related cardiac dysfunction. In the table, - means there is no available data in the relevant study.

**Table 3:** Definitions of cardiotoxicity.

*Panel A. Echocardiography-Only Studies (n = 27)*		
Study	Definitions of Cardiotoxicity	Definition Category
AboElMagd et al. 2022 [19]	Drop in global longitudinal strain (GLS) ≥15% from baseline preceding a reduction in LVEF, with LVEF decline defined as ≥5% with symptoms or ≥10% without symptoms to <55%.	combined imaging definition
Allam et al. 2023 [20]	Anthracycline-related cardiac dysfunction per ESC 2022 guidelines: new LVEF reduction ≥10 percentage points to 40–49%, supported by GLS decrease and elevated hs-Troponin-I and NT-proBNP.	guideline-based composite
Anqi et al. 2019 [21]	Decrease in LVEF > 10% from the normal lower limit without heart failure symptoms, or >5% with symptoms.	LVEF-only
BenKridis et al. 2020 [22]	Asymptomatic LVEF decrease of 10–15% to <50% or >15% decrease in left ventricular longitudinal myocardial strain, or symptomatic heart failure with LVEF < 50% (ESC criteria).	guideline-based composite
Bhagat et al. 2023 [23]	Decrease in LVEF > 20% when the baseline LVEF is normal or a decrease in LVEF > 10% when the baseline LVEF is less than the institutional lower limit of normal a decrease in LVEF > 5% with an absolute LVEF < 55% and accompanying symptoms of clinical HF, or a decrease in LVEF > 10% with an absolute LVEF < 55% without clinical HF	LVEF-only
Caballero et al. 2024 [24]	Decrease in LVEF > 10% compared with baseline value, with final LVEF < 53%.	LVEF-only
Cadeddu et al. 2017 [25]	LVEF reduction ≥5% to <55% with symptoms or ≥10% to <55% without symptoms (Cardiac Review and Evaluation Committee criteria).	guideline-based composite
Chang et al. 2021 [26]	Relative reduction in left ventricular global longitudinal strain (GLS) ≥15% from baseline.	GLS-only
Charbonnel et al. 2017 [27]	Decrease in LVEF > 10 percentage points to <53%.	LVEF-only
DiLisi et al. 2023 [29]	Subclinical cardiac dysfunction defined as a relative decrease in GLS ≥ 12% from baseline; CTRCD defined as absolute LVEF decrease ≥10% to <50% or absolute LVEF decrease >20%	combined imaging definition
DiLisi et al. 2023 [30]	Asymptomatic mild CTRCD is defined as the presence of preserved LVEF ≥ 50% but a new relative decline in (GLS) > 15% from baseline and/or a new rise in cardiac biomarkers	imaging plus biomarkers
El-Sherbeny et al. 2019 [31]	EF reduction ≥5% to <55% with heart failure symptoms, or asymptomatic EF reduction ≥10% to <55%.	LVEF-only
Fallah-Rad et al. 2011 [7]	LVEF decline ≥10% to <55% with signs or symptoms of congestive heart failure requiring drug discontinuation.	LVEF-only
Gripp et al. 2018 [32]	LVEF reduction ≥5% to <55% with symptoms or ≥10% to <55% without symptoms (trastuzumab committee criteria).	guideline-based composite
Guan et al. 2021 [33]	Absolute LVEF reduction >5% to <53% with symptoms, or >10% to <53% without symptoms (ESC guidelines).	guideline-based composite
Hazim et al. 2023 [34]	Decrease in the left ventricular ejection fraction (LVEF) of >10% to a value <53%	LVEF-only
Li et al. 2024 [38]	New LVEF reduction ≥10 percentage points to ≤50% (ESC 2022), with additional assessment based on left atrial reservoir longitudinal strain (LASr), LV GLS, and composite LAVGLS.	guideline-based composite
Liu et al. 2022 [39]	CTRCD) defined as ≥15% absolute fall in LV global longitudinal strain (GLS) indicating subclinical myocardial toxicity; LVEF preserved or no significant early change	GLS-only
Mele et al. 2016 [40]	Relative reduction in GLS > 10% from baseline as marker of LV systolic dysfunction; no significant LV-EF change; reversibility of GLS alterations studied	GLS-only
Mornos et al. 2013 [41]	LVEF reduction ≥5% to <55% with symptoms or ≥10% to <55% without symptoms.	LVEF-only
Moya et al. 2023 [42]	Mild CTRCD: LVEF ≥ 50% with GLS decline >15%; Moderate CTRCD: LVEF < 50% with GLS decline >15%; Severe CTRCD: LVEF < 40%.	guideline-based composite
Negishi et al. 2013 [43]	EF decline >10% from baseline within 12 months, or symptomatic reduction of 5%, or asymptomatic reduction of 10% to EF < 55%.	LVEF-only
Park et al. 2020 [44]	LVEF decrease >10% from baseline to <55% following trastuzumab therapy.	LVEF-only
Stoodley et al. 2013 [45]	Subclinical left ventricular systolic dysfunction indicated by relative reduction in global longitudinal peak systolic strain ≥10%.	GLS-only
Wang et al. 2020 [47]	LVEF reduction >10% to <53%, confirmed by repeat echocardiography.	LVEF-only
Yang et al. 2024 [48]	LVEF decrease ≥5% to <53% with heart failure symptoms or ≥10% to <53% without symptoms; relative LV GLS decrease ≥15% from baseline.	combined imaging definition
Zhang et al. 2022 [49]	Subclinical ATRCD diagnosed as LVEF ≥ 50% with relative global longitudinal strain (GLS) decrease ≥15% and/or positive troponin-I; Clinical ATRCD diagnosed as LVEF decrease >10 percentage points to <50%	imaging plus biomarkers
***Panel B. Cardiac magnetic resonance (CMR)-only studies (n = 4)***		
**Study**	**Definitions of cardiotoxicity**	**Definition category**
Cheng et al. 2023 [28]	LVEF reduction >10% to <55% and/or GLS change >15%.	combined imaging definition
Kersten et al. 2023 [14]	Not reported.	Not reported
Lenihan et al. 2025 [37]	Asymptomatic LVEF decrease ≥10% with absolute value ≥53%, GLS decrease >15% from baseline, or abnormal cardiac biomarkers (troponin I, BNP, or NT-proBNP); absolute LVEF reduction ≥10% from baseline to <53% with heart failure symptoms or abnormal cardiac biomarkers.	imaging plus biomarkers
Tahir et al. 2022 [46]	LVEF decline ≥10% to <55% or GLS change >15% at FU2.	combined imaging definition
***Panel C. Studies reporting both echocardiography and Cardiac magnetic resonance (CMR) at baseline (n = 2)***		
**Study**	**Definitions of cardiotoxicity**	**Definition category**
Huynh et al. 2025 [35]	Not reported.	Not reported
Kar et al. 2023 [36]	Impaired global longitudinal strain (GLS) worsening >15% relative to baseline (International Cardio-Oncology Society and ASE-EACVI criteria).	guideline-based composite

**Table 4:** Meta-regression of baseline GLS and LVEF against the CTRCD event rate, reported as β (95% CI) and *p* values.

Variable	β	95% CI	*p* Value
Baseline GLS	0.16	[−0.09, 0.42]	0.195
Baseline EF	0.03	[−0.12, 0.19]	0.668

## Data Availability

No new data were created or analyzed in this study.

## Supplementary Materials

The following supporting information can be downloaded at: https://www.mdpi.com/article/10.3390/jcm15124520/s1.

## Abbreviations

Anti HER2	Anti-Human Epidermal Growth Factor Receptor 2
BNP	B-type natriuretic peptide
CHF	Congestive heart failure
CI	Confidence interval
CMR	Cardiac magnetic resonance (imaging)
CTRCD	Cancer therapy related cardiac dysfunction
GLS	Global longitudinal strain
LV	Left ventricle
LVEF	Left ventricular ejection fraction
RCT	Randomised controlled trial
