# Supplementary material for: Cancer Therapy-Related Cardiac Dysfunction: Pooled Incidence of Subclinical and Clinical Presentations Using Multimodal Multi-Parametric Imaging—A Systematic Review and Meta Analysis
Source: J Clin Med. 2026 Jun 11;15(12):4520. doi: 10.3390/jcm15124520 (PMC13301166; doi:10.3390/jcm15124520)

Supplementary Table S1: Search strategy in databases

Database	Search strategy	Number of records
**PubMed**	((((((global longitudinal strain) OR (GLS)) OR (circumferential strain)) OR (strain rate)) OR (Strain)) AND ((((((((((((((((cardiotoxicity) OR (cardiomyopathy)) OR (cancer therapy)) OR (chemotherapy)) OR (chemotherap*)) OR (trastuzumab)) OR (anthracycline)) OR (doxorubicin)) OR (adriamycin)) OR (idarubicin)) OR (epirubicin)) OR (daunorubicin)) OR (mitoxantrone)) OR (5-fluorouracil)) OR (paclitaxel)) OR (cyclophosphamide))) AND (((echocardiography) OR (cardiac magnetic resonance)) OR (CMR))	4,279
**Scopus†**	(TITLE-ABS-KEY (("global longitudinal strain" OR gls OR "circumferential strain" OR "strain rate" OR strain)) AND TITLE-ABS-KEY ((cardiotoxicity OR cardiomyopathy OR "cancer therapy" OR chemotherapy OR chemotherap* OR trastuzumab OR anthracycline OR doxorubicin OR adriamycin OR idarubicin OR epirubicin OR daunorubicin OR mitoxantrone OR 5-fluorouracil OR paclitaxel OR cyclophosphamide)) AND TITLE-ABS-KEY (("echocardiography" OR "cardiac magnetic resonance" OR "CMR")))	4,893
**Web of Science**	("global longitudinal strain" OR GLS OR "circumferential strain" OR "strain rate" OR Strain) (All Fields) and (cardiotoxicity OR cardiomyopathy OR "cancer therapy" OR chemotherapy OR chemotherap* OR trastuzumab OR anthracycline OR doxorubicin OR adriamycin OR idarubicin OR epirubicin OR daunorubicin OR mitoxantrone OR 5-fluorouracil OR paclitaxel OR cyclophosphamide) (All Fields) and ("echocardiography" OR "cardiac magnetic resonance" OR "CMR") (All Fields)	4,095
**Embase**	('global longitudinal strain':ti,ab,kw OR gls:ti,ab,kw OR 'circumferential strain':ti,ab,kw OR 'strain rate':ti,ab,kw OR strain:ti,ab,kw) AND (cardiotoxicity:ti,ab,kw OR cardiomyopathy:ti,ab,kw OR 'cancer therapy':ti,ab,kw OR chemotherapy:ti,ab,kw OR chemotherap*:ti,ab,kw OR trastuzumab:ti,ab,kw OR anthracycline:ti,ab,kw OR doxorubicin:ti,ab,kw OR adriamycin:ti,ab,kw OR idarubicin:ti,ab,kw OR epirubicin:ti,ab,kw OR daunorubicin:ti,ab,kw OR mitoxantrone:ti,ab,kw OR '5 fluorouracil':ti,ab,kw OR paclitaxel:ti,ab,kw OR cyclophosphamide:ti,ab,kw) AND ('echocardiography':ti,ab,kw OR 'cardiac magnetic resonance':ti,ab,kw OR 'cmr':ti,ab,kw)	5,586
**Cochrane**	((((((global longitudinal strain) OR (GLS)) OR (circumferential strain)) OR (strain rate)) OR (Strain)) AND ((((((((((((((((cardiotoxicity) OR (cardiomyopathy)) OR (cancer therapy)) OR (chemotherapy)) OR (chemotherap*)) OR (trastuzumab)) OR (anthracycline)) OR (doxorubicin)) OR (adriamycin)) OR (idarubicin)) OR (epirubicin)) OR (daunorubicin)) OR (mitoxantrone)) OR (5-fluorouracil)) OR (paclitaxel)) OR (cyclophosphamide))) AND (((echocardiography) OR (cardiac magnetic resonance)) OR (CMR))	293

†Searched using title, abstract and keyword

Supplementary Table S2. Baseline imaging and follow-up characteristics of included studies stratified by imaging modality.

<table>
<tr><td colspan="8">Panel A. Echocardiography-only studies (n = 27)</td></tr>
<tr><th>Study</th><th>Design</th><th>Follow-up type</th><th>Follow-up (months)</th><th colspan="2">Baseline GLS (Echo)</th><th colspan="2">Baseline LVEF (Echo)</th></tr>
<tr><td>AboElMagd et al. 2022 [19]</td><td>Prospective</td><td>Short-term</td><td>6</td><td colspan="2">−19.57 ± 1.66</td><td colspan="2">65.7 ± 4.88</td></tr>
<tr><td>Allam et al. 2023 [20]</td><td>Prospective</td><td>Short-term</td><td>3</td><td colspan="2">-</td><td colspan="2">-</td></tr>
<tr><td>Anqi et al. 2019 [21]</td><td>Prospective</td><td>-</td><td>-</td><td colspan="2">−19.7 ± 2.46</td><td colspan="2">69.76 ± 6.13</td></tr>
<tr><td>BenKridis et al. 2020 [22]</td><td>Prospective</td><td>Long-term</td><td>15</td><td colspan="2">−20.71 ± 2.30</td><td colspan="2">62.48 ± 3.04</td></tr>
<tr><td>Bhagat et al. 2023 [23]</td><td>Prospective</td><td>Short-term</td><td>6</td><td colspan="2">−19.43 ± 2.56</td><td colspan="2">62.66 ± 3.88</td></tr>
<tr><td>Caballero et al. 2024 [24]</td><td>Prospective</td><td>Long-term</td><td>12</td><td colspan="2">−21.73 ± 1.74</td><td colspan="2">61.5 ± 4.9</td></tr>
<tr><td>Cadeddu et al. 2017 [25]</td><td>Prospective</td><td>Long-term</td><td>12</td><td colspan="2">−17.6 ± 1.3</td><td colspan="2">62.8 ± 2.0</td></tr>
<tr><td>Chang et al. 2021 [26]</td><td>Prospective</td><td>Long-term</td><td>12</td><td colspan="2">−19.7 ± 2.5</td><td colspan="2">69.1 ± 7.0</td></tr>
<tr><td>Charbonnel et al. 2017 [27]</td><td>Prospective</td><td>Long-term</td><td>12</td><td colspan="2">−21.16 ± 2.86</td><td colspan="2">65.66 ± 5.27</td></tr>
<tr><td>DiLisi et al. 2023 [29]</td><td>Prospective</td><td>Short-term</td><td>6</td><td colspan="2">−20.36 ± 1.47</td><td colspan="2">60.0 ± 3.21</td></tr>
<tr><td>DiLisi et al. 2023 [30]</td><td>Prospective</td><td>Short-term</td><td>6</td><td colspan="2">−20.7 ± 2.1</td><td colspan="2">60.0 ± 1.7</td></tr>
<tr><td>El-Sherbeny et al. 2019 [31]</td><td>Prospective</td><td>Long-term</td><td>12</td><td colspan="2">-</td><td colspan="2">-</td></tr>
<tr><td>Fallah-Rad et al. 2011 [7]</td><td>Prospective</td><td>Long-term</td><td>12</td><td colspan="2">-</td><td colspan="2">-</td></tr>
<tr><td>Gripp et al. 2018 [32]</td><td>Prospective</td><td>Long-term</td><td>12</td><td colspan="2">-</td><td colspan="2">-</td></tr>
<tr><td>Guan et al. 2021 [33]</td><td>Prospective</td><td>Short-term</td><td>6</td><td colspan="2">−22.6 ± 1.77</td><td colspan="2">66.65 ± 4.13</td></tr>
<tr><td>Hazim et al. 2023 [34]</td><td>Prospective</td><td>Long-term</td><td>12</td><td colspan="2">-</td><td colspan="2">-</td></tr>
<tr><td>Li et al. 2024 [38]</td><td>Prospective</td><td>Short-term</td><td>4</td><td colspan="2">−22.77 ± 2.45</td><td colspan="2">61.3 ± 4.73</td></tr>
<tr><td>Liu et al. 2022 [39]</td><td>Prospective</td><td>-</td><td>-</td><td colspan="2">-</td><td colspan="2">-</td></tr>
<tr><td>Mele et al. 2016 [40]</td><td>Prospective</td><td>-</td><td>-</td><td colspan="2">−21.5 ± 1.7</td><td colspan="2">63 ± 3</td></tr>
<tr><td>Mornos et al. 2013 [41]</td><td>Prospective</td><td>Long-term</td><td>12</td><td colspan="2">−21.2 ± 2.5</td><td colspan="2">61 ± 5.6</td></tr>
<tr><td>Moya et al. 2023 [42]</td><td>Prospective</td><td>Long-term</td><td>12</td><td colspan="2">-</td><td colspan="2">-</td></tr>
<tr><td>Negishi et al. 2013 [43]</td><td>Prospective</td><td>Long-term</td><td>12</td><td colspan="2">-</td><td colspan="2">-</td></tr>
<tr><td>Park et al. 2020 [44]</td><td>Retrospective</td><td>-</td><td>-</td><td colspan="2">−21.0 ± 2.4</td><td colspan="2">65.0 ± 6.4</td></tr>
<tr><td>Stoodley et al. 2013 [45]</td><td>Prospective</td><td>Long-term</td><td>12</td><td colspan="2">−19.0 ± 2.3</td><td colspan="2">58.0 ± 3.0</td></tr>
<tr><td>Wang et al. 2020 [47]</td><td>Prospective</td><td>Short-term</td><td>10</td><td colspan="2">−19.9 ± 3.2</td><td colspan="2">61.2 ± 5.4</td></tr>
<tr><td>Yang et al. 2024 [48]</td><td>Prospective</td><td>-</td><td>-</td><td colspan="2">−21.8 ± 2.6</td><td colspan="2">59.7 ± 6.5</td></tr>
<tr><td>Zhang et al. 2022 [49]</td><td>Prospective</td><td>Short-term</td><td>6</td><td colspan="2">−20.9 ± 2.34</td><td colspan="2">60.92 ± 5.78</td></tr>
<tr><td colspan="8">Panel B. Cardiac magnetic resonance (CMR)-only studies (n = 4)</td></tr>
<tr><th>Study</th><th>Design</th><th>Follow-up Type</th><th>Follow-up (months)</th><th colspan="2">Baseline GLS (CMR)</th><th colspan="2">Baseline LVEF (CMR)</th></tr>
<tr><td>Cheng et al. 2023 [28]</td><td>Prospective</td><td>Short-term</td><td>3</td><td colspan="2">−16.0 ± 1.6</td><td colspan="2">63.7 ± 4.33</td></tr>
<tr><td>Kersten et al. 2023 [14]</td><td>Prospective</td><td>Long-term</td><td>12</td><td colspan="2">−14.9 ± 2.1</td><td colspan="2">65.2 ± 6.8</td></tr>
<tr><td>Lenihan et al. 2025 [37]</td><td>Prospective</td><td>Long-term</td><td>12</td><td colspan="2">−20.0 ± 2.0</td><td colspan="2">61 ± 5</td></tr>
<tr><td>Tahir et al. 2022 [46]</td><td>Prospective</td><td>Long-term</td><td>13</td><td colspan="2">-</td><td colspan="2">-</td></tr>
<tr><td colspan="8">Panel C. Studies reporting both echocardiography and Cardiac magnetic resonance (CMR) at baseline (n = 2)</td></tr>
<tr><th>Study</th><th>Design</th><th>Follow-up Type</th><th>Follow-up (months)</th><th>Baseline GLS (Echo)</th><th>Baseline LVEF (Echo)</th><th>Baseline GLS (CMR)</th><th>Baseline LVEF (CMR)</th></tr>
<tr><td>Huynh et al. 2025 [35]</td><td>Prospective</td><td>Short-term</td><td>6</td><td>−21.8 ± 1.61</td><td>65.2 ± 6.19</td><td>−19.16 ± 2.71</td><td>72.5 ± 6.36</td></tr>
<tr><td>Kar et al. 2023 [36]</td><td>Prospective</td><td>Short-term</td><td>6</td><td>−18.6 ± 2.6</td><td>58.5 ± 6.0</td><td>−18.8 ± 1.5</td><td>58.0 ± 5.6</td></tr>
</table>

- A dash indicates that data were not available in the original study.

Supplementary Figure S1: Risk of bias assessment of the included studies using ROBINS-I.

Study ID	Confounding	Classification of Interventions	Selection into the Study	Deviations from Intended Interventions	Missing Data	Measurement of Outcomes	Selection of Reported Result	Overall Risk
AboElMagdRasheed et al. 2022	✓	✓	✓	✓	✓	✓	✓	✓
Allam et al. 2023	✓	✓	✓	✓	✓	✓	✓	✓
Anqi Y et al. 2019	✓	✓	✓	✓	✓	✓	✓	✓
BenKridis et al. 2020	✓	✓	✓	✓	✓	✓	✓	✓
Bhagat, 2023	✓	✓	✓	✓	✓	✓	✓	✓
Caballero et al. 2024	✗	✓	✓	✓	✓	✓	✓	✗
Cadeddu et al. 2017	✓	✓	✓	✓	✓	✓	✓	✓
Chang et al. 2021	✓	✓	✓	✓	✓	✓	✓	✓
Charbonnel 2017	✓	✓	✓	✓	✓	✓	✓	✓
Cheng et al., 2023	✓	✓	✓	✓	✓	✓	✓	✓
Di Lisi et al. 2023	✓	✓	✓	✓	✓	✓	✓	✓
Di Lisi et al. 2023	✓	✓	✓	✓	✓	✓	✓	✓
El-Sherbeny et al. 2019	✓	✓	✓	✓	✓	✓	✓	✓
Fallah-Rad et al. 2011	✓	✓	✓	✓	✓	✓	✓	✓
Gripp et al. 2018	✓	✓	✓	✓	✓	✓	✓	✓
Guan et al. 2021	✓	✓	✓	✓	✓	✓	✓	✓
Hazim et al. 2023	✓	✓	✓	✓	✓	✓	✓	✓
Huynh et al. 2025	✓	✓	✓	✓	✓	✓	✓	✓
Kar 2023	✓	✓	✓	✓	✓	✓	✓	✓
Kersten et al., 2024	✓	✓	✓	✓	✓	✓	✓	✓
Lenihan et al. 2025	✓	✓	✓	✓	✓	✓	✓	✓
Li et al. ,2024	✓	✓	✓	✓	✓	✓	✓	✓
Liu et al.2022	✓	✓	✓	✓	✓	✓	✓	✓
Mele et al. 2016	✓	✓	✓	✓	✓	✓	✓	✓
Mornos et al 2013	✓	✓	✓	✓	✓	✓	✓	✓
Moya 2023	✓	✓	✓	✓	✓	✓	✓	✓
Negishi 2013	✓	✓	✓	✓	✓	✓	✓	✓
Park et al., 2020	!	✓	✓	✓	!	✓	✓	!
Stoodley et al. 2013	✓	✓	✓	✓	✓	✓	✓	✓
Tahir 2022	✓	✓	✓	✓	✓	✓	✓	✓
Wang 2020	✓	✓	✓	✓	✓	✓	✓	✓
Yang, 2024	✓	✓	✓	✓	✓	✓	✓	✓
Zhang et al. 2022	✓	✓	✓	✓	!	✓	✓	!

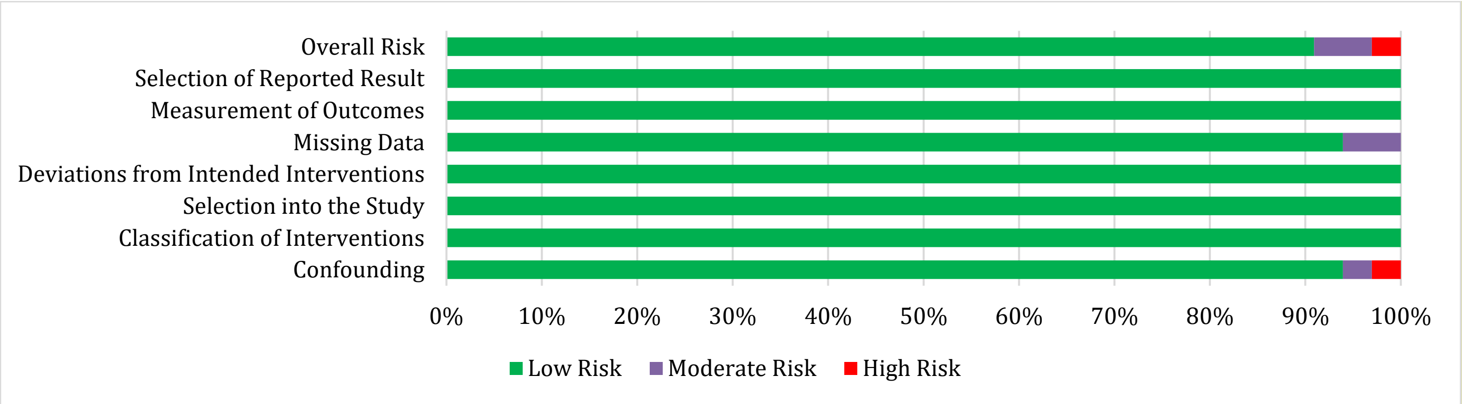

Supplementary Figure S2: Change in GLS from baseline with and without CTRCD by follow-up duration.

(2A) Change in GLS at visit 1 in patients with and without CTRCD

Study or Subgroup	CTRCD Mean	SD	Total	Non-CTRCD Mean	SD	Total	Weight	Mean Difference IV, Random, 95% CI
**Follow_up = Long Term**								
El□Sherbeny et al. 2019 (CTRCD, Clinical)	-3.18	0.94	18	-0.32	1.40	43	12.9%	-2.86 [-3.46; -2.26]
Charbonnel et al. 2017 (CTRCD, Sub-Clinical)	0.00	1.71	6	0.00	2.56	80	9.5%	0.00 [-1.48; 1.48]
Chang et al. 2021 (CTRCD, Subclinical)	-2.60	2.46	36	-0.10	2.42	38	11.0%	-2.50 [-3.61; -1.39]
Fallah-Rad et al. 2011 (CTRCD, Clinical)	-3.40	1.57	10	-0.30	2.35	32	10.4%	-3.10 [-4.37; -1.83]
Negishi et al. 2013 (CTRCD, Sub-Clinical)	-2.40	2.39	24	-0.30	2.00	57	11.1%	-2.10 [-3.19; -1.01]
Caballero-Valderrama et al. 2024 (CTRCD, Clinical)	-1.34	1.31	5	-0.43	2.14	28	9.8%	-0.91 [-2.30; 0.48]
**Total (95% CI)**			**99**			**278**	**64.7%**	**-2.01 [-3.23; -0.78]**
Heterogeneity: Tau^2 = 0.9269; Chi^2 = 18.16, df = 5 (P = 0.0028); I^2 = 72.5%								
**Follow_up = not mentioned**								
Moya et al. 2023 (Mild CTRCD, Clinical)	-1.20	3.12	10	0.90	2.55	16	6.6%	-2.10 [-4.40; 0.20]
Gripp et al. 2018 (CTRCD, Sub-Clinical)	-4.10	1.74	5	-0.90	2.05	44	8.8%	-3.20 [-4.84; -1.56]
**Total (95% CI)**			**15**			**60**	**15.4%**	**-2.83 [-9.44; 3.78]**
Heterogeneity: Tau^2 = 0; Chi^2 = 0.58, df = 1 (P = 0.4464); I^2 = 0%								
**Follow_up = Short Term**								
Di Lisi 2023 (CTRCD, Sub-Clinical)	-4.90	1.90	28	-0.40	2.23	141	12.2%	-4.50 [-5.29; -3.71]
Allam et al. 2023 (CTRCD, Sub-Clinical)	-3.70	2.92	10	-1.30	3.15	70	7.7%	-2.40 [-4.35; -0.45]
**Total (95% CI)**			**38**			**211**	**19.9%**	**-3.65 [-16.75; 9.45]**
Heterogeneity: Tau^2 = 1.6255; Chi^2 = 3.81, df = 1 (P = 0.0511); I^2 = 73.7%								
**Total (95% CI)**			**152**			**549**	**100.0%**	**-2.44 [-3.35; -1.53]**

Heterogeneity: Tau^2 = 1.2395; Chi^2 = 41.31, df = 9 (P < 0.0001); I^2 = 78.2%
Test for overall effect: t_9 = -6.07 (P = 0.0002)
Test for subgroup differences: Chi^2 = 2.70, df = 2 (P = 0.2593)

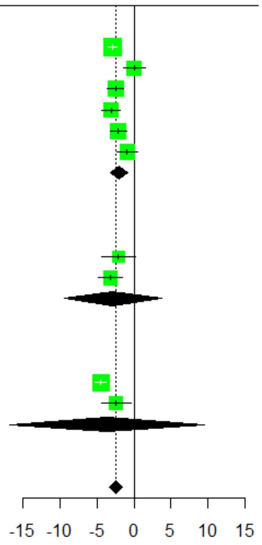

(2B) Change in GLS at visit 2 in patients with and without CTRCD

Study or Subgroup	CTRCD Mean	SD	Total	Non-CTRCD Mean	SD	Total	Weight	Mean Difference IV, Random, 95% CI
**Follow_up = Long Term**								
El□Sherbeny et al. 2019 (CTRCD, Clinical)	-5.33	1.05	18	-0.81	1.36	43	25.5%	-4.52 [-5.15; -3.89]
Charbonnel et al. 2017 (CTRCD, Sub-Clinical)	-1.56	2.41	6	-0.13	2.37	80	10.7%	-1.43 [-3.43; 0.57]
Chang et al. 2021 (CTRCD, Subclinical)	-3.80	2.42	36	0.30	2.49	38	19.2%	-4.10 [-5.22; -2.98]
Fallah-Rad et al. 2011 (CTRCD, Clinical)	-4.40	1.80	10	-0.80	2.62	32	15.5%	-3.60 [-5.04; -2.16]
Caballero-Valderrama et al. 2024 (CTRCD, Clinical)	-4.62	1.43	5	-0.88	1.68	28	15.9%	-3.74 [-5.14; -2.34]
**Total (95% CI)**			**75**			**221**	**86.9%**	**-3.77 [-5.04; -2.51]**
Heterogeneity: Tau^2 = 0.4438; Chi^2 = 9.28, df = 4 (P = 0.0546); I^2 = 56.9%								
**Follow_up = not mentioned**								
Moya et al. 2023 (Mild CTRCD, Clinical)	-2.60	2.04	10	-0.10	2.29	16	13.1%	-2.50 [-4.19; -0.81]
**Total (95% CI)**			**85**			**237**	**100.0%**	**-3.58 [-4.69; -2.47]**

Heterogeneity: Tau^2 = 0.5720; Chi^2 = 12.44, df = 5 (P = 0.0293); I^2 = 59.8%
Test for overall effect: t_5 = -8.30 (P = 0.0004)
Test for subgroup differences: Chi^2 = 1.70, df = 1 (P = 0.1923)

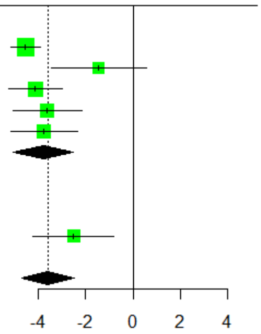

(2C) Change in GLS at visit 3 in patients with and without CTRCD

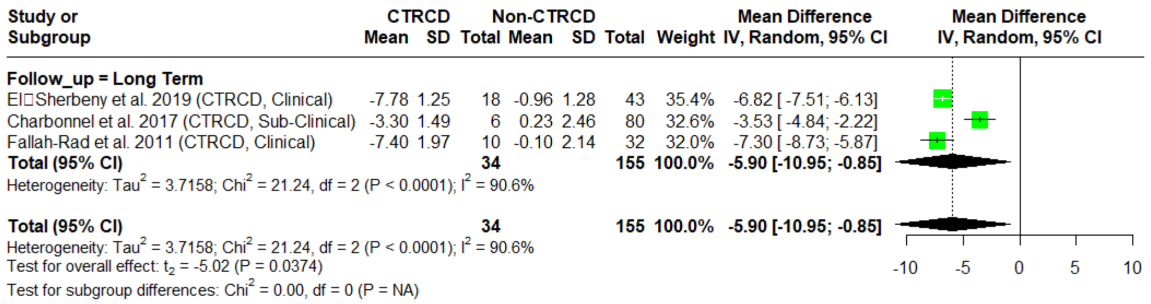

(2D) Change in GLS at the last visit in patients with and without CTRCD

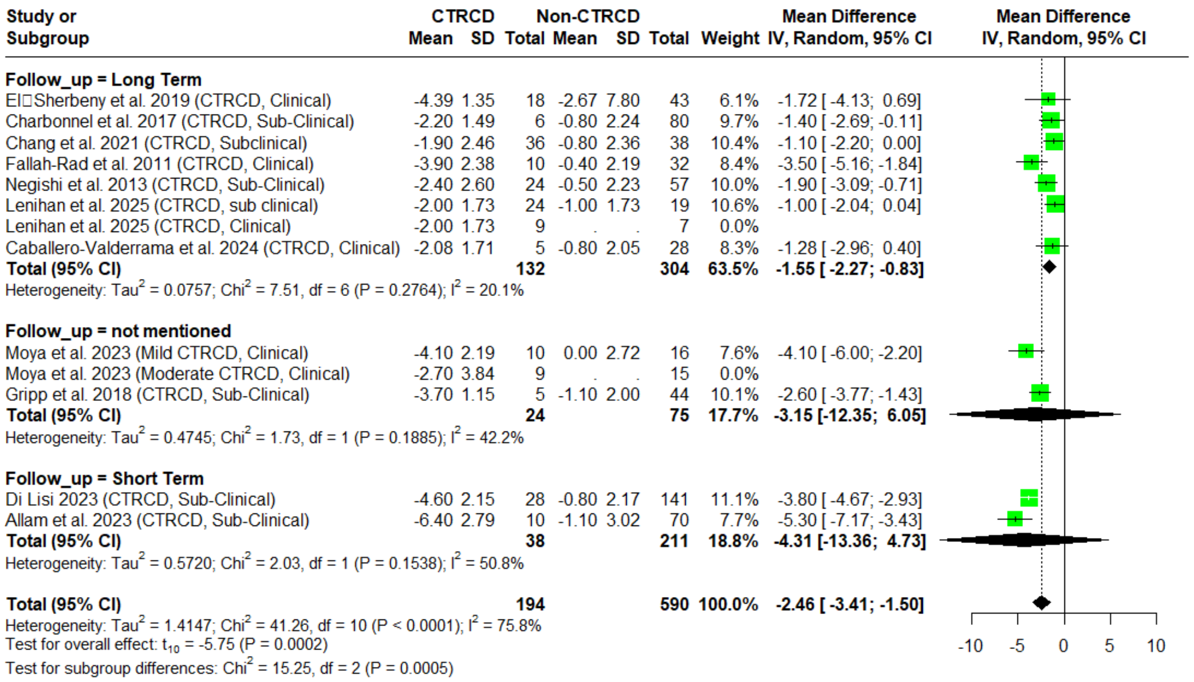

Supplementary Figure S2. Change in GLS from baseline with and without CTRCD by follow-up duration. (A) Change in GLS at visit 1 in patients with and without CTRCD. (B) Change in GLS at visit 2 in patients with and without CTRCD. (C) Change in GLS at visit 3 in patients with and without CTRCD. (D) Change in GLS at the last visit in patients with and without CTRCD [7,19,20,24,26,27,29,31,32,37,38,42,43,45,47].

Supplementary Figure S3: Change in LVEF from baseline with and without CTRCD by follow-up duration.

(3A) Change in LVEF at visit 1 in patients with and without CTRCD

Study or Subgroup	CTRCD Mean	CTRCD SD	CTRCD Total	Non-CTRCD Mean	Non-CTRCD SD	Non-CTRCD Total	Weight	Mean Difference IV, Random, 95% CI
**Follow_up = Long Term**								
El□Sherbeny et al. 2019 (CTRCD, Clinical)	3.17	2.52	18	1.17	2.92	43	18.2%	2.00 [0.54; 3.46]
Charbonnel et al. 2017 (CTRCD, Sub-Clinical)	0.03	2.86	6	-0.34	4.94	80	11.1%	0.37 [-2.16; 2.90]
Chang et al. 2021 (CTRCD, Subclinical)	0.90	8.85	36	-3.20	7.62	38	6.4%	4.10 [0.33; 7.87]
Fallah-Rad et al. 2011 (CTRCD, Clinical)	6.00	3.61	10	2.00	7.00	32	7.8%	4.00 [0.70; 7.30]
Negishi et al. 2013 (CTRCD, Sub-Clinical)	6.00	5.11	24	2.00	3.65	57	12.6%	4.00 [1.75; 6.25]
Caballero-Valderrama et al. 2024 (CTRCD, Clinical)	2.30	3.32	5	0.50	4.65	28	7.6%	1.80 [-1.58; 5.18]
**Total (95% CI)**			**99**			**278**	**63.8%**	**2.50 [0.96; 4.05]**
Heterogeneity: $Tau^2 = 0.5592$; $Chi^2 = 6.5$, df = 5 (P = 0.2606); $I^2 = 23.1\%$								
**Follow_up = not mentioned**								
Moya et al. 2023 (Mild CTRCD, Clinical)	1.60	4.06	10	0.10	4.94	16	7.2%	1.50 [-1.99; 4.99]
Gripp et al. 2018 (CTRCD, Sub-Clinical)	6.40	10.74	5	1.10	7.14	44	1.2%	5.30 [-4.35; 14.95]
**Total (95% CI)**			**15**			**60**	**8.5%**	**1.94 [-13.52; 17.40]**
Heterogeneity: $Tau^2 = 0$; $Chi^2 = 0.53$, df = 1 (P = 0.4678); $I^2 = 0\%$								
**Follow_up = Short Term**								
Di Lisi 2023 (CTRCD, Sub-Clinical)	0.90	3.50	28	0.00	2.10	141	19.2%	0.90 [-0.44; 2.24]
Allam et al. 2023 (CTRCD, Sub-Clinical)	8.20	4.49	10	3.00	5.71	70	8.6%	5.20 [2.11; 8.29]
**Total (95% CI)**			**38**			**211**	**27.8%**	**2.82 [-24.34; 29.97]**
Heterogeneity: $Tau^2 = 7.7705$; $Chi^2 = 6.27$, df = 1 (P = 0.0123); $I^2 = 84.1\%$								
**Total (95% CI)**			**152**			**549**	**100.0%**	**2.42 [1.23; 3.61]**

Heterogeneity: $Tau^2 = 1.1666$; $Chi^2 = 14.41$, df = 9 (P = 0.1084); $I^2 = 37.5\%$
Test for overall effect: $t_9 = 4.60$ (P = 0.0013)
Test for subgroup differences: $Chi^2 = 0.21$, df = 2 (P = 0.9006)

Mean Difference IV, Random, 95% CI

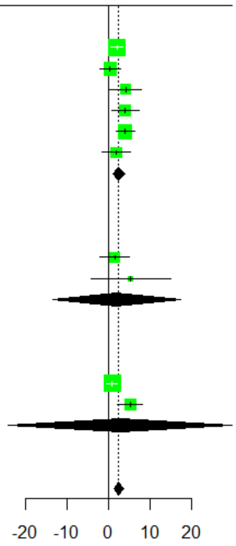

(3B) Change in LVEF at visit 2 in patients with and without CTRCD

Study or Subgroup	CTRCD Mean	CTRCD SD	CTRCD Total	Non-CTRCD Mean	Non-CTRCD SD	Non-CTRCD Total	Weight	Mean Difference IV, Random, 95% CI
**Follow_up = Long Term**								
El□Sherbeny et al. 2019 (CTRCD, Clinical)	17.06	3.24	18	0.52	3.03	43	17.2%	16.54 [14.79; 18.29]
Charbonnel et al. 2017 (CTRCD, Sub-Clinical)	2.67	4.15	6	0.66	4.94	80	16.8%	2.01 [-1.48; 5.50]
Chang et al. 2021 (CTRCD, Subclinical)	1.60	7.43	36	-2.70	7.11	38	16.8%	4.30 [0.98; 7.62]
Fallah-Rad et al. 2011 (CTRCD, Clinical)	22.00	7.94	10	-2.00	4.58	32	16.0%	24.00 [18.83; 29.17]
Caballero-Valderrama et al. 2024 (CTRCD, Clinical)	4.00	4.64	5	-0.20	5.00	28	16.4%	4.20 [-0.27; 8.67]
**Total (95% CI)**			**75**			**221**	**83.2%**	**10.15 [-1.68; 21.99]**
Heterogeneity: $Tau^2 = 85.9573$; $Chi^2 = 110.41$, df = 4 (P < 0.0001); $I^2 = 96.4\%$								
**Follow_up = not mentioned**								
Moya et al. 2023 (Mild CTRCD, Clinical)	2.30	4.12	10	1.60	4.46	16	16.8%	0.70 [-2.66; 4.06]
**Total (95% CI)**			**85**			**237**	**100.0%**	**8.56 [-1.25; 18.38]**

Heterogeneity: $Tau^2 = 82.7730$; $Chi^2 = 148.38$, df = 5 (P < 0.0001); $I^2 = 96.6\%$
Test for overall effect: $t_5 = 2.24$ (P = 0.0750)
Test for subgroup differences: $Chi^2 = 4.23$, df = 1 (P = 0.0397)

Mean Difference IV, Random, 95% CI

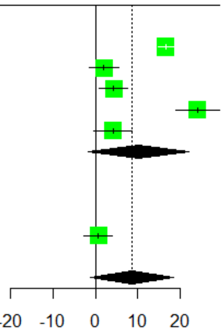

(3C) Change in LVEF at visit 3 in patients with and without CTRCD

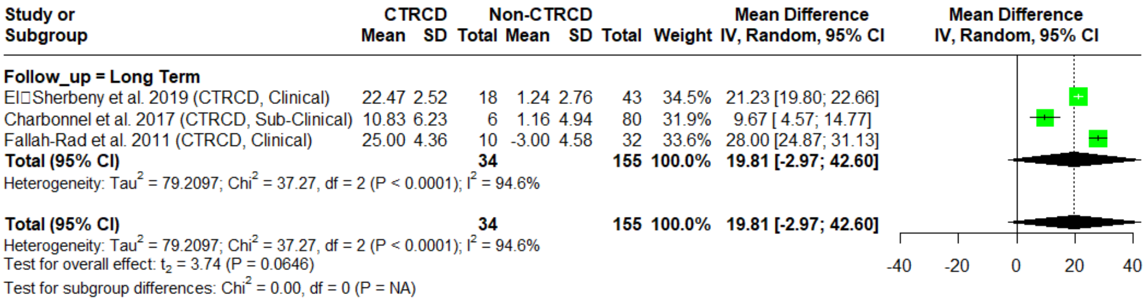

(3D) Change in at last visit in patients with and without CTRCD

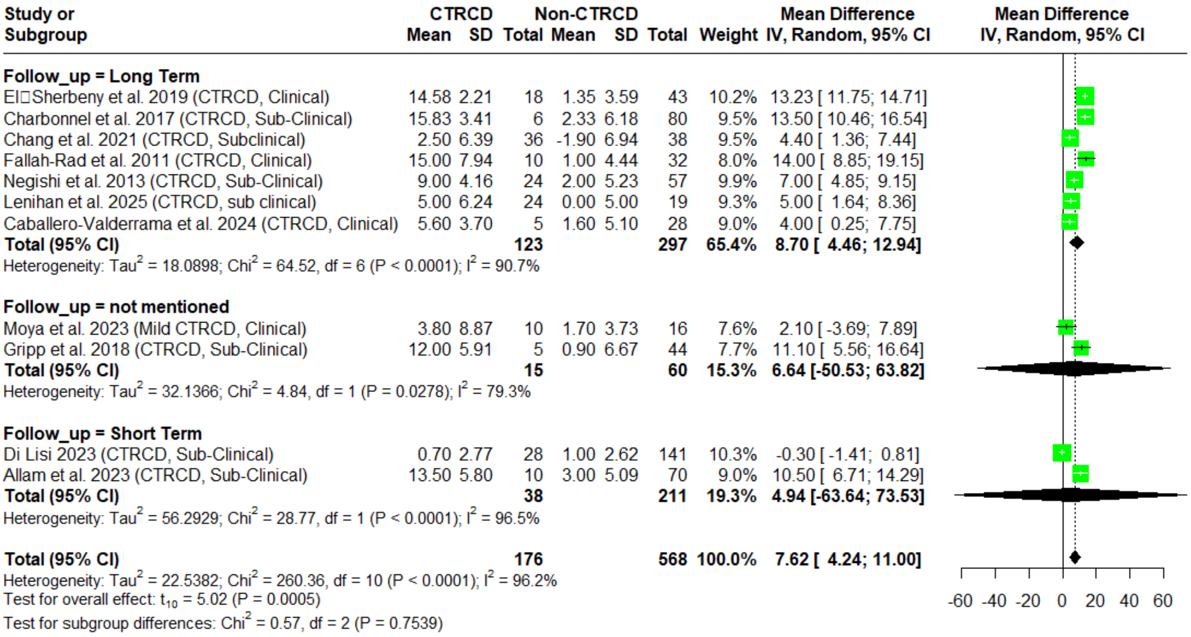

Supplementary Figure S3. Change in LVEF from baseline with and without CTRCD by follow-up duration. (A) Change in LVEF at visit 1 in patients with and without CTRCD. (B) Change in LVEF at visit 2 in patients with and without CTRCD. (C) Change in LVEF at visit 3 in patients with and without CTRCD. (D) Change in LVEF at the last visit in patients with and without CTRCD [7,20,24,26,27,29,31,32,37,42,43].

Supplementary Figure S4. Forest plot showing the leave-one-out sensitivity analysis of the pooled incidence of cancer therapy–related cardiac dysfunction (CTRCD) using a random-effects (inverse-variance) model.

Study	Tau2	Tau	I2	Proportion	95%-CI
Omitting El□Sherbeny et al. 2019 (Breast cancer, Trastuzumab)	0.8801	0.9381	88.3%	0.27	[0.20; 0.36]
Omitting Di Lisi 2023 (Breast cancer, Anthracycline followed by taxane + trastuzumab)	0.8653	0.9302	87.7%	0.27	[0.20; 0.36]
Omitting Moya et al. 2023 (Breast Cancer, anthracycline therapy w/wo Trastuzumab)	0.8681	0.9317	88.3%	0.27	[0.19; 0.35]
Omitting Charbonnel et al. 2017 (Lymphoma or Leukaemia, Anthracyclines)	0.7874	0.8874	87.6%	0.28	[0.21; 0.36]
Omitting Caballero-Valderrama et al. 2024 (Breast cancer, Anthracycline)	0.8594	0.9270	88.2%	0.27	[0.20; 0.36]
Omitting Wang 2020 et al. (Lymphoma , Anthracycline)	0.8662	0.9307	88.1%	0.27	[0.20; 0.36]
Omitting Bhagat et al. 2023 (Breast cancer, Anthracycline, doxorubicin and cyclophosphamide)	0.8753	0.9356	88.3%	0.27	[0.20; 0.35]
Omitting Park et al., 2020 (Breast cancer, Trastuzumab)	0.8700	0.9327	88.1%	0.27	[0.20; 0.36]
Omitting Guan et al. 2021 (Breast cancer, Anthracycline chemotherapy ± targeted drugs (trastuzumab, pertuzumab))	0.8357	0.9142	87.8%	0.28	[0.20; 0.36]
Omitting Mornos et al. 2013 (Several types of cancer, Anthracycline)	0.8504	0.9222	88.0%	0.27	[0.20; 0.36]
Omitting Mele et al. 2016 (Breast cancer, Trastuzumab, taxane, anthracyclines)	0.8622	0.9285	88.3%	0.27	[0.20; 0.35]
Omitting Anqi et al. 2019 (Breast cancer, Anthracycline)	0.8507	0.9223	88.1%	0.26	[0.19; 0.35]
Omitting Chang et al. 2021 (Lymphoma, Anthracycline)	0.8368	0.9148	87.7%	0.26	[0.19; 0.35]
Omitting Gripp et al. 2018 (Breast cancer,Anthracycline and/or Trastuzumab)	0.8313	0.9117	88.0%	0.28	[0.20; 0.36]
Omitting Cadeddu et al. 2017 (Breast cancer, Epirubicin-based anthracycline + trastuzumab)	0.8511	0.9225	88.1%	0.27	[0.20; 0.36]
Omitting Di Lisi 2023 (Breast Cancer, Anthracycline-containing chemotherapy followed by taxane ± trastuzumab)	0.8788	0.9375	88.3%	0.27	[0.20; 0.35]
Omitting BenKridis et al. 2020 (breast cancer, Anthracycline-containing chemotherapy followed by taxane ± trastuzumab)	0.8541	0.9242	88.1%	0.27	[0.20; 0.36]
Omitting AboElMagdRasheed et al. 2022 (Breast cancer, Anthracycline)	0.8691	0.9323	88.2%	0.27	[0.20; 0.36]
Omitting Stoodley et al. 2013 (Breast cancer, Anthracycline)	0.8262	0.9090	87.8%	0.28	[0.20; 0.36]
Omitting Yang et al. 2024 (Gastrointestinal cancers, Fluorouracil-based chemotherapy)	0.8800	0.9381	88.3%	0.27	[0.20; 0.36]
Omitting Liu et al. 2022 (Breast cancer, anthracycline or trastuzumab)	0.6444	0.8027	85.2%	0.25	[0.19; 0.33]
Omitting Fallah-Rad et al. 2011 (Breast cancer, trastuzumab following anthracycline-based chemotherapy)	0.8783	0.9372	88.3%	0.27	[0.20; 0.36]
Omitting Li et al. 2024 (Lymphoma, Anthracycline)	0.8684	0.9319	88.1%	0.27	[0.20; 0.36]
Omitting Allam et al. 2023 (Several types of cancer, Anthracycline)	0.8438	0.9186	87.9%	0.28	[0.20; 0.36]
Omitting Zhang et al. 2022 (Several types of cancer, Anthracycline)	0.8611	0.9279	87.5%	0.26	[0.19; 0.35]
Omitting Negishi et al. 2013 (Breast cancer, Trastuzumab)	0.8808	0.9385	88.3%	0.27	[0.20; 0.36]
Omitting Kar et al. 2023 (Breast cancer, Anthracycline and/or trastuzumab)	0.7478	0.8648	87.7%	0.26	[0.20; 0.33]
Omitting Huynh etal. 2025 (Breast cancer, (epirubicin and cyclophosphamide), Paclitaxel, Docetaxel Trastuzumab and Pertuzumab)	0.7773	0.8816	87.9%	0.26	[0.19; 0.34]
Omitting Tahir et al. 2022 (Breast cancer, Epirubicin□ or Radiotherapy)	0.8515	0.9227	88.0%	0.27	[0.20; 0.36]
Omitting Cheng et al. 2023 (Breast cancer, trastuzumab and/or pertuzumab)	0.8758	0.9359	88.3%	0.27	[0.20; 0.36]
Omitting Kersten et al., 2023 (Breast cancer, Anti-cancer drugs)	0.7856	0.8863	87.6%	0.26	[0.19; 0.34]
Omitting Lenihan et al. 2025 (Lymphoma, Anthracycline)	0.8067	0.8982	87.4%	0.26	[0.19; 0.34]
**Total (95% CI)**	**0.8406**	**0.9168**	**88.0%**	**0.27**	**[0.20; 0.35]**

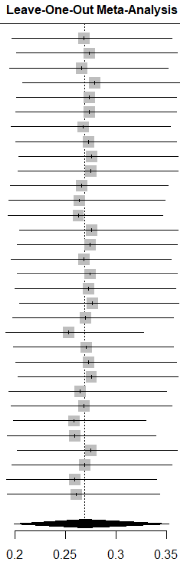

Supplementary Figure S4. Forest plot showing the leave-one-out sensitivity analysis of the pooled incidence of cancer therapy–related cardiac dysfunction (CTRCD) using a random-effects inverse-variance model [7,14,19–49].

Supplementary Figure S5. Leave-one-out sensitivity analysis of Change in GLS from baseline with and without CTRCD

(5A) Change in GLS at visit 1 in patients with and without CTRCD.

Study	P-value	Tau2	Tau	I2	MD	95%-CI
Omitting El□Sherbeny et al. 2019 (CTRCD, Clinical)	0.0008	1.4539	1.2058	80.6%	-2.37	[-3.41; -1.33]
Omitting Di Lisi 2023 (CTRCD, Sub-Clinical)	0.0002	0.6087	0.7802	58.3%	-2.18	[-2.98; -1.38]
Omitting Moya et al. 2023 (Mild CTRCD, Clinical)	0.0005	1.3597	1.1661	80.5%	-2.46	[-3.48; -1.45]
Omitting Charbonnel et al. 2017 (CTRCD, Sub-Clinical)	< 0.0001	0.7449	0.8631	70.3%	-2.73	[-3.52; -1.94]
Omitting Chang et al. 2021 (CTRCD, Subclinical)	0.0007	1.4522	1.2051	80.5%	-2.43	[-3.47; -1.39]
Omitting Gripp et al. 2018 (CTRCD, Sub-Clinical)	0.0006	1.3698	1.1704	80.5%	-2.36	[-3.37; -1.35]
Omitting Fallah-Rad et al. 2011 (CTRCD, Clinical)	0.0007	1.3940	1.1807	80.5%	-2.36	[-3.38; -1.34]
Omitting Allam et al. 2023 (CTRCD, Sub-Clinical)	0.0006	1.3913	1.1795	80.6%	-2.44	[-3.46; -1.42]
Omitting Negishi et al. 2013 (CTRCD, Sub-Clinical)	0.0006	1.4287	1.1953	79.8%	-2.48	[-3.51; -1.44]
Omitting Caballero-Valderrama et al. 2024 (CTRCD, Clinical)	0.0002	1.1144	1.0557	76.4%	-2.62	[-3.56; -1.68]
**Total (95% CI)**	**0.0002**	**1.2395**	**1.1133**	**78.2%**	**-2.44**	**[-3.35; -1.53]**

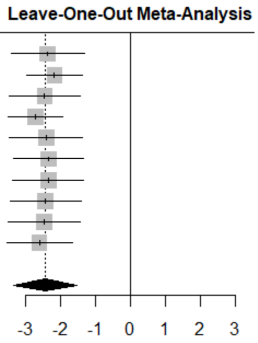

(5B) Change in GLS at visit 2 in patients with and without CTRCD.

Study	P-value	Tau2	Tau	I2	MD	95%-CI
Omitting El□Sherbeny et al. 2019 (CTRCD, Clinical)	0.0018	0.3168	0.5628	39.6%	-3.30	[-4.54; -2.05]
Omitting Moya et al. 2023 (Mild CTRCD, Clinical)	0.0012	0.4438	0.6662	56.9%	-3.77	[-5.04; -2.51]
Omitting Charbonnel et al. 2017 (CTRCD, Sub-Clinical)	0.0002	0.1702	0.4125	31.7%	-3.95	[-4.82; -3.08]
Omitting Chang et al. 2021 (CTRCD, Subclinical)	0.0029	0.8474	0.9206	67.7%	-3.39	[-4.84; -1.94]
Omitting Fallah-Rad et al. 2011 (CTRCD, Clinical)	0.0028	0.8631	0.9290	67.1%	-3.51	[-4.99; -2.02]
Omitting Caballero-Valderrama et al. 2024 (CTRCD, Clinical)	0.0029	0.8745	0.9351	67.5%	-3.48	[-4.96; -1.99]
**Total (95% CI)**	**0.0004**	**0.5720**	**0.7563**	**59.8%**	**-3.58**	**[-4.69; -2.47]**

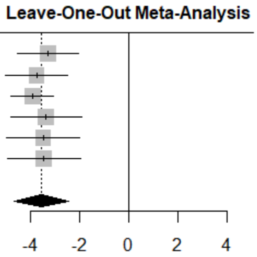

(5C) Change in GLS at visit 3 in patients with and without CTRCD.

Study	P-value	Tau2	Tau	I2	MD	95%-CI
Omitting El□Sherbeny et al. 2019 (CTRCD, Clinical)	0.2137	6.6182	2.5726	93.1%	-5.40	[-29.35; 18.55]
Omitting Charbonnel et al. 2017 (CTRCD, Sub-Clinical)	0.0173	0	0	0%	-6.91	[-9.30; -4.52]
Omitting Fallah-Rad et al. 2011 (CTRCD, Clinical)	0.1941	5.1266	2.2642	94.7%	-5.22	[-26.12; 15.67]
**Total (95% CI)**	**0.0374**	**3.7158**	**1.9276**	**90.6%**	**-5.90**	**[-10.95; -0.85]**

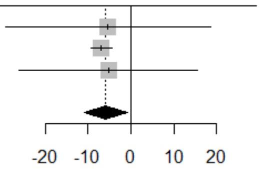

(5D) Change in GLS at the last visit in patients with and without CTRCD.

Study	P-value	Tau2	Tau	I2	MD	95%-CI
Omitting El□Sherbeny et al. 2019 (CTRCD, Clinical)	0.0004	1.5463	1.2435	78.0%	-2.51	[-3.55; -1.47]
Omitting Di Lisi 2023 (CTRCD, Sub-Clinical)	0.0006	1.2922	1.1367	68.4%	-2.28	[-3.29; -1.27]
Omitting Moya et al. 2023 (Mild CTRCD, Clinical)	0.0005	1.3595	1.1660	76.3%	-2.32	[-3.31; -1.33]
Omitting Moya et al. 2023 (Moderate CTRCD, Clinical)	0.0002	1.4147	1.1894	75.8%	-2.46	[-3.41; -1.50]
Omitting Charbonnel et al. 2017 (CTRCD, Sub-Clinical)	0.0003	1.5089	1.2284	76.8%	-2.57	[-3.61; -1.54]
Omitting Chang et al. 2021 (CTRCD, Subclinical)	0.0002	1.3837	1.1763	74.5%	-2.61	[-3.62; -1.60]
Omitting Gripp et al. 2018 (CTRCD, Sub-Clinical)	0.0006	1.6579	1.2876	78.1%	-2.45	[-3.52; -1.37]
Omitting Fallah-Rad et al. 2011 (CTRCD, Clinical)	0.0006	1.4957	1.2230	77.2%	-2.36	[-3.40; -1.33]
Omitting Allam et al. 2023 (CTRCD, Sub-Clinical)	0.0002	0.9379	0.9684	71.4%	-2.21	[-3.05; -1.37]
Omitting Negishi et al. 2013 (CTRCD, Sub-Clinical)	0.0005	1.6215	1.2734	77.8%	-2.52	[-3.59; -1.46]
Omitting Lenihan et al. 2025 (CTRCD, sub clinical)	0.0002	1.3256	1.1513	73.1%	-2.63	[-3.63; -1.63]
Omitting Lenihan et al. 2025 (CTRCD, Clinical)	0.0002	1.4147	1.1894	75.8%	-2.46	[-3.41; -1.50]
Omitting Caballero-Valderrama et al. 2024 (CTRCD, Clinical)	0.0003	1.4937	1.2222	77.2%	-2.57	[-3.59; -1.54]
**Total (95% CI)**	**0.0002**	**1.4147**	**1.1894**	**75.8%**	**-2.46**	**[-3.41; -1.50]**

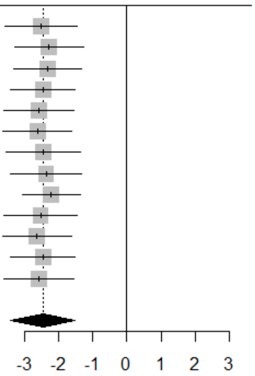

Supplementary Figure S5. Leave-one-out sensitivity analysis of change in GLS from baseline with and without CTRCD. (A) Change in GLS at visit 1 in patients with and without CTRCD. (B) Change in GLS at visit 2 in patients with and without CTRCD. (C) Change in GLS at visit 3 in patients with and without CTRCD. (D) Change in GLS at the last visit in patients with and without CTRCD [7,19,20,24,26,27,29,31,32,37,38,42,43,45,47]

Supplementary Figure S6. Leave-one-out sensitivity analysis of Change in LVEF from baseline with and without CTRCD

(6A) Change in LVEF at visit 1 in patients with and without CTRCD.

Study	P-value	Tau2	Tau	I2	MD	95%-CI
Omitting El□Sherbeny et al. 2019 (CTRCD, Clinical)	0.0030	1.7082	1.3070	44.4%	2.57	[1.16; 3.98]
Omitting Di Lisi 2023 (CTRCD, Sub-Clinical)	0.0010	0.7800	0.8832	20.3%	2.75	[1.50; 4.00]
Omitting Moya et al. 2023 (Mild CTRCD, Clinical)	0.0023	1.4212	1.1921	44.0%	2.52	[1.20; 3.85]
Omitting Charbonnel et al. 2017 (CTRCD, Sub-Clinical)	0.0010	1.1598	1.0769	35.8%	2.67	[1.46; 3.88]
Omitting Chang et al. 2021 (CTRCD, Subclinical)	0.0032	1.1719	1.0825	39.8%	2.30	[1.03; 3.58]
Omitting Gripp et al. 2018 (CTRCD, Sub-Clinical)	0.0025	1.1692	1.0813	42.8%	2.38	[1.11; 3.65]
Omitting Fallah-Rad et al. 2011 (CTRCD, Clinical)	0.0034	1.1583	1.0762	38.7%	2.28	[1.00; 3.56]
Omitting Allam et al. 2023 (CTRCD, Sub-Clinical)	0.0024	0.6584	0.8114	22.2%	2.09	[0.98; 3.20]
Omitting Negishi et al. 2013 (CTRCD, Sub-Clinical)	0.0043	0.8515	0.9228	29.4%	2.14	[0.89; 3.39]
Omitting Caballero-Valderrama et al. 2024 (CTRCD, Clinical)	0.0026	1.4431	1.2013	44.4%	2.50	[1.17; 3.84]
**Total (95% CI)**	**0.0013**	**1.1666**	**1.0801**	**37.5%**	**2.42**	**[1.23; 3.61]**

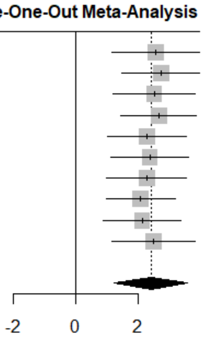

(6B) Change in LVEF at visit 2 in patients with and without CTRCD.

Study	P-value	Tau2	Tau	I2	MD	95%-CI
Omitting El□Sherbeny et al. 2019 (CTRCD, Clinical)	0.1792	84.0831	9.1697	93.5%	6.91	[-4.89; 18.70]
Omitting Moya et al. 2023 (Mild CTRCD, Clinical)	0.0758	85.9573	9.2713	96.4%	10.15	[-1.68; 21.99]
Omitting Charbonnel et al. 2017 (CTRCD, Sub-Clinical)	0.0877	91.8717	9.5850	96.7%	9.89	[-2.32; 22.10]
Omitting Chang et al. 2021 (CTRCD, Subclinical)	0.1077	99.5220	9.9761	97.0%	9.44	[-3.24; 22.12]
Omitting Fallah-Rad et al. 2011 (CTRCD, Clinical)	0.1201	40.2287	6.3426	96.7%	5.68	[-2.32; 13.69]
Omitting Caballero-Valderrama et al. 2024 (CTRCD, Clinical)	0.1071	99.0929	9.9545	97.1%	9.43	[-3.21; 22.08]
**Total (95% CI)**	**0.0750**	**82.7730**	**9.0980**	**96.6%**	**8.56**	**[-1.25; 18.38]**

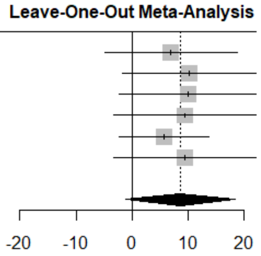

(6C) Change in LVEF at visit 3 in patients with and without CTRCD.

Study	P-value	Tau2	Tau	I2	MD	95%-CI
Omitting El□Sherbeny et al. 2019 (CTRCD, Clinical)	0.2868	163.3258	12.7799	97.2%	18.95	[-97.49; 135.39]
Omitting Charbonnel et al. 2017 (CTRCD, Sub-Clinical)	0.0874	21.3728	4.6231	93.3%	24.47	[-18.50; 67.43]
Omitting Fallah-Rad et al. 2011 (CTRCD, Clinical)	0.2241	63.1608	7.9474	94.5%	15.72	[-57.64; 89.08]
**Total (95% CI)**	**0.0646**	**79.2097**	**8.9000**	**94.6%**	**19.81**	**[-2.97; 42.60]**

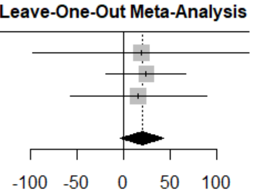

(6D) Change in LVEF at the last visit in patients with and without CTRCD.

Study	P-value	Tau2	Tau	I2	MD	95%-CI
Omitting El□Sherbeny et al. 2019 (CTRCD, Clinical)	0.0015	20.6757	4.5470	93.3%	6.97	[3.46; 10.48]
Omitting Di Lisi 2023 (CTRCD, Sub-Clinical)	0.0002	15.5528	3.9437	87.4%	8.55	[5.39; 11.71]
Omitting Moya et al. 2023 (Mild CTRCD, Clinical)	0.0006	22.5710	4.7509	96.5%	8.07	[4.51; 11.63]
Omitting Charbonnel et al. 2017 (CTRCD, Sub-Clinical)	0.0014	20.8961	4.5712	96.2%	7.00	[3.50; 10.49]
Omitting Chang et al. 2021 (CTRCD, Subclinical)	0.0009	24.1096	4.9102	96.5%	7.96	[4.25; 11.67]
Omitting Gripp et al. 2018 (CTRCD, Sub-Clinical)	0.0015	23.8438	4.8830	96.5%	7.33	[3.64; 11.01]
Omitting Fallah-Rad et al. 2011 (CTRCD, Clinical)	0.0013	21.3692	4.6227	96.4%	7.06	[3.59; 10.54]
Omitting Allam et al. 2023 (CTRCD, Sub-Clinical)	0.0016	24.2573	4.9252	96.5%	7.34	[3.61; 11.06]
Omitting Negishi et al. 2013 (CTRCD, Sub-Clinical)	0.0013	25.4505	5.0449	96.5%	7.69	[3.89; 11.50]
Omitting Lenihan et al. 2025 (CTRCD, sub clinical)	0.0010	24.5236	4.9521	96.5%	7.89	[4.15; 11.63]
Omitting Caballero-Valderrama et al. 2024 (CTRCD, Clinical)	0.0008	23.8089	4.8794	96.5%	7.98	[4.30; 11.67]
**Total (95% CI)**	**0.0005**	**22.5382**	**4.7474**	**96.2%**	**7.62**	**[4.24; 11.00]**

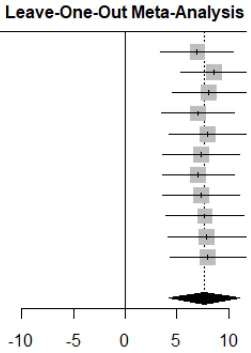

Supplementary Figure S6. Leave-one-out sensitivity analysis of change in LVEF from baseline with and without CTRCD. (A) Change in LVEF at visit 1 in patients with and without CTRCD. (B) Change in LVEF at visit 2 in patients with and without CTRCD. (C) Change in LVEF at visit 3 in patients with and without CTRCD. (D) Change in LVEF at the last visit in patients with and without CTRCD [7,20,24,26,27,29,31,32,37,42,43].

Supplementary Figure S7. Leave-one-out sensitivity analysis of the change in GLS from baseline in CTRCD.

(7A) Change in GLS at visit 1 in CTRCD patients.

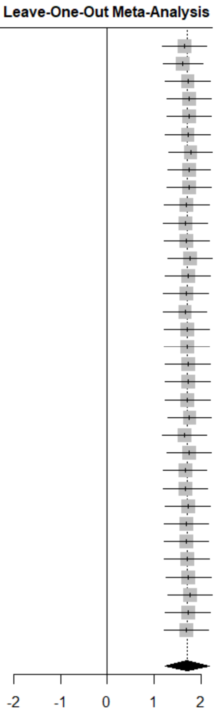

Study	P-value	Tau2	Tau	I2	MD	95%-CI
Omitting El□Sherbeny et al. 2019 (CTRCD, Clinical)	< 0.0001	1.2811	1.1318	84.7%	1.65	[1.18; 2.12]
Omitting Di Lisi 2023 (CTRCD, Sub-Clinical)	< 0.0001	0.9803	0.9901	83.2%	1.61	[1.18; 2.03]
Omitting Moya et al. 2023 (Mild CTRCD, Clinical)	< 0.0001	1.3462	1.1603	86.1%	1.71	[1.24; 2.19]
Omitting Moya et al. 2023 (Moderate CTRCD, Clinical)	< 0.0001	1.2925	1.1369	85.9%	1.74	[1.28; 2.21]
Omitting Charbonnel et al. 2017 (CTRCD, Sub-Clinical)	< 0.0001	1.3028	1.1414	85.9%	1.74	[1.27; 2.21]
Omitting Caballero-Valderrama et al. 2024 (CTRCD, Clinical)	< 0.0001	1.3607	1.1665	86.1%	1.71	[1.23; 2.19]
Omitting Hazim et al. 2023 (CTRCD, Subclinical) (with anthracycline) (Mixture)	< 0.0001	1.2081	1.0991	85.2%	1.77	[1.31; 2.23]
Omitting Hazim et al. 2023 (CTRCD, Subclinical) (without anthracycline) (Mixture)	< 0.0001	1.2831	1.1327	85.9%	1.74	[1.29; 2.20]
Omitting Bhagat et al. 2023 (CTRCD, Sub-Clinical) (Mixture)	< 0.0001	1.3013	1.1408	85.8%	1.75	[1.28; 2.22]
Omitting Park et al., 2020 (CTRCD, Clinical) (Mixture)	< 0.0001	1.3630	1.1675	86.1%	1.69	[1.21; 2.17]
Omitting Guan et al. 2021 (CTRCD, Sub-Clinical) (Mixture)	< 0.0001	1.3276	1.1522	85.5%	1.66	[1.19; 2.14]
Omitting Mornos et al. 2013 (CTRCD, Clinical)(Mixture)	< 0.0001	1.3707	1.1708	86.0%	1.69	[1.21; 2.17]
Omitting Mele et al. 2016 (CTRCD, Sub-Clinical)(Mixture)	< 0.0001	1.2569	1.1211	85.4%	1.76	[1.30; 2.23]
Omitting Anqi et al. 2019 (CTRCD, Sub-Clinical)(Mixture)	< 0.0001	1.3669	1.1691	86.0%	1.72	[1.24; 2.20]
Omitting Chang et al. 2021 (CTRCD, Subclinical)	< 0.0001	1.3478	1.1610	86.0%	1.68	[1.20; 2.15]
Omitting Gripp et al. 2018 (CTRCD, Sub-Clinical)	< 0.0001	1.2693	1.1267	85.8%	1.66	[1.19; 2.12]
Omitting Cadeddu et al. 2017 (CTRCD, Clinical)(Mixture)	< 0.0001	1.3825	1.1758	86.1%	1.70	[1.21; 2.18]
Omitting Di Lisi 2023 (CTRCD, Sub-Clinical)(Mixture)	< 0.0001	1.3817	1.1755	86.1%	1.70	[1.21; 2.18]
Omitting BenKridis et al. 2020 (CTRCD, Clinical)(Mixture)	< 0.0001	1.3709	1.1709	86.0%	1.72	[1.24; 2.20]
Omitting AboElMagdRasheed et al. 2022 (CTRCD, Clinical)(Mixture)	< 0.0001	1.3738	1.1721	86.0%	1.71	[1.23; 2.20]
Omitting Stoodley et al., 2013 (CTRCD, Sub-Clinical)(Mixture)	< 0.0001	1.3772	1.1735	86.1%	1.71	[1.23; 2.19]
Omitting Yang et al. 2024 (CTRCD, Sub-Clinical)(Mixture)	< 0.0001	1.2944	1.1377	85.5%	1.75	[1.28; 2.22]
Omitting Liu et al.2022 (CTRCD, Sub-Clinical) (anthracycline without trastuzumab)(Mixture)	< 0.0001	1.2507	1.1183	82.8%	1.64	[1.18; 2.11]
Omitting Liu et al.2022 (CTRCD, Sub-Clinical) (trastuzumab without anthracycline)(Mixture)	< 0.0001	1.3091	1.1441	83.6%	1.75	[1.27; 2.22]
Omitting Fallah-Rad et al. 2011 (CTRCD, Clinical)	< 0.0001	1.2863	1.1341	85.7%	1.66	[1.19; 2.12]
Omitting Allam et al. 2023 (CTRCD, Sub-Clinical)	< 0.0001	1.2914	1.1364	85.9%	1.66	[1.20; 2.13]
Omitting Zhang et al. 2022 (CTRCD, Clinical)(Mixture)	< 0.0001	1.3751	1.1727	85.8%	1.72	[1.24; 2.20]
Omitting Negishi et al. 2013 (CTRCD, Sub-Clinical)	< 0.0001	1.3568	1.1648	86.0%	1.68	[1.20; 2.16]
Omitting Kar et al. 2023 (CTRCD, Sub-Clinical)(Mixture)	< 0.0001	1.3573	1.1650	86.0%	1.68	[1.20; 2.16]
Omitting Huynh et al. 2025 (CTRCD, Sub-Clinical)(Mixture)	< 0.0001	1.3759	1.1730	86.1%	1.70	[1.22; 2.18]
Omitting Tahir et al. 2022 (CTRCD, Sub-Clinical) (Epirubicin□based (CMR))(Mixture)	< 0.0001	1.3556	1.1643	85.9%	1.73	[1.25; 2.21]
Omitting Tahir et al. 2022 (CTRCD, Sub-Clinical) (Radiotherapy (CMR)(Mixture)	< 0.0001	1.2622	1.1235	85.4%	1.76	[1.29; 2.23]
Omitting Kersten et al. 2023 (CTRCD, Clinical)(Mixture)	< 0.0001	1.3694	1.1702	86.0%	1.72	[1.24; 2.20]
Omitting Li et al. 2024 (CTRCD, Sub-Clinical)(Mixure)	< 0.0001	1.3578	1.1652	86.1%	1.69	[1.21; 2.16]
**Total (95% CI)**	**< 0.0001**	**1.3171**	**1.1477**	**85.7%**	**1.70**	**[1.24; 2.17]**

(7B) Change in GLS at visit 2 in CTRCD patients.

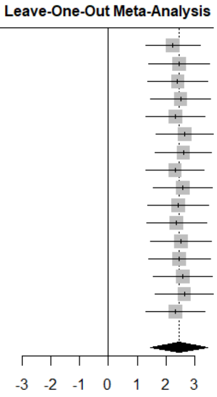

Study	P-value	Tau2	Tau	I2	MD	95%-CI
Omitting El□Sherbeny et al. 2019 (CTRCD, Clinical)	0.0002	2.3517	1.5335	87.3%	2.24	[1.28; 3.19]
Omitting Moya et al. 2023 (Mild CTRCD, Clinical)	0.0002	3.1182	1.7658	92.4%	2.44	[1.38; 3.50]
Omitting Moya et al. 2023 (Moderate CTRCD, Clinical)	0.0002	3.0403	1.7437	92.4%	2.40	[1.35; 3.44]
Omitting Charbonnel et al. 2017 (CTRCD, Sub-Clinical)	0.0002	3.0378	1.7429	92.4%	2.50	[1.45; 3.54]
Omitting Caballero-Valderrama et al. 2024 (CTRCD, Clinical)	0.0002	2.8369	1.6843	92.2%	2.32	[1.31; 3.33]
Omitting Hazim et al. 2023 (CTRCD, Subclinical) (with anthracycline) (Mixture)	< 0.0001	2.6397	1.6247	91.6%	2.63	[1.64; 3.62]
Omitting Hazim et al. 2023 (CTRCD, Subclinical) (without anthracycline) (Mixture)	< 0.0001	2.7333	1.6533	92.2%	2.59	[1.62; 3.56]
Omitting Guan et al. 2021 (CTRCD, Sub-Clinical) (Mixture)	0.0002	2.8020	1.6739	91.2%	2.31	[1.29; 3.32]
Omitting Mele et al. 2016 (CTRCD, Sub-Clinical)(Mixture)	0.0001	2.9158	1.7076	92.1%	2.57	[1.54; 3.60]
Omitting Anqi et al. 2019 (CTRCD, Sub-Clinical)(Mixture)	0.0002	3.1188	1.7660	92.4%	2.41	[1.35; 3.47]
Omitting Chang et al. 2021 (CTRCD, Subclinical)	0.0003	3.0035	1.7331	92.2%	2.36	[1.31; 3.40]
Omitting Cadeddu et al. 2017 (CTRCD, Clinical)(Mixture)	0.0002	3.1067	1.7626	91.8%	2.51	[1.45; 3.57]
Omitting BenKridis et al. 2020 (CTRCD, Clinical)(Mixture)	0.0002	3.1523	1.7755	92.4%	2.46	[1.40; 3.53]
Omitting Stoodley et al., 2013 (CTRCD, Sub-Clinical)(Mixture)	< 0.0001	2.8899	1.7000	91.5%	2.58	[1.55; 3.61]
Omitting Yang et al. 2024 (CTRCD, Sub-Clinical)(Mixture)	< 0.0001	2.6747	1.6355	91.2%	2.63	[1.63; 3.63]
Omitting Fallah-Rad et al. 2011 (CTRCD, Clinical)	0.0002	2.8767	1.6961	92.2%	2.33	[1.31; 3.35]
**Total (95% CI)**	**< 0.0001**	**2.8952**	**1.7015**	**91.9%**	**2.46**	**[1.47; 3.44]**

(7C) Change in GLS at visit 3 in CTRCD patients

Study	P-value	Tau2	Tau	I2	MD	95%-CI
Omitting El□Sherbeny et al. 2019 (CTRCD, Clinical)	0.0229	7.8673	2.8049	96.1%	3.01	[0.56; 5.46]
Omitting Charbonnel et al. 2017 (CTRCD, Sub-Clinical)	0.0208	10.9138	3.3036	97.7%	3.57	[0.73; 6.42]
Omitting Hazim et al. 2023 (CTRCD, Subclinical) (with anthracycline) (Mixture)	0.0099	9.3199	3.0529	97.5%	3.94	[1.28; 6.59]
Omitting Hazim et al. 2023 (CTRCD, Subclinical) (without anthracycline) (Mixture)	0.0071	8.6927	2.9483	97.6%	3.99	[1.48; 6.50]
Omitting Guan et al. 2021 (CTRCD, Sub-Clinical) (Mixture)	0.0264	10.2601	3.2031	97.5%	3.28	[0.51; 6.04]
Omitting Cadeddu et al. 2017 (CTRCD, Clinical)(Mixture)	0.0160	10.5441	3.2472	96.9%	3.75	[0.94; 6.56]
Omitting BenKridis et al. 2020 (CTRCD, Clinical)(Mixture)	0.0262	10.4348	3.2303	97.4%	3.31	[0.52; 6.09]
Omitting Yang et al. 2024 (CTRCD, Sub-Clinical)(Mixture)	0.0078	8.6658	2.9438	97.1%	4.02	[1.44; 6.60]
Omitting Fallah-Rad et al. 2011 (CTRCD, Clinical)	0.0238	8.7038	2.9502	97.5%	3.09	[0.55; 5.63]
**Total (95% CI)**	**0.0101**	**9.4815**	**3.0792**	**97.3%**	**3.55**	**[1.10; 6.00]**

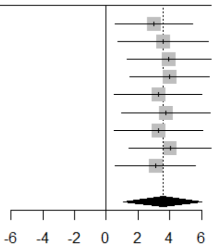

(7D) Change in GLS at the last visit in CTRCD patients

Study	P-value	Tau2	Tau	I2	MD	95%-CI
Omitting El□Sherbeny et al. 2019 (CTRCD, Clinical)	< 0.0001	2.5696	1.6030	91.0%	2.68	[2.09; 3.27]
Omitting Di Lisi 2023 (CTRCD, Sub-Clinical)	< 0.0001	2.5514	1.5973	91.1%	2.67	[2.09; 3.26]
Omitting Moya et al. 2023 (Mild CTRCD, Clinical)	< 0.0001	2.6059	1.6143	91.3%	2.69	[2.10; 3.28]
Omitting Moya et al. 2023 (Moderate CTRCD, Clinical)	< 0.0001	2.6236	1.6198	91.3%	2.73	[2.13; 3.32]
Omitting Charbonnel et al. 2017 (CTRCD, Sub-Clinical)	< 0.0001	2.6447	1.6263	91.3%	2.74	[2.14; 3.34]
Omitting Caballero-Valderrama et al. 2024 (CTRCD, Clinical)	< 0.0001	2.6339	1.6229	91.3%	2.74	[2.15; 3.34]
Omitting Hazim et al. 2023 (CTRCD, Subclinical) (with anthracycline) (Mixture)	< 0.0001	2.6130	1.6165	91.1%	2.76	[2.17; 3.36]
Omitting Hazim et al. 2023 (CTRCD, Subclinical) (without anthracycline) (Mixture)	< 0.0001	2.4981	1.5805	91.2%	2.78	[2.21; 3.36]
Omitting Bhagat et al. 2023 (CTRCD, Sub-Clinical) (Mixture)	< 0.0001	2.5492	1.5966	91.1%	2.78	[2.19; 3.36]
Omitting Park et al., 2020 (CTRCD, Clinical) (Mixture)	< 0.0001	2.4257	1.5574	91.0%	2.65	[2.08; 3.22]
Omitting Guan et al. 2021 (CTRCD, Sub-Clinical) (Mixture)	< 0.0001	2.1431	1.4639	89.8%	2.61	[2.06; 3.16]
Omitting Mornos et al. 2013 (CTRCD, Clinical)(Mixture)	< 0.0001	2.6094	1.6154	91.2%	2.69	[2.10; 3.28]
Omitting Mele et al. 2016 (CTRCD, Sub-Clinical)(Mixture)	< 0.0001	2.5077	1.5836	90.9%	2.79	[2.21; 3.37]
Omitting Anqi et al. 2019 (CTRCD, Sub-Clinical)(Mixture)	< 0.0001	2.5553	1.5985	91.1%	2.67	[2.09; 3.26]
Omitting Chang et al. 2021 (CTRCD, Subclinical)	< 0.0001	2.6391	1.6245	91.2%	2.75	[2.16; 3.35]
Omitting Gripp et al. 2018 (CTRCD, Sub-Clinical)	< 0.0001	2.6296	1.6216	91.3%	2.70	[2.11; 3.29]
Omitting Cadeddu et al. 2017 (CTRCD, Clinical)(Mixture)	< 0.0001	2.6610	1.6313	91.3%	2.71	[2.11; 3.31]
Omitting Di Lisi 2023 (CTRCD, Sub-Clinical)(Mixture)	< 0.0001	2.6626	1.6317	91.3%	2.71	[2.12; 3.31]
Omitting BenKridis et al. 2020 (CTRCD, Clinical)(Mixture)	< 0.0001	2.5795	1.6061	91.0%	2.68	[2.09; 3.27]
Omitting AboElMagdRasheed et al. 2022 (CTRCD, Clinical)(Mixture)	< 0.0001	2.6302	1.6218	91.2%	2.76	[2.16; 3.35]
Omitting Stoodley et al., 2013 (CTRCD, Sub-Clinical)(Mixture)	< 0.0001	2.3752	1.5412	89.5%	2.81	[2.24; 3.39]
Omitting Yang et al. 2024 (CTRCD, Sub-Clinical)(Mixture)	< 0.0001	2.6280	1.6211	91.2%	2.76	[2.16; 3.35]
Omitting Liu et al.2022 (CTRCD, Sub-Clinical) (anthracycline without trastuzumab)(Mixture)	< 0.0001	2.6005	1.6126	90.2%	2.68	[2.09; 3.28]
Omitting Liu et al.2022 (CTRCD, Sub-Clinical) (trastuzumab without anthracycline)(Mixture)	< 0.0001	2.6450	1.6264	91.2%	2.70	[2.11; 3.30]
Omitting Fallah-Rad et al. 2011 (CTRCD, Clinical)	< 0.0001	2.6163	1.6175	91.3%	2.70	[2.11; 3.29]
Omitting Allam et al. 2023 (CTRCD, Sub-Clinical)	< 0.0001	2.3804	1.5428	91.0%	2.64	[2.08; 3.21]
Omitting Zhang et al. 2022 (CTRCD, Clinical)(Mixture)	< 0.0001	2.6541	1.6291	91.2%	2.74	[2.15; 3.34]
Omitting Negishi et al. 2013 (CTRCD, Sub-Clinical)	< 0.0001	2.6514	1.6283	91.3%	2.74	[2.14; 3.33]
Omitting Kar et al. 2023 (CTRCD, Sub-Clinical)(Mixture)	< 0.0001	2.6576	1.6302	91.3%	2.73	[2.13; 3.33]
Omitting Huynh et al. 2025 (CTRCD, Sub-Clinical)(Mixture)	< 0.0001	2.6345	1.6231	91.2%	2.75	[2.16; 3.35]
Omitting Tahir et al. 2022 (CTRCD, Sub-Clinical) (Epirubicin□based (CMR))(Mixture)	< 0.0001	2.5621	1.6006	90.9%	2.78	[2.19; 3.37]
Omitting Tahir et al. 2022 (CTRCD, Sub-Clinical) (Radiotherapy (CMR)(Mixture)	< 0.0001	2.4040	1.5505	90.1%	2.81	[2.24; 3.38]
Omitting Cheng et al. 2023 (CTRCD, Clinical)(Mixture)	< 0.0001	2.5648	1.6015	91.0%	2.78	[2.19; 3.37]
Omitting Kersten et al. 2023 (CTRCD, Clinical)(Mixture)	< 0.0001	2.5774	1.6054	91.1%	2.77	[2.18; 3.36]
Omitting Lenihan et al. 2025 (CTRCD, Clinical) Sub-CTX	< 0.0001	2.6463	1.6267	91.2%	2.75	[2.15; 3.34]
Omitting Lenihan et al. 2025 (CTRCD, Clinical) CTX	< 0.0001	2.6402	1.6249	91.3%	2.75	[2.15; 3.34]
Omitting Li et al. 2024 (CTRCD, Sub-Clinical)(Mixure)	< 0.0001	2.6151	1.6171	91.2%	2.69	[2.10; 3.29]
**Total (95% CI)**	**< 0.0001**	**2.5725**	**1.6039**	**91.1%**	**2.73**	**[2.15; 3.31]**

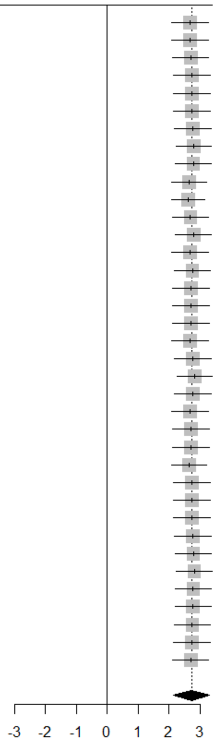

Supplementary Figure S7. Leave-one-out sensitivity analysis of change in GLS from baseline in CTRCD. (A) Change in GLS at visit 1 in CTRCD patients. (B) Change in GLS at visit 2 in CTRCD patients. (C) Change in GLS at visit 3 in CTRCD patients. (D) Change in GLS at the last visit in CTRCD patients [7,14,19–46,48,49].

Supplementary Figure S8. Leave-one-out sensitivity analysis of change in LVEF from baseline in CTRCD.

(8A) Change in LVEF at visit 1 in CTRCD patients.

Study	P-value	Tau2	Tau	I2	MD	95%-CI
Omitting El□Sherbeny et al. 2019 (CTRCD, Clinical)	< 0.0001	1.9302	1.3893	64.6%	-1.05	[-1.39; -0.72]
Omitting Di Lisi 2023 (CTRCD, Sub-Clinical)	< 0.0001	2.1358	1.4614	66.9%	-1.15	[-1.48; -0.82]
Omitting Moya et al. 2023 (Mild CTRCD, Clinical)	< 0.0001	2.0670	1.4377	66.9%	-1.14	[-1.46; -0.81]
Omitting Moya et al. 2023 (Moderate CTRCD, Clinical)	< 0.0001	2.0194	1.4210	66.9%	-1.14	[-1.47; -0.82]
Omitting Charbonnel et al. 2017 (CTRCD, Sub-Clinical)	< 0.0001	2.0495	1.4316	66.7%	-1.15	[-1.48; -0.82]
Omitting Caballero-Valderrama et al. 2024 (CTRCD, Clinical)	< 0.0001	2.0536	1.4330	66.7%	-1.13	[-1.46; -0.80]
Omitting Hazim et al. 2023 (CTRCD, Subclinical) (with anthracycline) (Mixture)	< 0.0001	1.9821	1.4079	65.8%	-1.10	[-1.43; -0.77]
Omitting Hazim et al. 2023 (CTRCD, Subclinical) (without anthracycline) (Mixture)	< 0.0001	1.8995	1.3782	65.4%	-1.20	[-1.53; -0.87]
Omitting Bhagat et al. 2023 (CTRCD, Sub-Clinical) (Mixture)	< 0.0001	2.0752	1.4406	66.4%	-1.11	[-1.44; -0.78]
Omitting Park et al., 2020 (CTRCD, Clinical) (Mixture)	< 0.0001	2.0464	1.4305	66.9%	-1.14	[-1.47; -0.82]
Omitting Guan et al. 2021 (CTRCD, Sub-Clinical) (Mixture)	< 0.0001	2.1483	1.4657	66.9%	-1.14	[-1.48; -0.81]
Omitting Mornos et al. 2013 (CTRCD, Clinical)(Mixture)	< 0.0001	2.1136	1.4538	66.8%	-1.15	[-1.49; -0.82]
Omitting Mele et al. 2016 (CTRCD, Sub-Clinical)(Mixture)	< 0.0001	2.0469	1.4307	66.5%	-1.17	[-1.50; -0.84]
Omitting Anqi et al. 2019 (CTRCD, Sub-Clinical)(Mixture)	< 0.0001	2.0822	1.4430	66.7%	-1.13	[-1.46; -0.80]
Omitting Chang et al. 2021 (CTRCD, Subclinical)	< 0.0001	2.0602	1.4353	66.9%	-1.14	[-1.47; -0.81]
Omitting Gripp et al. 2018 (CTRCD, Sub-Clinical)	< 0.0001	1.9852	1.4090	66.6%	-1.14	[-1.46; -0.81]
Omitting Cadeddu et al. 2017 (CTRCD, Clinical)(Mixture)	< 0.0001	1.8788	1.3707	60.8%	-1.41	[-1.76; -1.05]
Omitting Di Lisi 2023 (CTRCD, Sub-Clinical)(Mixture)	< 0.0001	2.0420	1.4290	65.6%	-1.23	[-1.58; -0.89]
Omitting BenKridis et al. 2020 (CTRCD, Clinical)(Mixture)	< 0.0001	2.1411	1.4633	66.7%	-1.11	[-1.44; -0.78]
Omitting AboElMagdRasheed et al. 2022 (CTRCD, Clinical)(Mixture)	< 0.0001	2.1227	1.4570	66.8%	-1.13	[-1.46; -0.80]
Omitting Stoodley et al., 2013 (CTRCD, Sub-Clinical)(Mixture)	< 0.0001	2.1607	1.4699	66.9%	-1.15	[-1.50; -0.81]
Omitting Yang et al. 2024 (CTRCD, Sub-Clinical)(Mixture)	< 0.0001	2.1150	1.4543	66.9%	-1.14	[-1.47; -0.81]
Omitting Liu et al.2022 (CTRCD, Sub-Clinical) (anthracycline without trastuzumab)(Mixture)	< 0.0001	1.9566	1.3988	65.9%	-1.19	[-1.52; -0.86]
Omitting Liu et al.2022 (CTRCD, Sub-Clinical) (trastuzumab without anthracycline)(Mixture)	< 0.0001	2.0518	1.4324	66.3%	-1.19	[-1.52; -0.85]
Omitting Fallah-Rad et al. 2011 (CTRCD, Clinical)	< 0.0001	1.6275	1.2757	63.3%	-1.09	[-1.41; -0.76]
Omitting Allam et al. 2023 (CTRCD, Sub-Clinical)	< 0.0001	1.5044	1.2265	61.5%	-1.09	[-1.41; -0.76]
Omitting Zhang et al. 2022 (CTRCD, Clinical)(Mixture)	< 0.0001	1.8023	1.3425	60.4%	-0.94	[-1.29; -0.60]
Omitting Negishi et al. 2013 (CTRCD, Sub-Clinical)	< 0.0001	1.5539	1.2466	62.6%	-1.08	[-1.41; -0.75]
Omitting Kar et al. 2023 (CTRCD, Sub-Clinical)(Mixture)	< 0.0001	2.0698	1.4387	66.9%	-1.15	[-1.47; -0.82]
Omitting Huynh et al. 2025 (CTRCD, Sub-Clinical)(Mixture)	< 0.0001	2.0442	1.4298	66.8%	-1.14	[-1.47; -0.82]
Omitting Tahir et al. 2022 (CTRCD, Sub-Clinical) (Epirubicin□based (CMR))(Mixture)	< 0.0001	2.0495	1.4316	66.6%	-1.16	[-1.49; -0.83]
Omitting Tahir et al. 2022 (CTRCD, Sub-Clinical) (Radiotherapy (CMR)(Mixture)	< 0.0001	1.8429	1.3575	65.3%	-1.18	[-1.51; -0.85]
Omitting Kersten et al. 2023 (CTRCD, Clinical)(Mixture)	< 0.0001	1.9303	1.3894	65.8%	-1.11	[-1.44; -0.78]
Omitting Li et al. 2024 (CTRCD, Sub-Clinical)(Mixure)	< 0.0001	2.0951	1.4475	66.9%	-1.13	[-1.46; -0.81]
**Total (95% CI)**	**< 0.0001**	**1.9858**	**1.4092**	**65.9%**	**-1.14**	**[-1.47; -0.81]**

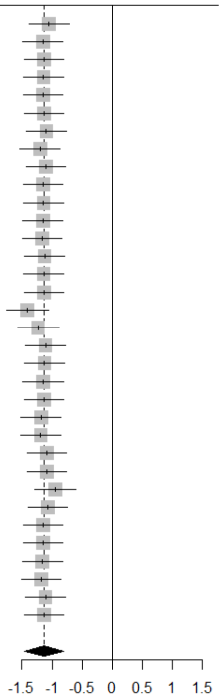

(8B) Change in LVEF at visit 2 in CTRCD patients.

Study	P-value	Tau2	Tau	I2	MD	95%-CI
Omitting El□Sherbeny et al. 2019 (CTRCD, Clinical)	0.0067	15.9704	3.9963	83.3%	-3.92	[-6.56; -1.28]
Omitting Moya et al. 2023 (Mild CTRCD, Clinical)	0.0054	31.7446	5.6342	95.1%	-5.11	[-8.44; -1.77]
Omitting Moya et al. 2023 (Moderate CTRCD, Clinical)	0.0087	30.9799	5.5660	94.9%	-4.70	[-8.00; -1.39]
Omitting Charbonnel et al. 2017 (CTRCD, Sub-Clinical)	0.0057	31.7956	5.6388	95.1%	-5.08	[-8.41; -1.74]
Omitting Caballero-Valderrama et al. 2024 (CTRCD, Clinical)	0.0064	31.8948	5.6475	95.1%	-4.99	[-8.33; -1.65]
Omitting Hazim et al. 2023 (CTRCD, Subclinical) (with anthracycline) (Mixture)	0.0060	32.1601	5.6710	95.1%	-5.06	[-8.41; -1.70]
Omitting Hazim et al. 2023 (CTRCD, Subclinical) (without anthracycline) (Mixture)	0.0042	30.7732	5.5474	94.9%	-5.24	[-8.53; -1.94]
Omitting Guan et al. 2021 (CTRCD, Sub-Clinical) (Mixture)	0.0067	32.4214	5.6940	95.0%	-4.98	[-8.35; -1.62]
Omitting Mele et al. 2016 (CTRCD, Sub-Clinical)(Mixture)	0.0045	31.0836	5.5753	95.0%	-5.21	[-8.51; -1.90]
Omitting Anqi et al. 2019 (CTRCD, Sub-Clinical)(Mixture)	0.0081	32.0328	5.6598	94.9%	-4.82	[-8.18; -1.47]
Omitting Chang et al. 2021 (CTRCD, Subclinical)	0.0050	31.4379	5.6069	95.0%	-5.16	[-8.47; -1.84]
Omitting Cadeddu et al. 2017 (CTRCD, Clinical)(Mixture)	0.0052	31.7177	5.6318	94.8%	-5.15	[-8.48; -1.81]
Omitting BenKridis et al. 2020 (CTRCD, Clinical)(Mixture)	0.0060	32.1764	5.6724	95.1%	-5.07	[-8.42; -1.71]
Omitting Stoodley et al., 2013 (CTRCD, Sub-Clinical)(Mixture)	0.0045	31.0720	5.5742	94.7%	-5.22	[-8.52; -1.91]
Omitting Yang et al. 2024 (CTRCD, Sub-Clinical)(Mixture)	0.0051	31.6191	5.6231	95.0%	-5.14	[-8.47; -1.81]
Omitting Fallah-Rad et al. 2011 (CTRCD, Clinical)	0.0030	17.1500	4.1413	94.2%	-3.98	[-6.36; -1.60]
**Total (95% CI)**	**0.0042**	**29.7186**	**5.4515**	**94.7%**	**-4.93**	**[-8.04; -1.81]**

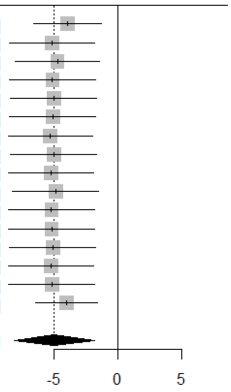

(8C) Change in LVEF at visit 3 in CTRCD patients.

Study	P-value	Tau2	Tau	I2	MD	95%-CI
Omitting El□Sherbeny et al. 2019 (CTRCD, Clinical)	0.0415	58.2294	7.6308	95.9%	-6.89	[-13.43; -0.35]
Omitting Charbonnel et al. 2017 (CTRCD, Sub-Clinical)	0.0404	88.9902	9.4335	98.9%	-8.45	[-16.42; -0.49]
Omitting Hazim et al. 2023 (CTRCD, Subclinical) (with anthracycline) (Mixture)	0.0306	88.9303	9.4303	98.9%	-9.10	[-17.06; -1.13]
Omitting Hazim et al. 2023 (CTRCD, Subclinical) (without anthracycline) (Mixture)	0.0196	80.8151	8.9897	98.8%	-9.68	[-17.28; -2.09]
Omitting Guan et al. 2021 (CTRCD, Sub-Clinical) (Mixture)	0.0316	89.5058	9.4607	98.9%	-9.05	[-17.04; -1.06]
Omitting Cadeddu et al. 2017 (CTRCD, Clinical)(Mixture)	0.0234	84.4722	9.1909	98.7%	-9.49	[-17.26; -1.72]
Omitting BenKridis et al. 2020 (CTRCD, Clinical)(Mixture)	0.0240	85.0259	9.2209	98.9%	-9.45	[-17.24; -1.66]
Omitting Yang et al. 2024 (CTRCD, Sub-Clinical)(Mixture)	0.0247	85.4750	9.2453	98.9%	-9.41	[-17.22; -1.61]
Omitting Fallah-Rad et al. 2011 (CTRCD, Clinical)	0.0330	49.5708	7.0407	98.7%	-6.66	[-12.61; -0.72]
**Total (95% CI)**	**0.0199**	**79.0164**	**8.8891**	**98.7%**	**-8.69**	**[-15.60; -1.78]**

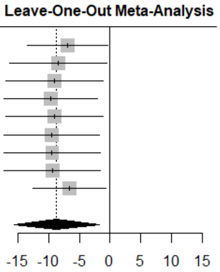

(8D) Change in LVEF at the last visit in CTRCD patients.

Study	P-value	Tau2	Tau	I2	MD	95%-CI
Omitting El□Sherbeny et al. 2019 (CTRCD, Clinical)	< 0.0001	21.3983	4.6258	93.1%	-4.81	[-6.46; -3.16]
Omitting Di Lisi 2023 (CTRCD, Sub-Clinical)	< 0.0001	23.8972	4.8885	95.2%	-5.24	[-6.96; -3.51]
Omitting Moya et al. 2023 (Mild CTRCD, Clinical)	< 0.0001	24.4207	4.9417	95.3%	-5.14	[-6.88; -3.40]
Omitting Moya et al. 2023 (Moderate CTRCD, Clinical)	< 0.0001	24.2229	4.9217	95.3%	-5.09	[-6.82; -3.35]
Omitting Charbonnel et al. 2017 (CTRCD, Sub-Clinical)	< 0.0001	20.9759	4.5799	94.8%	-4.79	[-6.41; -3.16]
Omitting Caballero-Valderrama et al. 2024 (CTRCD, Clinical)	< 0.0001	24.2578	4.9252	95.3%	-5.10	[-6.83; -3.36]
Omitting Hazim et al. 2023 (CTRCD, Subclinical) (with anthracycline) (Mixture)	< 0.0001	24.3685	4.9364	95.3%	-5.17	[-6.91; -3.43]
Omitting Hazim et al. 2023 (CTRCD, Subclinical) (without anthracycline) (Mixture)	< 0.0001	23.9769	4.8966	95.2%	-5.23	[-6.95; -3.50]
Omitting Bhagat et al. 2023 (CTRCD, Sub-Clinical) (Mixture)	< 0.0001	24.3588	4.9355	95.3%	-5.17	[-6.91; -3.43]
Omitting Park et al., 2020 (CTRCD, Clinical) (Mixture)	< 0.0001	19.5211	4.4183	94.7%	-4.72	[-6.29; -3.15]
Omitting Guan et al. 2021 (CTRCD, Sub-Clinical) (Mixture)	< 0.0001	24.3427	4.9338	95.2%	-5.05	[-6.78; -3.31]
Omitting Mornos et al. 2013 (CTRCD, Clinical)(Mixture)	< 0.0001	24.3705	4.9367	95.3%	-5.17	[-6.91; -3.43]
Omitting Mele et al. 2016 (CTRCD, Sub-Clinical)(Mixture)	< 0.0001	23.7033	4.8686	95.2%	-5.25	[-6.97; -3.54]
Omitting Anqi et al. 2019 (CTRCD, Sub-Clinical)(Mixture)	< 0.0001	23.7687	4.8753	95.2%	-4.97	[-6.69; -3.25]
Omitting Chang et al. 2021 (CTRCD, Subclinical)	< 0.0001	24.2810	4.9276	95.3%	-5.18	[-6.92; -3.44]
Omitting Gripp et al. 2018 (CTRCD, Sub-Clinical)	< 0.0001	23.4527	4.8428	95.3%	-4.96	[-6.66; -3.26]
Omitting Cadeddu et al. 2017 (CTRCD, Clinical)(Mixture)	< 0.0001	24.5201	4.9518	95.3%	-5.12	[-6.86; -3.37]
Omitting Di Lisi 2023 (CTRCD, Sub-Clinical)(Mixture)	< 0.0001	23.8275	4.8813	95.0%	-5.24	[-6.97; -3.52]
Omitting BenKridis et al. 2020 (CTRCD, Clinical)(Mixture)	< 0.0001	24.4371	4.9434	95.3%	-5.16	[-6.90; -3.42]
Omitting AboElMagdRasheed et al. 2022 (CTRCD, Clinical)(Mixture)	< 0.0001	24.2183	4.9212	95.3%	-5.20	[-6.93; -3.46]
Omitting Stoodley et al., 2013 (CTRCD, Sub-Clinical)(Mixture)	< 0.0001	23.6673	4.8649	95.0%	-5.26	[-6.97; -3.54]
Omitting Yang et al. 2024 (CTRCD, Sub-Clinical)(Mixture)	< 0.0001	24.3330	4.9329	95.3%	-5.17	[-6.91; -3.44]
Omitting Liu et al.2022 (CTRCD, Sub-Clinical) (anthracycline without trastuzumab)(Mixture)	< 0.0001	23.8370	4.8823	95.2%	-5.24	[-6.96; -3.52]
Omitting Liu et al.2022 (CTRCD, Sub-Clinical) (trastuzumab without anthracycline)(Mixture)	< 0.0001	23.9925	4.8982	95.2%	-5.23	[-6.95; -3.50]
Omitting Fallah-Rad et al. 2011 (CTRCD, Clinical)	< 0.0001	21.6104	4.6487	95.0%	-4.82	[-6.47; -3.18]
Omitting Allam et al. 2023 (CTRCD, Sub-Clinical)	< 0.0001	22.4439	4.7375	95.1%	-4.87	[-6.54; -3.20]
Omitting Zhang et al. 2022 (CTRCD, Clinical)(Mixture)	< 0.0001	24.4955	4.9493	95.3%	-5.13	[-6.87; -3.39]
Omitting Negishi et al. 2013 (CTRCD, Sub-Clinical)	< 0.0001	23.9947	4.8984	95.2%	-5.00	[-6.72; -3.27]
Omitting Kar et al. 2023 (CTRCD, Sub-Clinical)(Mixture)	< 0.0001	23.8003	4.8786	95.3%	-5.24	[-6.96; -3.52]
Omitting Huynh et al. 2025 (CTRCD, Sub-Clinical)(Mixture)	< 0.0001	24.1105	4.9102	95.3%	-5.18	[-6.91; -3.45]
Omitting Tahir et al. 2022 (CTRCD, Sub-Clinical) (Epirubicin□based (CMR))(Mixture)	< 0.0001	23.6859	4.8668	95.2%	-5.26	[-6.97; -3.54]
Omitting Tahir et al. 2022 (CTRCD, Sub-Clinical) (Radiotherapy (CMR)(Mixture)	< 0.0001	23.7260	4.8709	95.3%	-5.25	[-6.97; -3.53]
Omitting Cheng et al. 2023 (CTRCD, Clinical)(Mixture)	< 0.0001	24.4692	4.9466	95.3%	-5.09	[-6.83; -3.35]
Omitting Kersten et al. 2023 (CTRCD, Clinical)(Mixture)	< 0.0001	24.4307	4.9427	95.3%	-5.14	[-6.88; -3.39]
Omitting Lenihan et al. 2025 (CTRCD, Clinical) Sub-CTX	< 0.0001	24.4531	4.9450	95.3%	-5.11	[-6.85; -3.37]
Omitting Lenihan et al. 2025 (CTRCD, Clinical) CTX	< 0.0001	24.0176	4.9008	95.3%	-5.02	[-6.74; -3.29]
Omitting Li et al. 2024 (CTRCD, Sub-Clinical)(Mixure)	< 0.0001	24.4857	4.9483	95.3%	-5.13	[-6.88; -3.39]
**Total (95% CI)**	**< 0.0001**	**23.7280**	**4.8711**	**95.2%**	**-5.10**	**[-6.80; -3.41]**

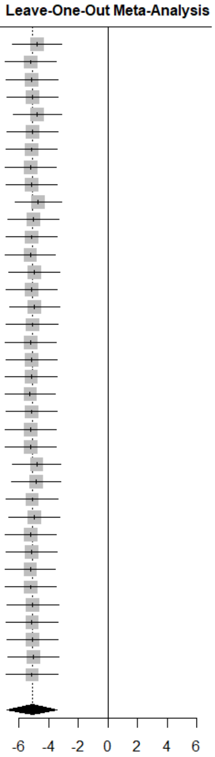

Supplementary Figure S8. Leave-one-out sensitivity analysis of change in LVEF from baseline in CTRCD. (A) Change in LVEF at visit 1 in CTRCD patients. (B) Change in LVEF at visit 2 in CTRCD patients. (C) Change in LVEF at visit 3 in CTRCD patients. (D) Change in LVEF at the last visit in CTRCD patients [7,14,19–46,48,49].

Supplementary Figure S9. Subgroup and Sensitivity Analyses of GLS and LVEF Changes Stratified by CTRCD Definition

Supplementary Figure S9. Subgroup and sensitivity analyses of GLS and LVEF changes stratified by CTRCD definition. The figure includes analyses of changes in GLS and LVEF at visit 1, visit 2, visit 3, and the last visit in patients with and without CTRCD, as well as subgroup analyses in CTRCD and non-CTRCD cohorts and CMR-derived measures where applicable [7,14,19–49].

Change in GLS at visit 1 in patients with and without CTRCD.

Study or Subgroup	CTRCD Mean	CTRCD SD	CTRCD Total	Non-CTRCD Mean	Non-CTRCD SD	Non-CTRCD Total	Weight	Mean Difference IV, Random, 95% CI
**CTRCD_Definition = GLS-only**								
Chang et al. 2021 (CTRCD, Subclinical)	-2.60	2.46	36	-0.10	2.42	38	11.0%	-2.50 [-3.61; -1.39]
**CTRCD_Definition = Guideline-based composite**								
Moya et al. 2023 (Mild CTRCD, Clinical)	-1.20	3.12	10	0.90	2.55	16	6.6%	-2.10 [-4.40; 0.20]
Gripp et al. 2018 (CTRCD, Sub-Clinical)	-4.10	1.74	5	-0.90	2.05	44	8.8%	-3.20 [-4.84; -1.56]
Allam et al. 2023 (CTRCD, Sub-Clinical)	-3.70	2.92	10	-1.30	3.15	70	7.7%	-2.40 [-4.35; -0.45]
**Total (95% CI)**			**25**			**130**	**23.1%**	**-2.69 [-4.13; -1.25]**
Heterogeneity: $Tau^2 = 0$; $Chi^2 = 0.71$, df = 2 (P = 0.7026); $I^2 = 0\%$								
**CTRCD_Definition = Imaging plus biomarkers**								
Di Lisi 2023 (CTRCD, Sub-Clinical)	-4.90	1.90	28	-0.40	2.23	141	12.2%	-4.50 [-5.29; -3.71]
**CTRCD_Definition = LVEF-only**								
El□Sherbeny et al. 2019 (CTRCD, Clinical)	-3.18	0.94	18	-0.32	1.40	43	12.9%	-2.86 [-3.46; -2.26]
Charbonnel et al. 2017 (CTRCD, Sub-Clinical)	0.00	1.71	6	0.00	2.56	80	9.5%	0.00 [-1.48; 1.48]
Fallah-Rad et al. 2011 (CTRCD, Clinical)	-3.40	1.57	10	-0.30	2.35	32	10.4%	-3.10 [-4.37; -1.83]
Negishi et al. 2013 (CTRCD, Sub-Clinical)	-2.40	2.39	24	-0.30	2.00	57	11.1%	-2.10 [-3.19; -1.01]
Caballero-Valderrama et al. 2024 (CTRCD, Clinical)	-1.34	1.31	5	-0.43	2.14	28	9.8%	-0.91 [-2.30; 0.48]
**Total (95% CI)**			**63**			**240**	**53.7%**	**-1.89 [-3.48; -0.29]**
Heterogeneity: $Tau^2 = 1.2367$; $Chi^2 = 18.07$, df = 4 (P = 0.0012); $I^2 = 77.9\%$								
**Total (95% CI)**			**152**			**549**	**100.0%**	**-2.44 [-3.35; -1.53]**
**Prediction interval**								**[-5.13; 0.24]**
Heterogeneity: $Tau^2 = 1.2395$; $Chi^2 = 41.31$, df = 9 (P < 0.0001); $I^2 = 78.2\%$								
Test for overall effect: $t_9 = -6.07$ (P = 0.0002)								
Test for subgroup differences: $Chi^2 = 18.97$, df = 3 (P = 0.0003)								

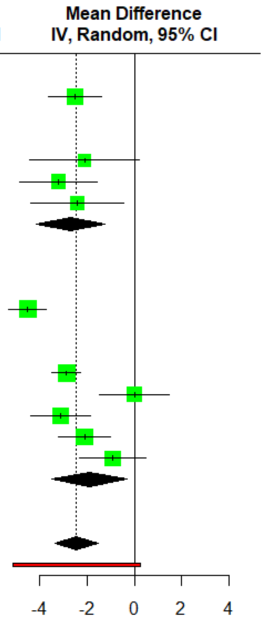

Change in GLS at visit 2 in patients with and without CTRCD.

Study or Subgroup	CTRCD Mean	CTRCD SD	CTRCD Total	Non-CTRCD Mean	Non-CTRCD SD	Non-CTRCD Total	Weight	Mean Difference IV, Random, 95% CI
**CTRCD_Definition = GLS-only**								
Chang et al. 2021 (CTRCD, Subclinical)	-3.80	2.42	36	0.30	2.49	38	19.2%	-4.10 [-5.22; -2.98]
**CTRCD_Definition = Guideline-based composite**								
Moya et al. 2023 (Mild CTRCD, Clinical)	-2.60	2.04	10	-0.10	2.29	16	13.1%	-2.50 [-4.19; -0.81]
**CTRCD_Definition = LVEF-only**								
El□Sherbeny et al. 2019 (CTRCD, Clinical)	-5.33	1.05	18	-0.81	1.36	43	25.5%	-4.52 [-5.15; -3.89]
Charbonnel et al. 2017 (CTRCD, Sub-Clinical)	-1.56	2.41	6	-0.13	2.37	80	10.7%	-1.43 [-3.43; 0.57]
Fallah-Rad et al. 2011 (CTRCD, Clinical)	-4.40	1.80	10	-0.80	2.62	32	15.5%	-3.60 [-5.04; -2.16]
Caballero-Valderrama et al. 2024 (CTRCD, Clinical)	-4.62	1.43	5	-0.88	1.68	28	15.9%	-3.74 [-5.14; -2.34]
**Total (95% CI)**			**39**			**183**	**67.7%**	**-3.57 [-5.51; -1.62]**
Heterogeneity: $Tau^2 = 0.8897$; $Chi^2 = 9.28$, df = 3 (P = 0.0258); $I^2 = 67.7\%$								
**Total (95% CI)**			**85**			**237**	**100.0%**	**-3.58 [-4.69; -2.47]**
**Prediction interval**								**[-5.80; -1.36]**
Heterogeneity: $Tau^2 = 0.5720$; $Chi^2 = 12.44$, df = 5 (P = 0.0293); $I^2 = 59.8\%$								
Test for overall effect: $t_5 = -8.30$ (P = 0.0004)								
Test for subgroup differences: $Chi^2 = 2.39$, df = 2 (P = 0.3022)								

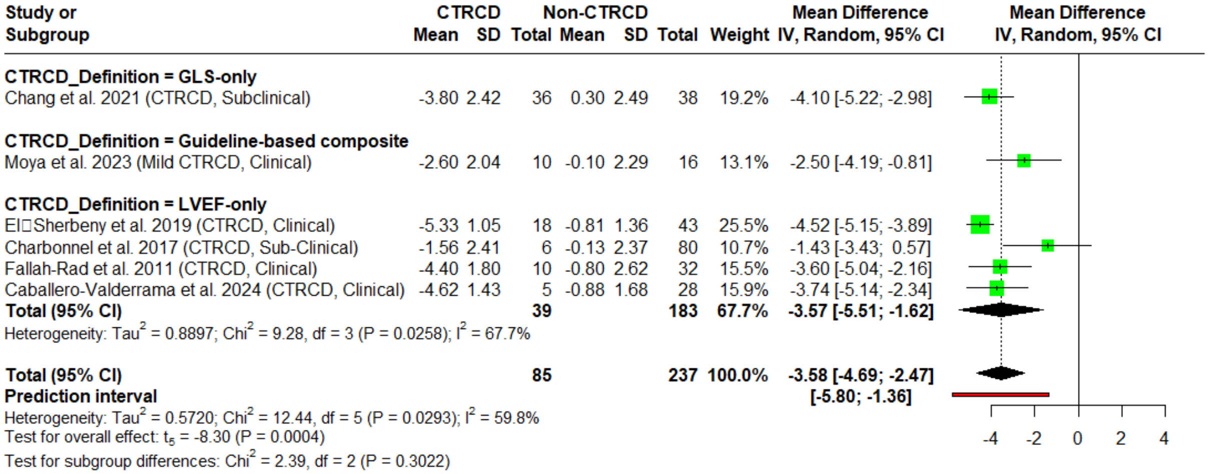

Change in GLS at visit 3 in patients with and without CTRCD.

Study or Subgroup	CTRCD Mean	CTRCD SD	CTRCD Total	Non-CTRCD Mean	Non-CTRCD SD	Non-CTRCD Total	Weight	Mean Difference IV, Random, 95% CI
**CTRCD_Definition = LVEF-only**								
El□Sherbeny et al. 2019 (CTRCD, Clinical)	-7.78	1.25	18	-0.96	1.28	43	35.4%	-6.82 [-7.51; -6.13]
Charbonnel et al. 2017 (CTRCD, Sub-Clinical)	-3.30	1.49	6	0.23	2.46	80	32.6%	-3.53 [-4.84; -2.22]
Fallah-Rad et al. 2011 (CTRCD, Clinical)	-7.40	1.97	10	-0.10	2.14	32	32.0%	-7.30 [-8.73; -5.87]
**Total (95% CI)**			**34**			**155**	**100.0%**	**-5.90 [-10.95; -0.85]**
Heterogeneity: Tau^2 = 3.7158; Chi^2 = 21.24, df = 2 (P < 0.0001); I^2 = 90.6%								
**Total (95% CI)**			**34**			**155**	**100.0%**	**-5.90 [-10.95; -0.85]**
**Prediction interval**								**[-15.59; 3.79]**

Heterogeneity: Tau^2 = 3.7158; Chi^2 = 21.24, df = 2 (P < 0.0001); I^2 = 90.6%
Test for overall effect: t_2 = -5.02 (P = 0.0374)
Test for subgroup differences: Chi^2 = 0.00, df = 0 (P = NA)

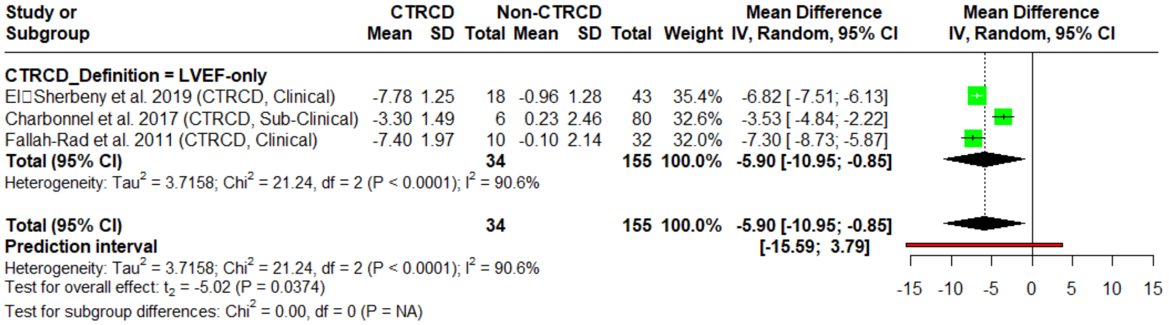

Change in GLS at the last visit in patients with and without CTRCD.

Study or Subgroup	CTRCD Mean	CTRCD SD	CTRCD Total	Non-CTRCD Mean	Non-CTRCD SD	Non-CTRCD Total	Weight	Mean Difference IV, Random, 95% CI
**CTRCD_Definition = GLS-only**								
Chang et al. 2021 (CTRCD, Subclinical)	-1.90	2.46	36	-0.80	2.36	38	10.4%	-1.10 [-2.20; 0.00]
**CTRCD_Definition = Guideline-based composite**								
Moya et al. 2023 (Mild CTRCD, Clinical)	-4.10	2.19	10	0.00	2.72	16	7.6%	-4.10 [-6.00; -2.20]
Moya et al. 2023 (Moderate CTRCD, Clinical)	-2.70	3.84	9	.	.	15	0.0%	
Gripp et al. 2018 (CTRCD, Sub-Clinical)	-3.70	1.15	5	-1.10	2.00	44	10.1%	-2.60 [-3.77; -1.43]
Allam et al. 2023 (CTRCD, Sub-Clinical)	-6.40	2.79	10	-1.10	3.02	70	7.7%	-5.30 [-7.17; -3.43]
**Total (95% CI)**			**34**			**145**	**25.4%**	**-3.86 [-7.30; -0.42]**
Heterogeneity: Tau^2 = 1.3437; Chi^2 = 6.22, df = 2 (P = 0.0446); I^2 = 67.8%								
**CTRCD_Definition = Imaging plus biomarkers**								
Di Lisi 2023 (CTRCD, Sub-Clinical)	-4.60	2.15	28	-0.80	2.17	141	11.1%	-3.80 [-4.67; -2.93]
Lenihan et al. 2025 (CTRCD, sub clinical)	-2.00	1.73	24	-1.00	1.73	19	10.6%	-1.00 [-2.04; 0.04]
Lenihan et al. 2025 (CTRCD, Clinical)	-2.00	1.73	9	.	.	7	0.0%	
**Total (95% CI)**			**61**			**167**	**21.7%**	**-2.42 [-20.20; 15.37]**
Heterogeneity: Tau^2 = 3.6792; Chi^2 = 16.28, df = 1 (P < 0.0001); I^2 = 93.9%								
**CTRCD_Definition = LVEF-only**								
El□Sherbeny et al. 2019 (CTRCD, Clinical)	-4.39	1.35	18	-2.67	7.80	43	6.1%	-1.72 [-4.13; 0.69]
Charbonnel et al. 2017 (CTRCD, Sub-Clinical)	-2.20	1.49	6	-0.80	2.24	80	9.7%	-1.40 [-2.69; -0.11]
Fallah-Rad et al. 2011 (CTRCD, Clinical)	-3.90	2.38	10	-0.40	2.19	32	8.4%	-3.50 [-5.16; -1.84]
Negishi et al. 2013 (CTRCD, Sub-Clinical)	-2.40	2.60	24	-0.50	2.23	57	10.0%	-1.90 [-3.09; -0.71]
Caballero-Valderrama et al. 2024 (CTRCD, Clinical)	-2.08	1.71	5	-0.80	2.05	28	8.3%	-1.28 [-2.96; 0.40]
**Total (95% CI)**			**63**			**240**	**42.6%**	**-1.92 [-2.97; -0.87]**
Heterogeneity: Tau^2 = 0.0775; Chi^2 = 4.69, df = 4 (P = 0.3208); I^2 = 14.7%								
**Total (95% CI)**			**194**			**590**	**100.0%**	**-2.46 [-3.41; -1.50]**
**Prediction interval**								**[-5.27; 0.36]**

Heterogeneity: Tau^2 = 1.4147; Chi^2 = 41.26, df = 10 (P < 0.0001); I^2 = 75.8%
Test for overall effect: t_{10} = -5.75 (P = 0.0002)
Test for subgroup differences: Chi^2 = 8.11, df = 3 (P = 0.0437)

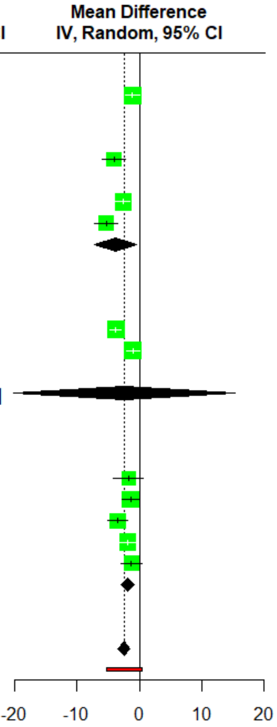

Change in LVEF at visit 1 in patients with and without CTRCD.

Study or Subgroup	CTRCD Mean	CTRCD SD	CTRCD Total	Non-CTRCD Mean	Non-CTRCD SD	Non-CTRCD Total	Weight	Mean Difference IV, Random, 95% CI
**CTRCD_Definition = GLS-only**								
Chang et al. 2021 (CTRCD, Subclinical)	0.90	8.85	36	-3.20	7.62	38	6.4%	4.10 [0.33; 7.87]
**CTRCD_Definition = Guideline-based composite**								
Moya et al. 2023 (Mild CTRCD, Clinical)	1.60	4.06	10	0.10	4.94	16	7.2%	1.50 [-1.99; 4.99]
Gripp et al. 2018 (CTRCD, Sub-Clinical)	6.40	10.74	5	1.10	7.14	44	1.2%	5.30 [-4.35; 14.95]
Allam et al. 2023 (CTRCD, Sub-Clinical)	8.20	4.49	10	3.00	5.71	70	8.6%	5.20 [2.11; 8.29]
**Total (95% CI)**			**25**			**130**	**17.1%**	**3.64 [-1.95; 9.23]**
Heterogeneity: $Tau^2 = 2.6063$; $Chi^2 = 2.54$, df = 2 (P = 0.2813); $I^2 = 21.2\%$								
**CTRCD_Definition = Imaging plus biomarkers**								
Di Lisi 2023 (CTRCD, Sub-Clinical)	0.90	3.50	28	0.00	2.10	141	19.2%	0.90 [-0.44; 2.24]
**CTRCD_Definition = LVEF-only**								
El□Sherbeny et al. 2019 (CTRCD, Clinical)	3.17	2.52	18	1.17	2.92	43	18.2%	2.00 [0.54; 3.46]
Charbonnel et al. 2017 (CTRCD, Sub-Clinical)	0.03	2.86	6	-0.34	4.94	80	11.1%	0.37 [-2.16; 2.90]
Fallah-Rad et al. 2011 (CTRCD, Clinical)	6.00	3.61	10	2.00	7.00	32	7.8%	4.00 [0.70; 7.30]
Negishi et al. 2013 (CTRCD, Sub-Clinical)	6.00	5.11	24	2.00	3.65	57	12.6%	4.00 [1.75; 6.25]
Caballero-Valderrama et al. 2024 (CTRCD, Clinical)	2.30	3.32	5	0.50	4.65	28	7.6%	1.80 [-1.58; 5.18]
**Total (95% CI)**			**63**			**240**	**57.3%**	**2.35 [0.53; 4.18]**
Heterogeneity: $Tau^2 = 0.6280$; $Chi^2 = 5.69$, df = 4 (P = 0.2238); $I^2 = 29.7\%$								
**Total (95% CI)**			**152**			**549**	**100.0%**	**2.42 [1.23; 3.61]**
**Prediction interval**								**[-0.33; 5.17]**
Heterogeneity: $Tau^2 = 1.1666$; $Chi^2 = 14.41$, df = 9 (P = 0.1084); $I^2 = 37.5\%$								
Test for overall effect: $t_9 = 4.60$ (P = 0.0013)								
Test for subgroup differences: $Chi^2 = 5.65$, df = 3 (P = 0.1299)								

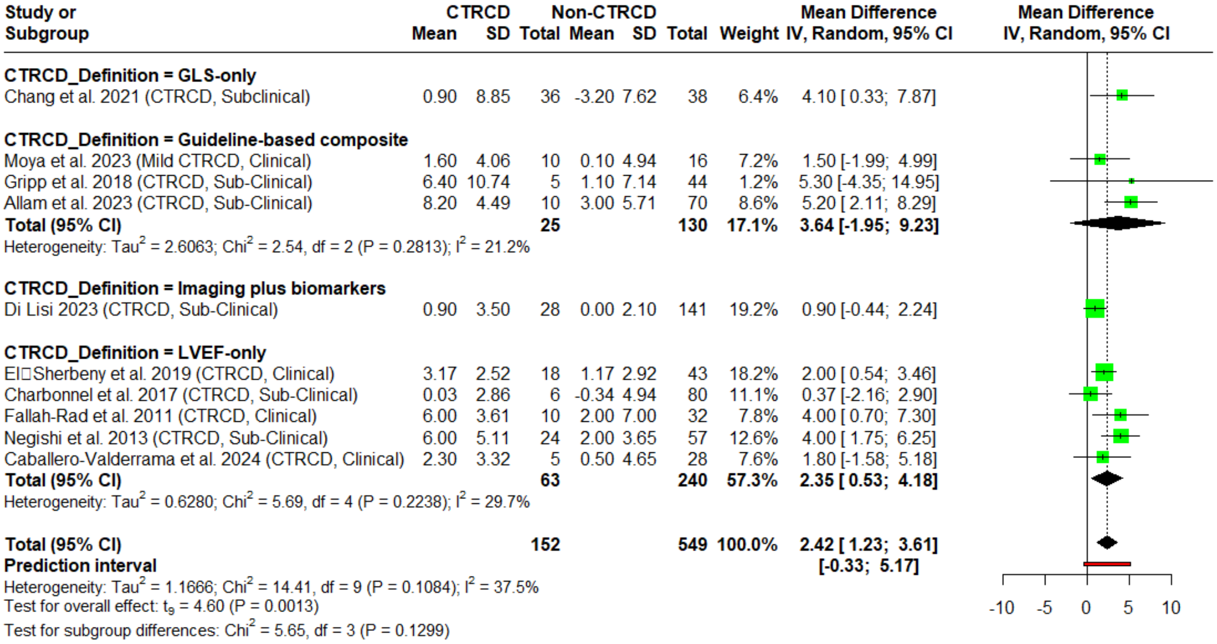

Change in LVEF at visit 2 in patients with and without CTRCD.

Study or Subgroup	CTRCD Mean	CTRCD SD	CTRCD Total	Non-CTRCD Mean	Non-CTRCD SD	Non-CTRCD Total	Weight	Mean Difference IV, Random, 95% CI
**CTRCD_Definition = GLS-only**								
Chang et al. 2021 (CTRCD, Subclinical)	1.60	7.43	36	-2.70	7.11	38	16.8%	4.30 [0.98; 7.62]
**CTRCD_Definition = Guideline-based composite**								
Moya et al. 2023 (Mild CTRCD, Clinical)	2.30	4.12	10	1.60	4.46	16	16.8%	0.70 [-2.66; 4.06]
**CTRCD_Definition = LVEF-only**								
El□Sherbeny et al. 2019 (CTRCD, Clinical)	17.06	3.24	18	0.52	3.03	43	17.2%	16.54 [14.79; 18.29]
Charbonnel et al. 2017 (CTRCD, Sub-Clinical)	2.67	4.15	6	0.66	4.94	80	16.8%	2.01 [-1.48; 5.50]
Fallah-Rad et al. 2011 (CTRCD, Clinical)	22.00	7.94	10	-2.00	4.58	32	16.0%	24.00 [18.83; 29.17]
Caballero-Valderrama et al. 2024 (CTRCD, Clinical)	4.00	4.64	5	-0.20	5.00	28	16.4%	4.20 [-0.27; 8.67]
**Total (95% CI)**			**39**			**183**	**66.4%**	**11.64 [-4.83; 28.12]**
Heterogeneity: $Tau^2 = 102.1491$; $Chi^2 = 85.67$, df = 3 (P < 0.0001); $I^2 = 96.5\%$								
**Total (95% CI)**			**85**			**237**	**100.0%**	**8.56 [-1.25; 18.38]**
**Prediction interval**								**[-16.77; 33.90]**
Heterogeneity: $Tau^2 = 82.7730$; $Chi^2 = 148.38$, df = 5 (P < 0.0001); $I^2 = 96.6\%$								
Test for overall effect: $t_5 = 2.24$ (P = 0.0750)								
Test for subgroup differences: $Chi^2 = 5.18$, df = 2 (P = 0.0752)								

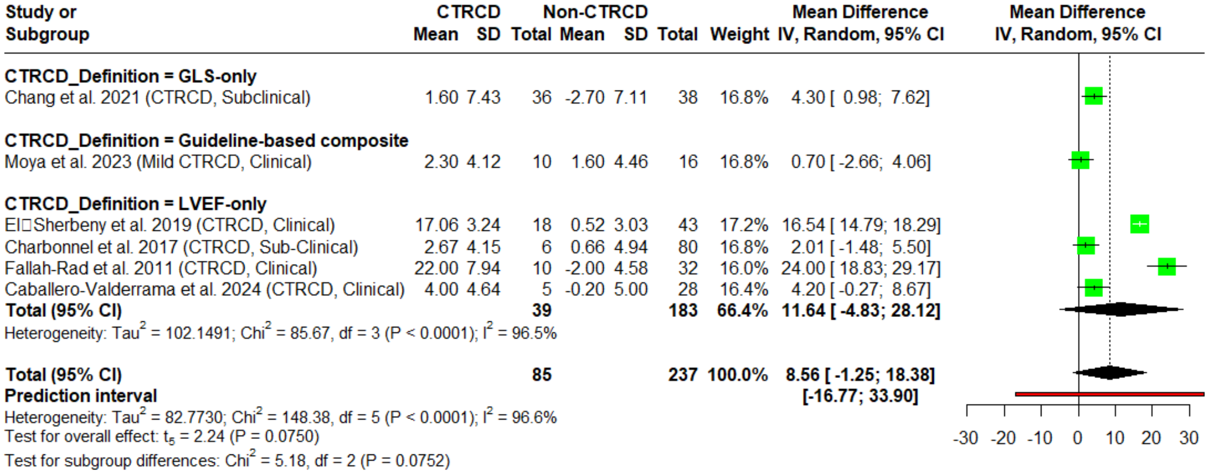

Change in LVEF at visit 3 in patients with and without CTRCD.

Study or Subgroup	CTRCD Mean	SD	Total	Non-CTRCD Mean	SD	Total	Weight	Mean Difference IV, Random, 95% CI
**CTRCD_Definition = LVEF-only**								
El□Sherbeny et al. 2019 (CTRCD, Clinical)	22.47	2.52	18	1.24	2.76	43	34.5%	21.23 [19.80; 22.66]
Charbonnel et al. 2017 (CTRCD, Sub-Clinical)	10.83	6.23	6	1.16	4.94	80	31.9%	9.67 [4.57; 14.77]
Fallah-Rad et al. 2011 (CTRCD, Clinical)	25.00	4.36	10	-3.00	4.58	32	33.6%	28.00 [24.87; 31.13]
**Total (95% CI)**			**34**			**155**	**100.0%**	**19.81 [-2.97; 42.60]**
Heterogeneity: Tau^2 = 79.2097; Chi^2 = 37.27, df = 2 (P < 0.0001); I^2 = 94.6%								
**Total (95% CI)**			**34**			**155**	**100.0%**	**19.81 [-2.97; 42.60]**
**Prediction interval**								**[-24.63; 64.25]**

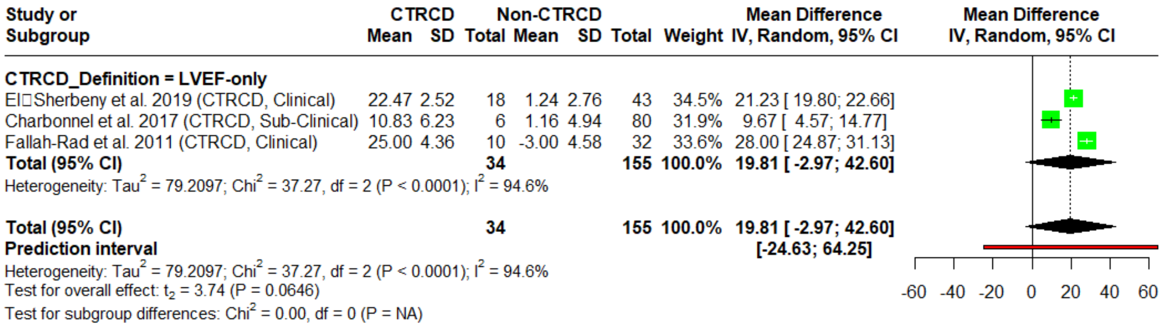

Heterogeneity: Tau^2 = 79.2097; Chi^2 = 37.27, df = 2 (P < 0.0001); I^2 = 94.6%
Test for overall effect: t_2 = 3.74 (P = 0.0646)
Test for subgroup differences: Chi^2 = 0.00, df = 0 (P = NA)

Change in LVEF at the last visit in patients with and without CTRCD.

Study or Subgroup	CTRCD Mean	SD	Total	Non-CTRCD Mean	SD	Total	Weight	Mean Difference IV, Random, 95% CI
**CTRCD_Definition = GLS-only**								
Chang et al. 2021 (CTRCD, Subclinical)	2.50	6.39	36	-1.90	6.94	38	9.5%	4.40 [1.36; 7.44]
**CTRCD_Definition = Guideline-based composite**								
Moya et al. 2023 (Mild CTRCD, Clinical)	3.80	8.87	10	1.70	3.73	16	7.6%	2.10 [-3.69; 7.89]
Gripp et al. 2018 (CTRCD, Sub-Clinical)	12.00	5.91	5	0.90	6.67	44	7.7%	11.10 [5.56; 16.64]
Allam et al. 2023 (CTRCD, Sub-Clinical)	13.50	5.80	10	3.00	5.09	70	9.0%	10.50 [6.71; 14.29]
**Total (95% CI)**			**25**			**130**	**24.3%**	**8.13 [-4.04; 20.30]**
Heterogeneity: Tau^2 = 16.3225; Chi^2 = 6.58, df = 2 (P = 0.0373); I^2 = 69.6%								
**CTRCD_Definition = Imaging plus biomarkers**								
Di Lisi 2023 (CTRCD, Sub-Clinical)	0.70	2.77	28	1.00	2.62	141	10.3%	-0.30 [-1.41; 0.81]
Lenihan et al. 2025 (CTRCD, sub clinical)	5.00	6.24	24	0.00	5.00	19	9.3%	5.00 [1.64; 8.36]
**Total (95% CI)**			**52**			**160**	**19.6%**	**2.10 [-31.42; 35.63]**
Heterogeneity: Tau^2 = 12.4130; Chi^2 = 8.61, df = 1 (P = 0.0034); I^2 = 88.4%								
**CTRCD_Definition = LVEF-only**								
El□Sherbeny et al. 2019 (CTRCD, Clinical)	14.58	2.21	18	1.35	3.59	43	10.2%	13.23 [11.75; 14.71]
Charbonnel et al. 2017 (CTRCD, Sub-Clinical)	15.83	3.41	6	2.33	6.18	80	9.5%	13.50 [10.46; 16.54]
Fallah-Rad et al. 2011 (CTRCD, Clinical)	15.00	7.94	10	1.00	4.44	32	8.0%	14.00 [8.85; 19.15]
Negishi et al. 2013 (CTRCD, Sub-Clinical)	9.00	4.16	24	2.00	5.23	57	9.9%	7.00 [4.85; 9.15]
Caballero-Valderrama et al. 2024 (CTRCD, Clinical)	5.60	3.70	5	1.60	5.10	28	9.0%	4.00 [0.25; 7.75]
**Total (95% CI)**			**63**			**240**	**46.7%**	**10.30 [4.71; 15.89]**
Heterogeneity: Tau^2 = 17.2414; Chi^2 = 39.29, df = 4 (P < 0.0001); I^2 = 89.8%								
**Total (95% CI)**			**176**			**568**	**100.0%**	**7.62 [4.24; 11.00]**
**Prediction interval**								**[-3.50; 18.74]**

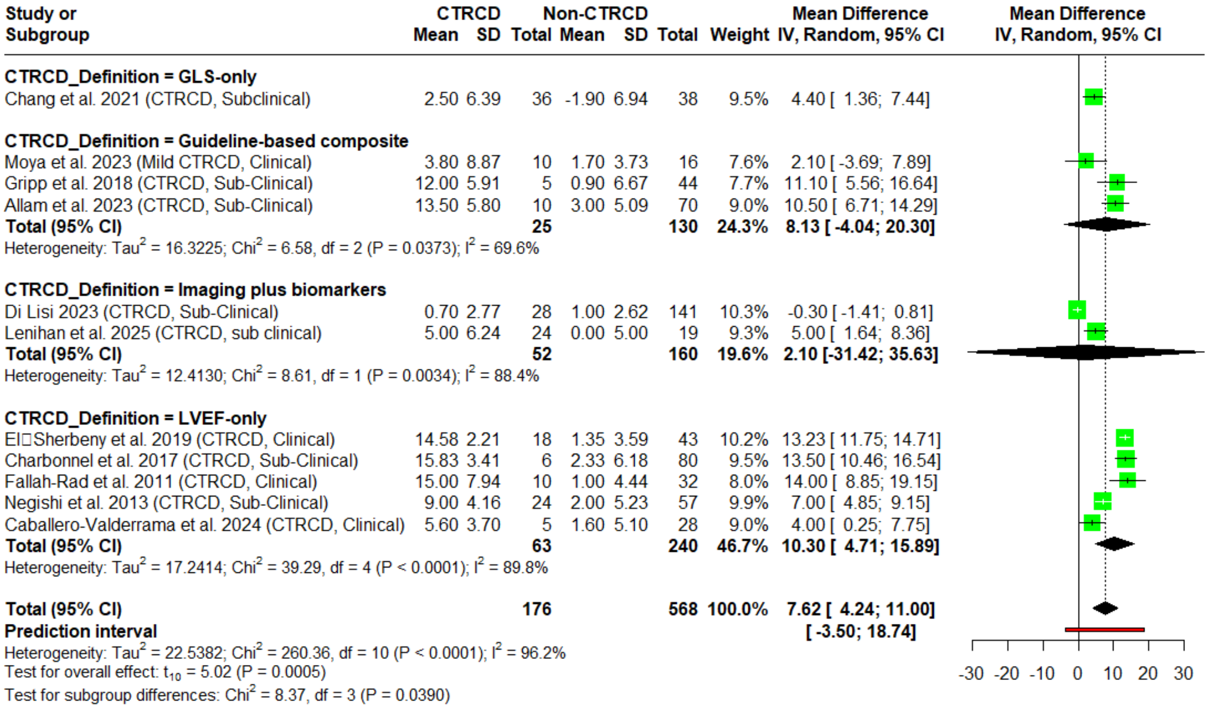

Heterogeneity: Tau^2 = 22.5382; Chi^2 = 260.36, df = 10 (P < 0.0001); I^2 = 96.2%
Test for overall effect: t_{10} = 5.02 (P = 0.0005)
Test for subgroup differences: Chi^2 = 8.37, df = 3 (P = 0.0390)

Change in GLS at Visit 1 in CTRCD patients

Study or Subgroup	Follow_up Mean	Follow_up SD	Follow_up Total	Baseline Mean	Baseline SD	Baseline Total	Weight	Mean Difference IV, Random, 95% CI
**CTRCD_Definition = Combined imaging definition**								
AboElMagdRasheed et al. 2022 (CTRCD, Clinical)(Mixture)	-18.20	2.42	50	-19.57	1.66	50	3.4%	1.37 [0.56; 2.18]
Yang et al. 2024 (CTRCD, Sub-Clinical)(Mixture)	-21.50	2.40	51	-21.80	2.60	51	3.2%	0.30 [-0.67; 1.27]
Tahir et al. 2022 (CTRCD, Sub-Clinical) (Epirubicin□based (CMR))(Mixture)	-17.00	2.00	39	-18.00	2.00	39	3.3%	1.00 [0.11; 1.89]
Tahir et al. 2022 (CTRCD, Sub-Clinical) (Radiotherapy (CMR)(Mixture)	-18.00	2.00	27	-18.00	2.00	27	3.1%	0.00 [-1.07; 1.07]
**Total (95% CI)**			**167**			**167**	**12.9%**	**0.73 [-0.27; 1.73]**
Heterogeneity: $Tau^2 = 0.1684$; $Chi^2 = 5.25$, df = 3 (P = 0.1545); $I^2 = 42.8\%$								
**CTRCD_Definition = GLS-only**								
Mele et al. 2016 (CTRCD, Sub-Clinical)(Mixture)	-21.50	1.60	15	-21.40	1.70	15	3.0%	-0.10 [-1.28; 1.08]
Chang et al. 2021 (CTRCD, Subclinical)	-17.50	2.30	36	-20.10	2.60	36	3.0%	2.60 [1.47; 3.73]
Stoodley et al., 2013 (CTRCD, Sub-Clinical)(Mixture)	-17.50	2.30	50	-19.00	2.30	78	3.3%	1.50 [0.68; 2.32]
Liu et al.2022 (CTRCD, Sub-Clinical) (anthracycline without trastuzumab)(Mixture)	-20.66	1.21	34	-24.00	0.74	34	3.6%	3.34 [2.86; 3.82]
Liu et al.2022 (CTRCD, Sub-Clinical) (trastuzumab without anthracycline)(Mixture)	-23.54	0.88	33	-24.07	0.98	33	3.6%	0.53 [0.08; 0.98]
**Total (95% CI)**			**168**			**196**	**16.6%**	**1.60 [-0.17; 3.36]**
Heterogeneity: $Tau^2 = 1.8383$; $Chi^2 = 82.98$, df = 4 (P < 0.0001); $I^2 = 95.2\%$								
**CTRCD_Definition = Guideline-based composite**								
Moya et al. 2023 (Mild CTRCD, Clinical)	-20.70	3.60	10	-21.90	1.60	10	1.7%	1.20 [-1.24; 3.64]
Moya et al. 2023 (Moderate CTRCD, Clinical)	-19.50	3.10	9	-19.10	1.70	9	1.8%	-0.40 [-2.71; 1.91]
Guan et al. 2021 (CTRCD, Sub-Clinical) (Mixture)	-19.79	2.38	79	-22.60	1.77	79	3.5%	2.81 [2.16; 3.46]
Gripp et al. 2018 (CTRCD, Sub-Clinical)	-15.20	2.00	5	-19.30	1.20	5	2.1%	4.10 [2.06; 6.14]
Cadeddu et al. 2017 (CTRCD, Clinical)(Mixture)	-15.70	1.30	45	-17.60	1.30	45	3.6%	1.90 [1.36; 2.44]
BenKridis et al. 2020 (CTRCD, Clinical)(Mixture)	-19.40	2.16	50	-20.71	2.30	50	3.3%	1.31 [0.44; 2.18]
Allam et al. 2023 (CTRCD, Sub-Clinical)	-14.00	0.40	10	-17.70	3.10	10	2.2%	3.70 [1.76; 5.64]
Kar et al. 2023 (CTRCD, Sub-Clinical)(Mixture)	-16.20	2.60	32	-18.60	2.60	32	2.9%	2.40 [1.13; 3.67]
Li et al. 2024 (CTRCD, Sub-Clinical)(Mixure)	-20.44	2.62	14	-22.77	2.45	80	2.7%	2.33 [0.86; 3.80]
**Total (95% CI)**			**254**			**320**	**23.7%**	**2.19 [1.36; 3.02]**
Heterogeneity: $Tau^2 = 0.4358$; $Chi^2 = 19.71$, df = 8 (P = 0.0115); $I^2 = 59.4\%$								
**CTRCD_Definition = Imaging plus biomarkers**								
Di Lisi 2023 (CTRCD, Sub-Clinical)	-17.00	1.60	28	-21.90	2.10	28	3.2%	4.90 [3.92; 5.88]
Di Lisi 2023 (CTRCD, Sub-Clinical)(Mixture)	-18.45	1.82	61	-20.36	1.47	61	3.5%	1.91 [1.32; 2.50]
Zhang et al. 2022 (CTRCD, Clinical)(Mixture)	-19.60	2.38	207	-20.90	2.34	207	3.6%	1.30 [0.85; 1.75]
**Total (95% CI)**			**296**			**296**	**10.4%**	**2.67 [-2.08; 7.42]**
Heterogeneity: $Tau^2 = 3.4744$; $Chi^2 = 42.81$, df = 2 (P < 0.0001); $I^2 = 95.3\%$								
**CTRCD_Definition = LVEF-only**								
El□Sherbeny et al. 2019 (CTRCD, Clinical)	-16.89	0.80	18	-20.07	1.04	18	3.5%	3.18 [2.57; 3.79]
Charbonnel et al. 2017 (CTRCD, Sub-Clinical)	-18.96	1.71	6	-18.96	1.71	6	2.2%	0.00 [-1.94; 1.94]
Caballero-Valderrama et al. 2024 (CTRCD, Clinical)	-21.60	1.06	5	-22.94	1.46	5	2.5%	1.34 [-0.24; 2.92]
Hazim et al. 2023 (CTRCD, Subclinical) (with anthracycline) (Mixture)	-21.80	2.00	20	-21.30	2.00	20	2.9%	-0.50 [-1.74; 0.74]
Hazim et al. 2023 (CTRCD, Subclinical) (without anthracycline) (Mixture)	-21.80	2.00	27	-20.50	8.00	27	1.3%	-1.30 [-4.41; 1.81]
Bhagat et al. 2023 (CTRCD, Sub-Clinical) (Mixture)	-19.23	3.04	28	-19.43	2.56	31	2.7%	0.20 [-1.24; 1.64]
Park et al., 2020 (CTRCD, Clinical) (Mixture)	-18.80	2.30	13	-21.00	2.40	72	2.8%	2.20 [0.83; 3.57]
Mornos et al. 2013 (CTRCD, Clinical)(Mixture)	-19.00	2.40	74	-21.20	2.50	74	3.4%	2.20 [1.41; 2.99]
Anqi et al. 2019 (CTRCD, Sub-Clinical)(Mixture)	-18.42	2.51	40	-19.70	2.46	40	3.1%	1.28 [0.19; 2.37]
Fallah-Rad et al. 2011 (CTRCD, Clinical)	-16.40	1.10	10	-19.80	1.80	10	2.8%	3.40 [2.09; 4.71]
Negishi et al. 2013 (CTRCD, Sub-Clinical)	-18.30	2.10	24	-20.70	2.60	24	2.8%	2.40 [1.06; 3.74]
**Total (95% CI)**			**265**			**327**	**30.0%**	**1.51 [0.54; 2.49]**
Heterogeneity: $Tau^2 = 1.5090$; $Chi^2 = 51.93$, df = 10 (P < 0.0001); $I^2 = 80.7\%$								
**CTRCD_Definition = Not reported**								
Huynh et al. 2025 (CTRCD, Sub-Clinical)(Mixture)	-19.93	1.00	11	-21.80	1.61	16	3.2%	1.87 [0.88; 2.86]
Kersten et al. 2023 (CTRCD, Clinical)(Mixture)	-13.60	1.90	33	-14.90	2.10	34	3.2%	1.30 [0.34; 2.26]
**Total (95% CI)**			**44**			**50**	**6.4%**	**1.58 [-2.04; 5.20]**
Heterogeneity: $Tau^2 = 0$; $Chi^2 = 0.66$, df = 1 (P = 0.4164); $I^2 = 0\%$								
**Total (95% CI)**			**1194**			**1356**	**100.0%**	**1.70 [1.24; 2.17]**
**Prediction interval**								**[-0.67; 4.08]**

Heterogeneity: $Tau^2 = 1.3171$; $Chi^2 = 230.02$, df = 33 (P < 0.0001); $I^2 = 85.7\%$
Test for overall effect: $t_{33} = 7.45$ (P < 0.0001)
Test for subgroup differences: $Chi^2 = 10.91$, df = 5 (P = 0.0533)

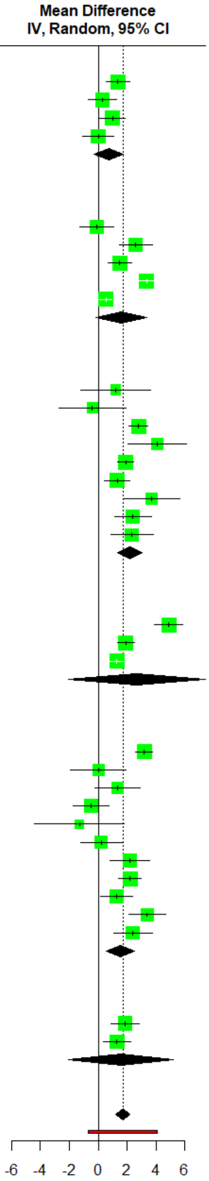

Change in GLS at Visit 2 in CTRCD patients

Study or Subgroup	Follow_up Mean	SD	Total	Baseline Mean	SD	Total	Weight	Mean Difference IV, Random, 95% CI
**CTRCD_Definition = Combined imaging definition**								
Yang et al. 2024 (CTRCD, Sub-Clinical)(Mixture)	-21.60	2.20	51	-21.80	2.60	51	6.9%	0.20 [-0.73; 1.13]
**CTRCD_Definition = GLS-only**								
Mele et al. 2016 (CTRCD, Sub-Clinical)(Mixture)	-20.60	2.00	15	-21.40	1.70	15	6.4%	0.80 [-0.53; 2.13]
Chang et al. 2021 (CTRCD, Subclinical)	-16.30	2.20	36	-20.10	2.60	36	6.7%	3.80 [2.69; 4.91]
Stoodley et al., 2013 (CTRCD, Sub-Clinical)(Mixture)	-18.20	2.20	50	-19.00	2.30	78	7.1%	0.80 [0.00; 1.60]
**Total (95% CI)**			**101**			**129**	**20.2%**	**1.80 [-2.50; 6.09]**
Heterogeneity: $Tau^2 = 2.6826$; $Chi^2 = 20.3$, df = 2 ($P < 0.0001$); $I^2 = 90.1\%$								
**CTRCD_Definition = Guideline-based composite**								
Moya et al. 2023 (Mild CTRCD, Clinical)	-19.30	2.30	10	-21.90	1.60	10	5.9%	2.60 [0.86; 4.34]
Moya et al. 2023 (Moderate CTRCD, Clinical)	-15.60	3.20	9	-19.10	1.70	9	5.0%	3.50 [1.13; 5.87]
Guan et al. 2021 (CTRCD, Sub-Clinical) (Mixture)	-18.17	3.04	79	-22.60	1.77	79	7.1%	4.43 [3.65; 5.21]
Cadeddu et al. 2017 (CTRCD, Clinical)(Mixture)	-15.90	1.20	45	-17.60	1.30	45	7.3%	1.70 [1.18; 2.22]
BenKridis et al. 2020 (CTRCD, Clinical)(Mixture)	-18.37	2.70	50	-20.71	2.30	50	6.9%	2.34 [1.36; 3.32]
**Total (95% CI)**			**193**			**193**	**32.1%**	**2.86 [1.43; 4.28]**
Heterogeneity: $Tau^2 = 1.1712$; $Chi^2 = 33.74$, df = 4 ($P < 0.0001$); $I^2 = 88.1\%$								
**CTRCD_Definition = LVEF-only**								
El□Sherbeny et al. 2019 (CTRCD, Clinical)	-14.74	1.05	18	-20.07	1.04	18	7.2%	5.33 [4.65; 6.01]
Charbonnel et al. 2017 (CTRCD, Sub-Clinical)	-17.40	2.76	6	-18.96	1.71	6	4.6%	1.56 [-1.04; 4.16]
Caballero-Valderrama et al. 2024 (CTRCD, Clinical)	-18.32	1.40	5	-22.94	1.46	5	5.8%	4.62 [2.85; 6.39]
Hazim et al. 2023 (CTRCD, Subclinical) (with anthracycline) (Mixture)	-21.30	2.00	20	-21.30	2.00	20	6.6%	0.00 [-1.24; 1.24]
Hazim et al. 2023 (CTRCD, Subclinical) (without anthracycline) (Mixture)	-21.40	2.00	27	-20.50	8.00	27	4.0%	-0.90 [-4.01; 2.21]
Anqi et al. 2019 (CTRCD, Sub-Clinical)(Mixture)	-16.66	3.18	40	-19.70	2.46	40	6.5%	3.04 [1.79; 4.29]
Fallah-Rad et al. 2011 (CTRCD, Clinical)	-15.40	1.80	10	-19.80	1.80	10	6.1%	4.40 [2.82; 5.98]
**Total (95% CI)**			**126**			**126**	**40.8%**	**2.76 [0.56; 4.95]**
Heterogeneity: $Tau^2 = 4.7075$; $Chi^2 = 69.83$, df = 6 ($P < 0.0001$); $I^2 = 91.4\%$								
**Total (95% CI)**			**471**			**499**	**100.0%**	**2.46 [1.47; 3.44]**
**Prediction interval**								**[-1.30; 6.22]**

Heterogeneity: $Tau^2 = 2.8952$; $Chi^2 = 184.49$, df = 15 ($P < 0.0001$); $I^2 = 91.9\%$
Test for overall effect: $t_{15} = 5.30$ ($P < 0.0001$)
Test for subgroup differences: $Chi^2 = 16.32$, df = 3 ($P = 0.0010$)

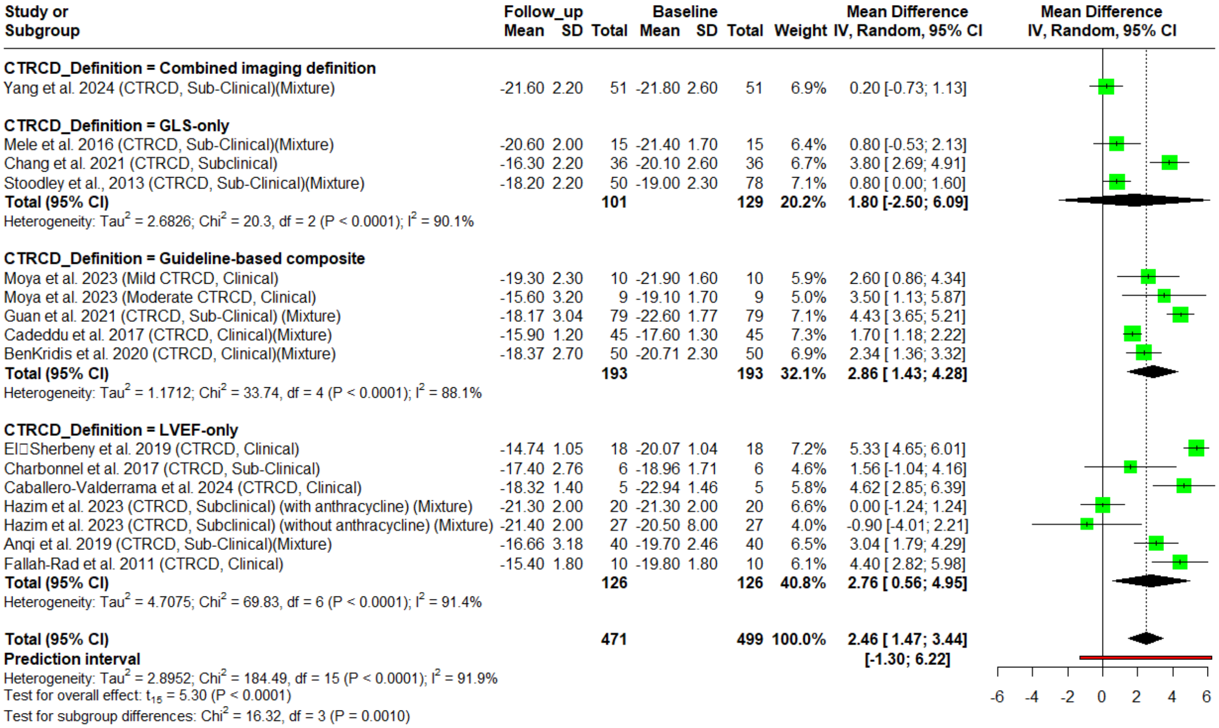

Change in GLS at Visit 3 in CTRCD patients

Study or Subgroup	Follow_up Mean	SD	Total	Baseline Mean	SD	Total	Weight	Mean Difference IV, Random, 95% CI
**CTRCD_Definition = Combined imaging definition**								
Yang et al. 2024 (CTRCD, Sub-Clinical)(Mixture)	-21.80	2.30	51	-21.80	2.60	51	11.4%	0.00 [-0.95; 0.95]
**CTRCD_Definition = Guideline-based composite**								
Guan et al. 2021 (CTRCD, Sub-Clinical) (Mixture)	-16.96	3.65	79	-22.60	1.77	79	11.5%	5.64 [4.75; 6.53]
Cadeddu et al. 2017 (CTRCD, Clinical)(Mixture)	-15.60	0.90	45	-17.60	1.30	45	11.6%	2.00 [1.54; 2.46]
BenKridis et al. 2020 (CTRCD, Clinical)(Mixture)	-15.33	1.15	50	-20.71	2.30	50	11.6%	5.38 [4.67; 6.09]
**Total (95% CI)**			**174**			**174**	**34.7%**	**4.32 [-0.75; 9.38]**
Heterogeneity: $Tau^2 = 4.0616$; $Chi^2 = 89$, df = 2 ($P < 0.0001$); $I^2 = 97.8\%$								
**CTRCD_Definition = LVEF-only**								
El□Sherbeny et al. 2019 (CTRCD, Clinical)	-12.29	1.38	18	-20.07	1.04	18	11.5%	7.78 [6.98; 8.58]
Charbonnel et al. 2017 (CTRCD, Sub-Clinical)	-15.66	1.04	6	-18.96	1.71	6	10.9%	3.30 [1.70; 4.90]
Hazim et al. 2023 (CTRCD, Subclinical) (with anthracycline) (Mixture)	-20.80	2.00	20	-21.30	2.00	20	11.2%	0.50 [-0.74; 1.74]
Hazim et al. 2023 (CTRCD, Subclinical) (without anthracycline) (Mixture)	-21.20	1.00	27	-20.50	8.00	27	9.3%	-0.70 [-3.74; 2.34]
Fallah-Rad et al. 2011 (CTRCD, Clinical)	-12.40	2.10	10	-19.80	1.80	10	10.8%	7.40 [5.69; 9.11]
**Total (95% CI)**			**81**			**81**	**53.9%**	**3.76 [-1.02; 8.55]**
Heterogeneity: $Tau^2 = 13.8929$; $Chi^2 = 121.38$, df = 4 ($P < 0.0001$); $I^2 = 96.7\%$								
**Total (95% CI)**			**306**			**306**	**100.0%**	**3.55 [1.10; 6.00]**
**Prediction interval**								**[-3.95; 11.06]**

Heterogeneity: $Tau^2 = 9.4815$; $Chi^2 = 300.90$, df = 8 ($P < 0.0001$); $I^2 = 97.3\%$
Test for overall effect: $t_8 = 3.35$ ($P = 0.0101$)
Test for subgroup differences: $Chi^2 = 14.59$, df = 2 ($P = 0.0007$)

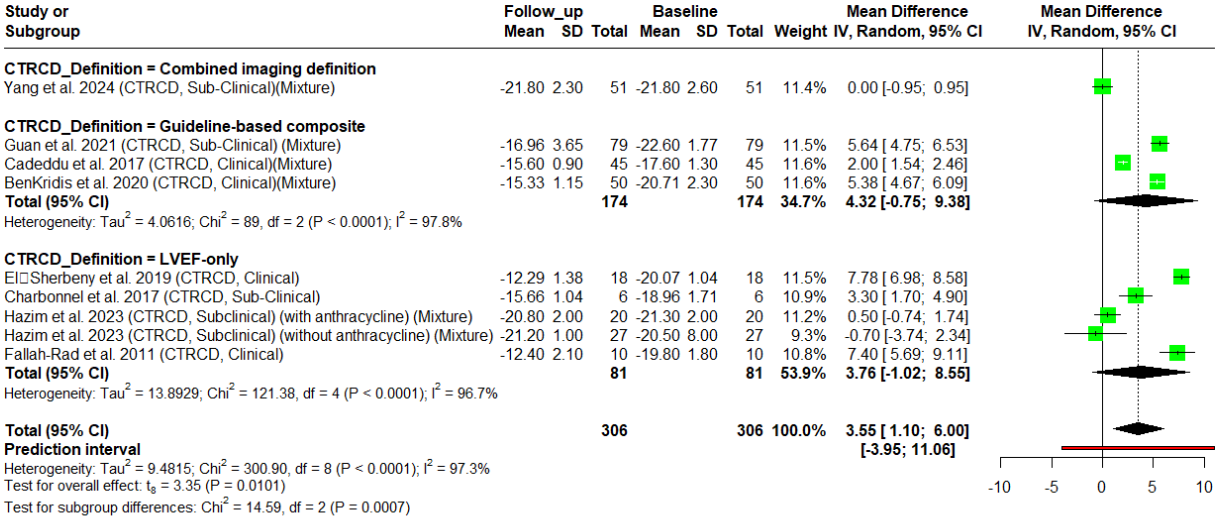

Change in GLS at Last Visit in CTRCD patients

Study or Subgroup	Follow_up Mean	SD	Total	Baseline Mean	SD	Total	Weight	Mean Difference IV, Random, 95% CI
**CTRCD_Definition = Combined imaging definition**								
AboElMagdRasheed et al. 2022 (CTRCD, Clinical)(Mixture)	-17.84	2.94	50	-19.57	1.66	50	2.9%	1.73 [0.79; 2.67]
Yang et al. 2024 (CTRCD, Sub-Clinical)(Mixture)	-20.10	2.50	51	-21.80	2.60	51	2.9%	1.70 [0.71; 2.69]
Tahir et al. 2022 (CTRCD, Sub-Clinical) (Epirubicin□based (CMR))(Mixture)	-17.00	2.00	38	-18.00	2.00	39	2.9%	1.00 [0.11; 1.89]
Tahir et al. 2022 (CTRCD, Sub-Clinical) (Radiotherapy (CMR)(Mixture)	-18.00	1.00	27	-18.00	2.00	27	2.9%	0.00 [-0.84; 0.84]
Cheng et al. 2023 (CTRCD, Clinical)(Mixture)	-15.00	2.00	24	-16.00	1.60	24	2.8%	1.00 [-0.02; 2.02]
**Total (95% CI)**			**190**			**191**	**14.4%**	**1.06 [0.17; 1.95]**
Heterogeneity: Tau^2 = 0.3109; Chi^2 = 9.64, df = 4 (P = 0.0469); I^2 = 58.5%								
**CTRCD_Definition = GLS-only**								
Mele et al. 2016 (CTRCD, Sub-Clinical)(Mixture)	-20.90	1.60	15	-21.40	1.70	15	2.8%	0.50 [-0.68; 1.68]
Chang et al. 2021 (CTRCD, Subclinical)	-18.20	2.30	36	-20.10	2.60	36	2.8%	1.90 [0.77; 3.03]
Stoodley et al., 2013 (CTRCD, Sub-Clinical)(Mixture)	-19.10	1.90	50	-19.00	2.30	78	3.0%	-0.10 [-0.83; 0.63]
Liu et al.2022 (CTRCD, Sub-Clinical) (anthracycline without trastuzumab)(Mixture)	-19.93	0.78	34	-24.00	0.74	34	3.1%	4.07 [3.71; 4.43]
Liu et al.2022 (CTRCD, Sub-Clinical) (trastuzumab without anthracycline)(Mixture)	-20.55	1.58	33	-24.07	0.98	33	3.0%	3.52 [2.89; 4.15]
**Total (95% CI)**			**168**			**196**	**14.7%**	**2.01 [-0.27; 4.29]**
Heterogeneity: Tau^2 = 3.2405; Chi^2 = 125.38, df = 4 (P < 0.0001); I^2 = 96.8%								
**CTRCD_Definition = Guideline-based composite**								
Moya et al. 2023 (Mild CTRCD, Clinical)	-17.80	2.50	10	-21.90	1.60	10	2.3%	4.10 [2.26; 5.94]
Moya et al. 2023 (Moderate CTRCD, Clinical)	-16.40	4.40	9	-19.10	1.70	9	1.6%	2.70 [-0.38; 5.78]
Guan et al. 2021 (CTRCD, Sub-Clinical) (Mixture)	-15.94	4.03	79	-22.60	1.77	79	2.9%	6.66 [5.69; 7.63]
Gripp et al. 2018 (CTRCD, Sub-Clinical)	-15.60	1.10	5	-19.30	1.20	5	2.6%	3.70 [2.27; 5.13]
Cadeddu et al. 2017 (CTRCD, Clinical)(Mixture)	-14.40	1.10	45	-17.60	1.30	45	3.1%	3.20 [2.70; 3.70]
BenKridis et al. 2020 (CTRCD, Clinical)(Mixture)	-16.42	1.40	50	-20.71	2.30	50	3.0%	4.29 [3.54; 5.04]
Allam et al. 2023 (CTRCD, Sub-Clinical)	-11.30	0.80	10	-17.70	3.10	10	2.3%	6.40 [4.42; 8.38]
Kar et al. 2023 (CTRCD, Sub-Clinical)(Mixture)	-16.00	2.60	32	-18.60	2.60	32	2.7%	2.60 [1.33; 3.87]
Li et al. 2024 (CTRCD, Sub-Clinical)(Mixure)	-18.83	2.08	14	-22.77	2.45	80	2.7%	3.94 [2.73; 5.15]
**Total (95% CI)**			**254**			**320**	**23.2%**	**4.21 [3.11; 5.32]**
Heterogeneity: Tau^2 = 1.5767; Chi^2 = 50.42, df = 8 (P < 0.0001); I^2 = 84.1%								
**CTRCD_Definition = Imaging plus biomarkers**								
Di Lisi 2023 (CTRCD, Sub-Clinical)	-17.30	2.20	28	-21.90	2.10	28	2.8%	4.60 [3.47; 5.73]
Di Lisi 2023 (CTRCD, Sub-Clinical)(Mixture)	-17.22	1.62	61	-20.36	1.47	61	3.1%	3.14 [2.59; 3.69]
Zhang et al. 2022 (CTRCD, Clinical)(Mixture)	-18.75	4.45	145	-20.90	2.34	207	3.0%	2.15 [1.36; 2.94]
Lenihan et al. 2025 (CTRCD, Clinical) Sub-CTX	-18.00	1.00	24	-20.00	2.00	24	2.9%	2.00 [1.11; 2.89]
Lenihan et al. 2025 (CTRCD, Clinical) CTX	-17.00	1.00	9	-19.00	2.00	9	2.6%	2.00 [0.54; 3.46]
**Total (95% CI)**			**267**			**329**	**14.3%**	**2.78 [1.43; 4.14]**
Heterogeneity: Tau^2 = 0.8929; Chi^2 = 18.09, df = 4 (P = 0.0012); I^2 = 77.9%								
**CTRCD_Definition = LVEF-only**								
El□Sherbeny et al. 2019 (CTRCD, Clinical)	-15.68	1.52	18	-20.07	1.04	18	2.9%	4.39 [3.54; 5.24]
Charbonnel et al. 2017 (CTRCD, Sub-Clinical)	-16.76	1.04	6	-18.96	1.71	6	2.5%	2.20 [0.60; 3.80]
Caballero-Valderrama et al. 2024 (CTRCD, Clinical)	-20.86	1.88	5	-22.94	1.46	5	2.2%	2.08 [-0.01; 4.17]
Hazim et al. 2023 (CTRCD, Subclinical) (with anthracycline) (Mixture)	-19.80	1.00	20	-21.30	2.00	20	2.9%	1.50 [0.52; 2.48]
Hazim et al. 2023 (CTRCD, Subclinical) (without anthracycline) (Mixture)	-21.30	1.00	27	-20.50	8.00	27	1.6%	-0.80 [-3.84; 2.24]
Bhagat et al. 2023 (CTRCD, Sub-Clinical) (Mixture)	-18.73	3.66	29	-19.43	2.56	31	2.5%	0.70 [-0.91; 2.31]
Park et al., 2020 (CTRCD, Clinical) (Mixture)	-15.30	2.60	13	-21.00	2.40	72	2.6%	5.70 [4.18; 7.22]
Mornos et al. 2013 (CTRCD, Clinical)(Mixture)	-17.20	2.80	74	-21.20	2.50	74	2.9%	4.00 [3.14; 4.86]
Anqi et al. 2019 (CTRCD, Sub-Clinical)(Mixture)	-15.16	2.13	40	-19.70	2.46	40	2.9%	4.54 [3.53; 5.55]
Fallah-Rad et al. 2011 (CTRCD, Clinical)	-15.90	2.70	10	-19.80	1.80	10	2.2%	3.90 [1.89; 5.91]
Negishi et al. 2013 (CTRCD, Sub-Clinical)	-18.30	2.60	24	-20.70	2.60	24	2.6%	2.40 [0.93; 3.87]
**Total (95% CI)**			**266**			**327**	**27.8%**	**2.95 [1.73; 4.17]**
Heterogeneity: Tau^2 = 2.4531; Chi^2 = 59.08, df = 10 (P < 0.0001); I^2 = 83.1%								
**CTRCD_Definition = Not reported**								
Huynh et al. 2025 (CTRCD, Sub-Clinical)(Mixture)	-20.00	1.00	11	-21.80	1.61	16	2.9%	1.80 [0.81; 2.79]
Kersten et al. 2023 (CTRCD, Clinical)(Mixture)	-13.80	2.50	32	-14.90	2.10	34	2.8%	1.10 [-0.02; 2.22]
**Total (95% CI)**			**43**			**50**	**5.7%**	**1.49 [-2.92; 5.91]**
Heterogeneity: Tau^2 = 0; Chi^2 = 0.85, df = 1 (P = 0.3572); I^2 = 0%								
**Total (95% CI)**			**1188**			**1413**	**100.0%**	**2.73 [2.15; 3.31]**
**Prediction interval**								**[-0.58; 6.03]**
Heterogeneity: Tau^2 = 2.5725; Chi^2 = 402.54, df = 36 (P < 0.0001); I^2 = 91.1%								
Test for overall effect: t_{36} = 9.54 (P < 0.0001)								
Test for subgroup differences: Chi^2 = 37.43, df = 5 (P < 0.0001)								

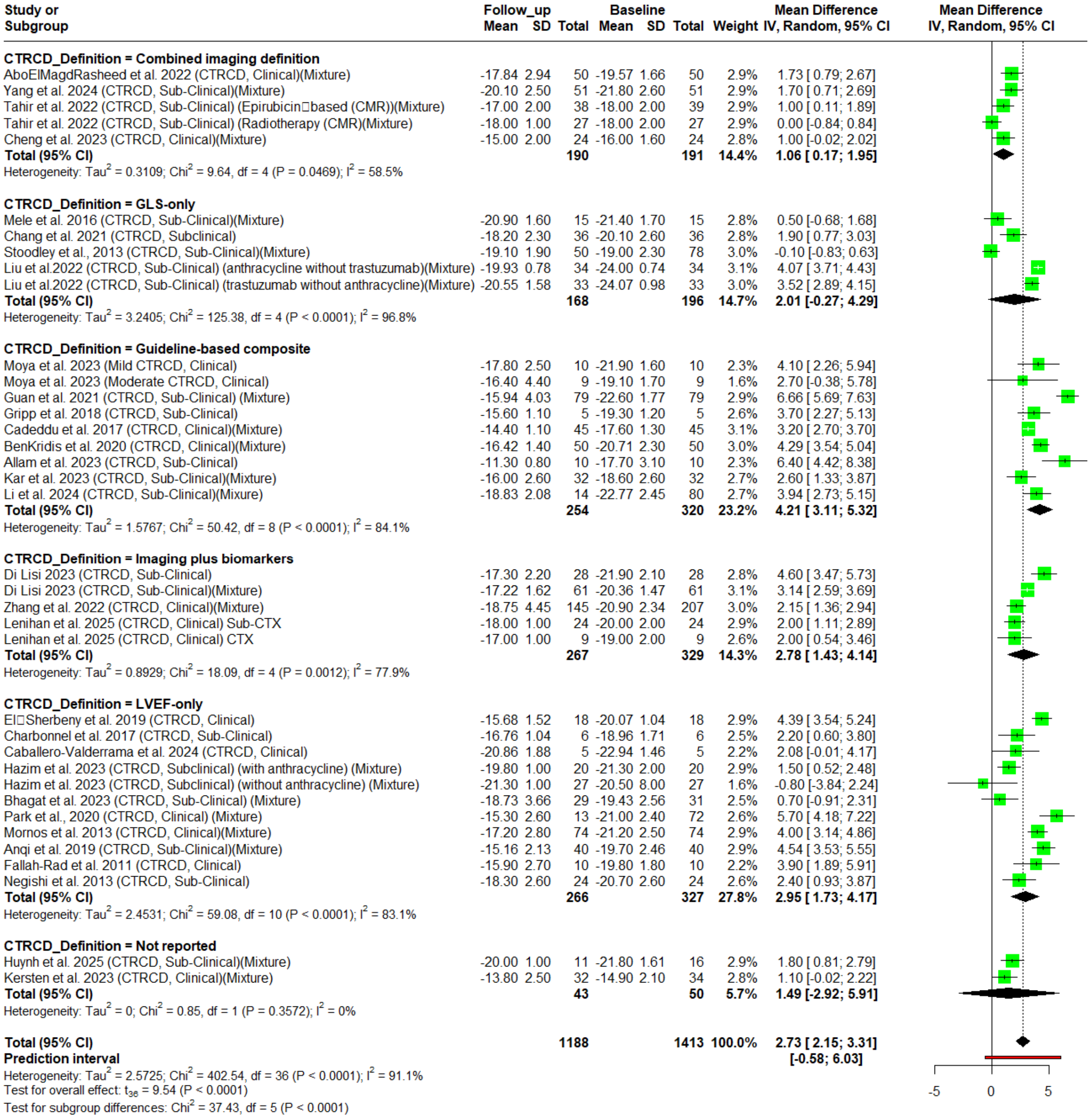

Change in LVEF at Visit 1 in CTRCD patients

Study or Subgroup	Follow_up Mean	SD	Total	Baseline Mean	SD	Total	Weight	Mean Difference IV, Fixed, 95% CI
**CTRCD_Definition = Combined imaging definition**								
AboElMagdRasheed et al. 2022 (CTRCD, Clinical)(Mixture)	64.10	5.46	50	65.70	4.88	50	2.6%	-1.60 [-3.63; 0.43]
Yang et al. 2024 (CTRCD, Sub-Clinical)(Mixture)	58.40	5.20	51	59.70	6.50	51	2.0%	-1.30 [-3.58; 0.98]
Tahir et al. 2022 (CTRCD, Sub-Clinical) (Epirubicin□based (CMR))(Mixture)	60.00	6.00	39	60.00	5.00	39	1.8%	0.00 [-2.45; 2.45]
Tahir et al. 2022 (CTRCD, Sub-Clinical) (Radiotherapy (CMR)(Mixture)	64.00	6.00	27	62.00	5.00	27	1.2%	2.00 [-0.95; 4.95]
**Total (95% CI)**			**167**			**167**	**7.6%**	**-0.57 [-1.75; 0.61]**
Heterogeneity: $Tau^2 = 0.6035$; $Chi^2 = 4.51$, df = 3 (P = 0.2112); $I^2 = 33.5\%$								
**CTRCD_Definition = GLS-only**								
Mele et al. 2016 (CTRCD, Sub-Clinical)(Mixture)	63.00	3.00	15	63.00	3.00	15	2.3%	0.00 [-2.15; 2.15]
Chang et al. 2021 (CTRCD, Subclinical)	69.30	10.20	36	70.20	5.70	36	0.7%	-0.90 [-4.72; 2.92]
Stoodley et al., 2013 (CTRCD, Sub-Clinical)(Mixture)	57.00	3.00	50	58.00	3.00	78	9.4%	-1.00 [-2.07; 0.07]
Liu et al.2022 (CTRCD, Sub-Clinical) (anthracycline without trastuzumab)(Mixture)	67.36	4.29	34	66.78	4.17	34	2.6%	0.58 [-1.43; 2.59]
Liu et al.2022 (CTRCD, Sub-Clinical) (trastuzumab without anthracycline)(Mixture)	66.03	3.51	33	66.14	2.82	33	4.5%	-0.11 [-1.65; 1.43]
**Total (95% CI)**			**168**			**196**	**19.6%**	**-0.46 [-1.20; 0.28]**
Heterogeneity: $Tau^2 = 0$; $Chi^2 = 2.44$, df = 4 (P = 0.6552); $I^2 = 0\%$								
**CTRCD_Definition = Guideline-based composite**								
Moya et al. 2023 (Mild CTRCD, Clinical)	55.80	4.40	10	57.40	3.60	10	0.9%	-1.60 [-5.12; 1.92]
Moya et al. 2023 (Moderate CTRCD, Clinical)	54.80	8.70	9	55.00	4.50	9	0.3%	-0.20 [-6.60; 6.20]
Guan et al. 2021 (CTRCD, Sub-Clinical) (Mixture)	65.57	5.25	79	66.65	4.13	79	4.9%	-1.08 [-2.55; 0.39]
Gripp et al. 2018 (CTRCD, Sub-Clinical)	57.60	12.30	5	64.00	4.80	5	0.1%	-6.40 [-17.97; 5.17]
Cadeddu et al. 2017 (CTRCD, Clinical)(Mixture)	63.20	2.10	45	62.80	2.00	45	14.9%	0.40 [-0.45; 1.25]
BenKridis et al. 2020 (CTRCD, Clinical)(Mixture)	60.74	4.45	50	62.48	3.04	50	4.8%	-1.74 [-3.23; -0.25]
Allam et al. 2023 (CTRCD, Sub-Clinical)	53.00	3.30	10	61.20	5.11	10	0.8%	-8.20 [-11.97; -4.43]
Kar et al. 2023 (CTRCD, Sub-Clinical)(Mixture)	57.90	7.20	32	58.50	6.00	32	1.0%	-0.60 [-3.85; 2.65]
Li et al. 2024 (CTRCD, Sub-Clinical)(Mixure)	59.80	4.81	14	61.30	4.73	80	1.4%	-1.50 [-4.22; 1.22]
**Total (95% CI)**			**254**			**320**	**29.0%**	**-0.64 [-1.25; -0.03]**
Heterogeneity: $Tau^2 = 3.1178$; $Chi^2 = 25.3$, df = 8 (P = 0.0014); $I^2 = 68.4\%$								
**CTRCD_Definition = Imaging plus biomarkers**								
Di Lisi 2023 (CTRCD, Sub-Clinical)	59.20	4.00	28	60.10	1.50	28	4.3%	-0.90 [-2.48; 0.68]
Di Lisi 2023 (CTRCD, Sub-Clinical)(Mixture)	59.89	3.14	61	60.00	3.21	61	8.4%	-0.11 [-1.24; 1.02]
Zhang et al. 2022 (CTRCD, Clinical)(Mixture)	57.54	6.18	207	60.92	5.78	207	8.0%	-3.38 [-4.53; -2.23]
**Total (95% CI)**			**296**			**296**	**20.7%**	**-1.54 [-2.26; -0.82]**
Heterogeneity: $Tau^2 = 2.6362$; $Chi^2 = 16.6$, df = 2 (P = 0.0002); $I^2 = 88\%$								
**CTRCD_Definition = LVEF-only**								
El□Sherbeny et al. 2019 (CTRCD, Clinical)	59.65	2.76	18	62.82	2.19	18	4.0%	-3.17 [-4.80; -1.54]
Charbonnel et al. 2017 (CTRCD, Sub-Clinical)	64.80	2.86	6	64.83	2.86	6	1.0%	-0.03 [-3.27; 3.21]
Caballero-Valderrama et al. 2024 (CTRCD, Clinical)	60.60	1.00	5	62.90	3.70	5	0.9%	-2.30 [-5.66; 1.06]
Hazim et al. 2023 (CTRCD, Subclinical) (with anthracycline) (Mixture)	59.90	3.00	20	63.00	4.00	20	2.2%	-3.10 [-5.29; -0.91]
Hazim et al. 2023 (CTRCD, Subclinical) (without anthracycline) (Mixture)	63.80	4.00	27	63.00	3.00	27	3.0%	0.80 [-1.09; 2.69]
Bhagat et al. 2023 (CTRCD, Sub-Clinical) (Mixture)	60.30	3.90	28	62.66	3.88	31	2.7%	-2.36 [-4.35; -0.37]
Park et al., 2020 (CTRCD, Clinical) (Mixture)	64.50	7.40	13	65.00	6.40	72	0.6%	-0.50 [-4.79; 3.79]
Mornos et al. 2013 (CTRCD, Clinical)(Mixture)	60.30	6.00	74	61.00	5.60	74	3.1%	-0.70 [-2.57; 1.17]
Anqi et al. 2019 (CTRCD, Sub-Clinical)(Mixture)	67.71	5.85	40	69.76	6.13	40	1.5%	-2.05 [-4.68; 0.58]
Fallah-Rad et al. 2011 (CTRCD, Clinical)	58.00	4.00	10	64.00	3.00	10	1.1%	-6.00 [-9.10; -2.90]
Negishi et al. 2013 (CTRCD, Sub-Clinical)	58.00	5.50	24	64.00	4.60	24	1.3%	-6.00 [-8.87; -3.13]
**Total (95% CI)**			**265**			**327**	**21.5%**	**-2.13 [-2.84; -1.43]**
Heterogeneity: $Tau^2 = 2.9159$; $Chi^2 = 29.06$, df = 10 (P = 0.0012); $I^2 = 65.6\%$								
**CTRCD_Definition = Not reported**								
Huynh et al. 2025 (CTRCD, Sub-Clinical)(Mixture)	64.80	5.25	11	65.20	6.19	16	0.6%	-0.40 [-4.74; 3.94]
Kersten et al. 2023 (CTRCD, Clinical)(Mixture)	61.20	6.70	33	65.20	6.80	34	1.0%	-4.00 [-7.23; -0.77]
**Total (95% CI)**			**44**			**50**	**1.6%**	**-2.71 [-5.31; -0.12]**
Heterogeneity: $Tau^2 = 2.6696$; $Chi^2 = 1.7$, df = 1 (P = 0.1922); $I^2 = 41.2\%$								
**Total (95% CI)**			**1194**			**1356**	**100.0%**	**-1.14 [-1.47; -0.81]**
**Prediction interval**								**[-4.38; 1.50]**
Heterogeneity: $Tau^2 = 1.9858$; $Chi^2 = 96.64$, df = 33 (P < 0.0001); $I^2 = 65.9\%$								
Test for overall effect: Z = -6.84 (P < 0.0001)								
Test for subgroup differences: $Chi^2 = 17.03$, df = 5 (P = 0.0044)								

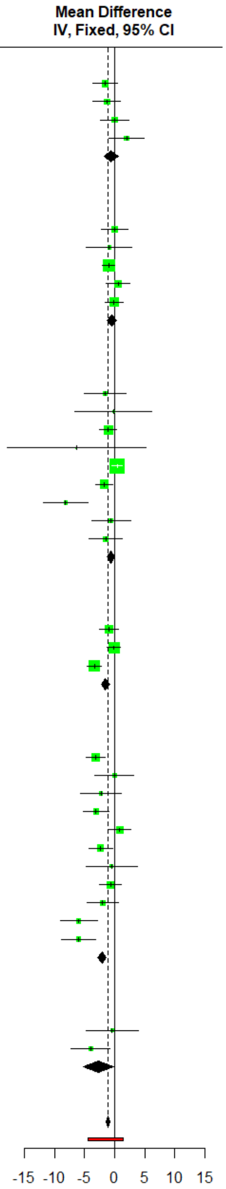

Change in LVEF at Visit 2 in CTRCD patients

Study or Subgroup	Follow_up Mean	Follow_up SD	Follow_up Total	Baseline Mean	Baseline SD	Baseline Total	Weight	Mean Difference IV, Random, 95% CI
**CTRCD_Definition = Combined imaging definition**								
Yang et al. 2024 (CTRCD, Sub-Clinical)(Mixture)	57.80	6.70	51	59.70	6.50	51	6.4%	-1.90 [-4.46; 0.66]
**CTRCD_Definition = GLS-only**								
Mele et al. 2016 (CTRCD, Sub-Clinical)(Mixture)	62.00	3.00	15	63.00	3.00	15	6.5%	-1.00 [-3.15; 1.15]
Chang et al. 2021 (CTRCD, Subclinical)	68.60	8.40	36	70.20	5.70	36	6.2%	-1.60 [-4.92; 1.72]
Stoodley et al., 2013 (CTRCD, Sub-Clinical)(Mixture)	57.00	3.00	50	58.00	3.00	78	6.7%	-1.00 [-2.07; 0.07]
**Total (95% CI)**			**101**			**129**	**19.4%**	**-1.05 [-1.53; -0.56]**
Heterogeneity: $Tau^2 = 0$; $Chi^2 = 0.12$, df = 2 (P = 0.9436); $I^2 = 0\%$								
**CTRCD_Definition = Guideline-based composite**								
Moya et al. 2023 (Mild CTRCD, Clinical)	55.10	4.50	10	57.40	3.60	10	6.1%	-2.30 [-5.87; 1.27]
Moya et al. 2023 (Moderate CTRCD, Clinical)	46.30	4.50	9	55.00	4.50	9	5.9%	-8.70 [-12.86; -4.54]
Guan et al. 2021 (CTRCD, Sub-Clinical) (Mixture)	62.34	5.21	79	66.65	4.13	79	6.6%	-4.31 [-5.78; -2.84]
Cadeddu et al. 2017 (CTRCD, Clinical)(Mixture)	60.80	1.40	45	62.80	2.00	45	6.7%	-2.00 [-2.71; -1.29]
BenKridis et al. 2020 (CTRCD, Clinical)(Mixture)	59.34	5.12	50	62.48	3.04	50	6.6%	-3.14 [-4.79; -1.49]
**Total (95% CI)**			**193**			**193**	**31.9%**	**-3.58 [-6.32; -0.85]**
Heterogeneity: $Tau^2 = 2.4309$; $Chi^2 = 16.62$, df = 4 (P = 0.0023); $I^2 = 75.9\%$								
**CTRCD_Definition = LVEF-only**								
El□Sherbeny et al. 2019 (CTRCD, Clinical)	45.76	3.72	18	62.82	2.19	18	6.5%	-17.06 [-19.05; -15.07]
Charbonnel et al. 2017 (CTRCD, Sub-Clinical)	62.16	4.76	6	64.83	2.86	6	5.8%	-2.67 [-7.11; 1.77]
Caballero-Valderrama et al. 2024 (CTRCD, Clinical)	58.90	5.20	5	62.90	3.70	5	5.3%	-4.00 [-9.59; 1.59]
Hazim et al. 2023 (CTRCD, Subclinical) (with anthracycline) (Mixture)	59.80	3.00	20	63.00	4.00	20	6.5%	-3.20 [-5.39; -1.01]
Hazim et al. 2023 (CTRCD, Subclinical) (without anthracycline) (Mixture)	62.40	3.00	27	63.00	3.00	27	6.6%	-0.60 [-2.20; 1.00]
Anqi et al. 2019 (CTRCD, Sub-Clinical)(Mixture)	63.12	6.02	40	69.76	6.13	40	6.4%	-6.64 [-9.30; -3.98]
Fallah-Rad et al. 2011 (CTRCD, Clinical)	42.00	9.00	10	64.00	3.00	10	5.2%	-22.00 [-27.88; -16.12]
**Total (95% CI)**			**126**			**126**	**42.3%**	**-7.90 [-15.38; -0.41]**
Heterogeneity: $Tau^2 = 60.4478$; $Chi^2 = 198.9$, df = 6 (P < 0.0001); $I^2 = 97\%$								
**Total (95% CI)**			**471**			**499**	**100.0%**	**-4.93 [-8.04; -1.81]**
**Prediction interval**								**[-16.93; 7.08]**
Heterogeneity: $Tau^2 = 29.7186$; $Chi^2 = 283.31$, df = 15 (P < 0.0001); $I^2 = 94.7\%$								
Test for overall effect: $t_{15} = -3.37$ (P = 0.0042)								
Test for subgroup differences: $Chi^2 = 11.90$, df = 3 (P = 0.0077)								

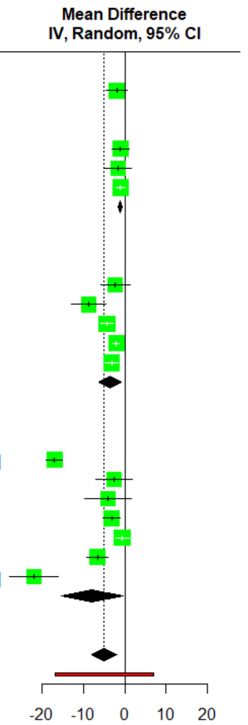

Change in LVEF at Visit 3 in CTRCD patients

Study or Subgroup	Follow_up Mean	Follow_up SD	Follow_up Total	Baseline Mean	Baseline SD	Baseline Total	Weight	Mean Difference IV, Random, 95% CI
**CTRCD_Definition = Combined imaging definition**								
Yang et al. 2024 (CTRCD, Sub-Clinical)(Mixture)	56.70	5.80	51	59.70	6.50	51	11.2%	-3.00 [-5.39; -0.61]
**CTRCD_Definition = Guideline-based composite**								
Guan et al. 2021 (CTRCD, Sub-Clinical) (Mixture)	60.73	5.65	79	66.65	4.13	79	11.3%	-5.92 [-7.46; -4.38]
Cadeddu et al. 2017 (CTRCD, Clinical)(Mixture)	60.30	1.80	45	62.80	2.00	45	11.4%	-2.50 [-3.29; -1.71]
BenKridis et al. 2020 (CTRCD, Clinical)(Mixture)	59.71	4.24	50	62.48	3.04	50	11.3%	-2.77 [-4.22; -1.32]
**Total (95% CI)**			**174**			**174**	**34.0%**	**-3.67 [-8.34; 1.00]**
Heterogeneity: $Tau^2 = 3.0345$; $Chi^2 = 15.26$, df = 2 (P = 0.0005); $I^2 = 86.9\%$								
**CTRCD_Definition = LVEF-only**								
El□Sherbeny et al. 2019 (CTRCD, Clinical)	40.35	2.76	18	62.82	2.19	18	11.3%	-22.47 [-24.10; -20.84]
Charbonnel et al. 2017 (CTRCD, Sub-Clinical)	54.00	7.15	6	64.83	2.86	6	10.1%	-10.83 [-16.99; -4.67]
Hazim et al. 2023 (CTRCD, Subclinical) (with anthracycline) (Mixture)	57.50	6.00	20	63.00	4.00	20	11.0%	-5.50 [-8.66; -2.34]
Hazim et al. 2023 (CTRCD, Subclinical) (without anthracycline) (Mixture)	62.10	3.00	27	63.00	3.00	27	11.3%	-0.90 [-2.50; 0.70]
Fallah-Rad et al. 2011 (CTRCD, Clinical)	39.00	5.00	10	64.00	3.00	10	10.9%	-25.00 [-28.61; -21.39]
**Total (95% CI)**			**81**			**81**	**54.8%**	**-12.94 [-26.06; 0.18]**
Heterogeneity: $Tau^2 = 109.8536$; $Chi^2 = 409.84$, df = 4 (P < 0.0001); $I^2 = 99\%$								
**Total (95% CI)**			**306**			**306**	**100.0%**	**-8.69 [-15.60; -1.78]**
**Prediction interval**								**[-30.33; 12.95]**
Heterogeneity: $Tau^2 = 79.0164$; $Chi^2 = 638.11$, df = 8 (P < 0.0001); $I^2 = 98.7\%$								
Test for overall effect: $t_8 = -2.90$ (P = 0.0199)								
Test for subgroup differences: $Chi^2 = 4.15$, df = 2 (P = 0.1257)								

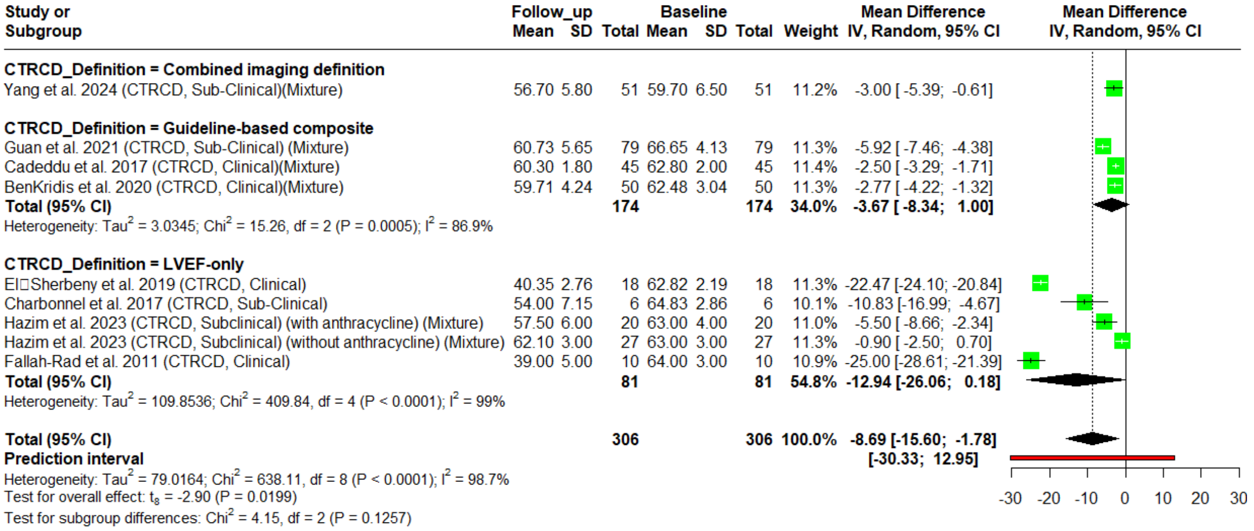

Change in LVEF at Last Visit in CTRCD patients

Study or Subgroup	Follow_up Mean	Follow_up SD	Follow_up Total	Baseline Mean	Baseline SD	Baseline Total	Weight	Mean Difference IV, Random, 95% CI
**CTRCD_Definition = Combined imaging definition**								
AboElMagdRasheed et al. 2022 (CTRCD, Clinical)(Mixture)	63.64	6.09	50	65.70	4.88	50	2.8%	-2.06 [-4.22; 0.10]
Yang et al. 2024 (CTRCD, Sub-Clinical)(Mixture)	56.90	5.90	51	59.70	6.50	51	2.8%	-2.80 [-5.21; -0.39]
Tahir et al. 2022 (CTRCD, Sub-Clinical) (Epirubicin□based (CMR))(Mixture)	60.00	2.00	38	60.00	5.00	39	2.9%	0.00 [-1.69; 1.69]
Tahir et al. 2022 (CTRCD, Sub-Clinical) (Radiotherapy (CMR)(Mixture)	62.00	5.00	27	62.00	5.00	27	2.7%	0.00 [-2.67; 2.67]
Cheng et al. 2023 (CTRCD, Clinical)(Mixture)	58.00	3.94	24	63.70	4.33	24	2.8%	-5.70 [-8.04; -3.36]
**Total (95% CI)**			**190**			**191**	**14.0%**	**-2.08 [-5.00; 0.84]**
Heterogeneity: $Tau^2 = 4.2124$; $Chi^2 = 17.45$, df = 4 (P = 0.0016); $I^2 = 77.1\%$								
**CTRCD_Definition = GLS-only**								
Mele et al. 2016 (CTRCD, Sub-Clinical)(Mixture)	63.00	3.00	15	63.00	3.00	15	2.8%	0.00 [-2.15; 2.15]
Chang et al. 2021 (CTRCD, Subclinical)	67.70	6.80	36	70.20	5.70	36	2.7%	-2.50 [-5.40; 0.40]
Stoodley et al., 2013 (CTRCD, Sub-Clinical)(Mixture)	58.00	3.00	50	58.00	3.00	78	2.9%	0.00 [-1.07; 1.07]
Liu et al.2022 (CTRCD, Sub-Clinical) (anthracycline without trastuzumab)(Mixture)	66.30	3.13	34	66.78	4.17	34	2.9%	-0.48 [-2.23; 1.27]
Liu et al.2022 (CTRCD, Sub-Clinical) (trastuzumab without anthracycline)(Mixture)	65.08	2.69	33	66.14	2.82	33	2.9%	-1.06 [-2.39; 0.27]
**Total (95% CI)**			**168**			**196**	**14.2%**	**-0.50 [-1.42; 0.42]**
Heterogeneity: $Tau^2 = < 0.0001$; $Chi^2 = 3.57$, df = 4 (P = 0.4680); $I^2 = 0\%$								
**CTRCD_Definition = Guideline-based composite**								
Moya et al. 2023 (Mild CTRCD, Clinical)	53.60	3.00	10	57.40	3.60	10	2.7%	-3.80 [-6.70; -0.90]
Moya et al. 2023 (Moderate CTRCD, Clinical)	48.90	10.10	9	55.00	4.50	9	1.9%	-6.10 [-13.32; 1.12]
Guan et al. 2021 (CTRCD, Sub-Clinical) (Mixture)	59.43	6.99	79	66.65	4.13	79	2.9%	-7.22 [-9.01; -5.43]
Gripp et al. 2018 (CTRCD, Sub-Clinical)	52.00	5.10	5	64.00	4.80	5	2.1%	-12.00 [-18.14; -5.86]
Cadeddu et al. 2017 (CTRCD, Clinical)(Mixture)	58.00	2.30	45	62.80	2.00	45	2.9%	-4.80 [-5.69; -3.91]
BenKridis et al. 2020 (CTRCD, Clinical)(Mixture)	59.00	3.30	50	62.48	3.04	50	2.9%	-3.48 [-4.72; -2.24]
Allam et al. 2023 (CTRCD, Sub-Clinical)	47.70	1.50	10	61.20	5.11	10	2.6%	-13.50 [-16.80; -10.20]
Kar et al. 2023 (CTRCD, Sub-Clinical)(Mixture)	58.30	6.50	32	58.50	6.00	32	2.7%	-0.20 [-3.26; 2.86]
Li et al. 2024 (CTRCD, Sub-Clinical)(Mixure)	57.08	5.83	80	61.30	4.73	80	2.9%	-4.22 [-5.87; -2.57]
**Total (95% CI)**			**320**			**320**	**23.6%**	**-5.83 [-8.99; -2.68]**
Heterogeneity: $Tau^2 = 13.4933$; $Chi^2 = 53.02$, df = 8 (P < 0.0001); $I^2 = 84.9\%$								
**CTRCD_Definition = Imaging plus biomarkers**								
Di Lisi 2023 (CTRCD, Sub-Clinical)	59.40	4.00	28	60.10	1.50	28	2.9%	-0.70 [-2.28; 0.88]
Di Lisi 2023 (CTRCD, Sub-Clinical)(Mixture)	59.50	2.00	61	60.00	3.21	61	2.9%	-0.50 [-1.45; 0.45]
Zhang et al. 2022 (CTRCD, Clinical)(Mixture)	56.51	8.55	145	60.92	5.78	207	2.9%	-4.41 [-6.01; -2.81]
Lenihan et al. 2025 (CTRCD, Clinical) Sub-CTX	57.00	6.00	24	62.00	5.00	24	2.7%	-5.00 [-8.12; -1.88]
Lenihan et al. 2025 (CTRCD, Clinical) CTX	48.00	7.00	9	57.00	5.00	9	2.2%	-9.00 [-14.62; -3.38]
**Total (95% CI)**			**267**			**329**	**13.6%**	**-3.25 [-7.19; 0.68]**
Heterogeneity: $Tau^2 = 7.1170$; $Chi^2 = 29.46$, df = 4 (P < 0.0001); $I^2 = 86.4\%$								
**CTRCD_Definition = LVEF-only**								
El□Sherbeny et al. 2019 (CTRCD, Clinical)	48.24	2.11	18	62.82	2.19	18	2.9%	-14.58 [-15.98; -13.18]
Charbonnel et al. 2017 (CTRCD, Sub-Clinical)	49.00	1.90	6	64.83	2.86	6	2.7%	-15.83 [-18.58; -13.08]
Caballero-Valderrama et al. 2024 (CTRCD, Clinical)	57.30	6.80	5	62.90	3.70	5	2.0%	-5.60 [-12.39; 1.19]
Hazim et al. 2023 (CTRCD, Subclinical) (with anthracycline) (Mixture)	60.00	2.00	20	63.00	4.00	20	2.8%	-3.00 [-4.96; -1.04]
Hazim et al. 2023 (CTRCD, Subclinical) (without anthracycline) (Mixture)	62.00	2.00	27	63.00	3.00	27	2.9%	-1.00 [-2.36; 0.36]
Bhagat et al. 2023 (CTRCD, Sub-Clinical) (Mixture)	59.66	5.45	29	62.66	3.88	31	2.8%	-3.00 [-5.41; -0.59]
Park et al., 2020 (CTRCD, Clinical) (Mixture)	46.90	4.80	13	65.00	6.40	72	2.7%	-18.10 [-21.10; -15.10]
Mornos et al. 2013 (CTRCD, Clinical)(Mixture)	58.00	5.90	74	61.00	5.60	74	2.9%	-3.00 [-4.85; -1.15]
Anqi et al. 2019 (CTRCD, Sub-Clinical)(Mixture)	59.94	5.07	40	69.76	6.13	40	2.8%	-9.82 [-12.29; -7.35]
Fallah-Rad et al. 2011 (CTRCD, Clinical)	49.00	4.00	10	64.00	3.00	10	2.7%	-15.00 [-18.10; -11.90]
Negishi et al. 2013 (CTRCD, Sub-Clinical)	55.00	5.30	24	64.00	4.60	24	2.7%	-9.00 [-11.81; -6.19]
**Total (95% CI)**			**266**			**327**	**29.9%**	**-8.91 [-13.08; -4.73]**
Heterogeneity: $Tau^2 = 36.9548$; $Chi^2 = 351.96$, df = 10 (P < 0.0001); $I^2 = 97.2\%$								
**CTRCD_Definition = Not reported**								
Huynh et al. 2025 (CTRCD, Sub-Clinical)(Mixture)	63.50	9.50	11	65.20	6.19	16	2.0%	-1.70 [-8.08; 4.68]
Kersten et al. 2023 (CTRCD, Clinical)(Mixture)	61.10	6.10	32	65.20	6.80	34	2.7%	-4.10 [-7.21; -0.99]
**Total (95% CI)**			**43**			**50**	**4.7%**	**-3.64 [-15.66; 8.38]**
Heterogeneity: $Tau^2 = 0$; $Chi^2 = 0.44$, df = 1 (P = 0.5076); $I^2 = 0\%$								
**Total (95% CI)**			**1254**			**1413**	**100.0%**	**-5.10 [-6.80; -3.41]**
**Prediction interval**								**[-15.13; 4.92]**

Heterogeneity: $Tau^2 = 23.7280$; $Chi^2 = 747.18$, df = 36 (P < 0.0001); $I^2 = 95.2\%$
Test for overall effect: $t_{36} = -6.12$ (P < 0.0001)
Test for subgroup differences: $Chi^2 = 41.64$, df = 5 (P < 0.0001)

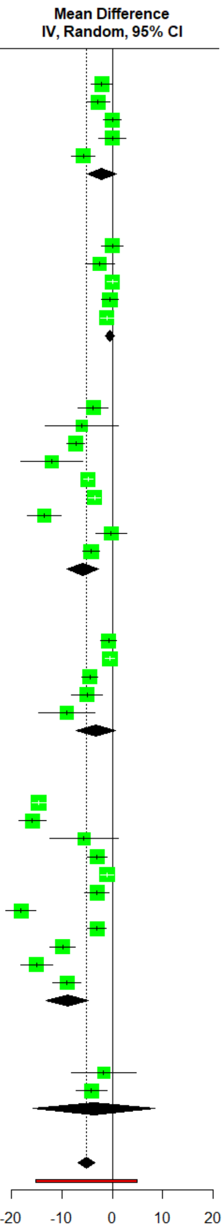

Change in GLS at Visit 1 in non-CTRCD patients

Study or Subgroup	Follow_up Mean	Follow_up SD	Follow_up Total	Baseline Mean	Baseline SD	Baseline Total	Weight	Mean Difference IV, Fixed, 95% CI
**CTRCD_Definition = GLS-only**								
Mele et al. 2016 (Non-CTRCD)	-22.20	2.10	15	-21.60	1.80	15	3.2%	-0.60 [-2.00; 0.80]
Chang et al. 2021 (Non-CTRCD)	-19.20	2.60	38	-19.30	2.20	38	5.4%	0.10 [-0.98; 1.18]
**Total (95% CI)**			**53**			**53**	**8.6%**	**-0.16 [-1.02; 0.69]**
Heterogeneity: $Tau^2 = 0$; $Chi^2 = 0.6$, df = 1 (P = 0.4382); $I^2 = 0\%$								
**CTRCD_Definition = Guideline-based composite**								
Moya et al. 2023 (Non-CTRCD)	-21.30	2.60	31	-20.40	2.50	31	3.9%	-0.90 [-2.17; 0.37]
Gripp et al. 2018 (Non-CTRCD)	-19.60	2.10	44	-20.50	2.00	44	8.5%	0.90 [0.04; 1.76]
Allam et al. 2023 (Non-CTRCD)	-17.00	3.10	70	-18.30	3.20	70	5.8%	1.30 [0.26; 2.34]
**Total (95% CI)**			**145**			**145**	**18.2%**	**0.64 [0.05; 1.23]**
Heterogeneity: $Tau^2 = 0.9353$; $Chi^2 = 7.54$, df = 2 (P = 0.0230); $I^2 = 73.5\%$								
**CTRCD_Definition = Imaging plus biomarkers**								
DiLisi et al. 2023 (Non-CTRCD)	-20.00	2.40	141	-20.40	2.00	141	23.6%	0.40 [-0.12; 0.92]
**CTRCD_Definition = LVEF-only**								
El□Sherbeny et al. 2019 (Non-CTRCD)	-20.12	1.35	43	-20.44	1.44	43	18.0%	0.32 [-0.27; 0.91]
Charbonnel et al. 2017 (Non-CTRCD)	-21.33	2.56	80	-21.33	2.56	80	10.0%	0.00 [-0.79; 0.79]
Caballero-Valderrama et al. 2024 (Non-CTRCD)	-21.08	2.40	28	-21.51	1.72	28	5.2%	0.43 [-0.66; 1.52]
Fallah-Rad et al. 2011 (Non-CTRCD)	-19.90	2.30	32	-20.20	2.40	32	4.7%	0.30 [-0.85; 1.45]
Negishi 2013 (Non-CTRCD)	-19.70	2.00	57	-20.00	2.00	57	11.6%	0.30 [-0.43; 1.03]
**Total (95% CI)**			**240**			**240**	**49.6%**	**0.26 [-0.09; 0.62]**
Heterogeneity: $Tau^2 = 0$; $Chi^2 = 0.56$, df = 4 (P = 0.9673); $I^2 = 0\%$								
**Total (95% CI)**			**579**			**579**	**100.0%**	**0.33 [0.08; 0.58]**
**Prediction interval**								**[0.04; 0.61]**

Heterogeneity: $Tau^2 < 0.0001$; $Chi^2 = 11.27$, df = 10 (P = 0.3369); $I^2 = 11.3\%$
Test for overall effect: Z = 2.56 (P = 0.0106)
Test for subgroup differences: $Chi^2 = 2.57$, df = 3 (P = 0.4635)

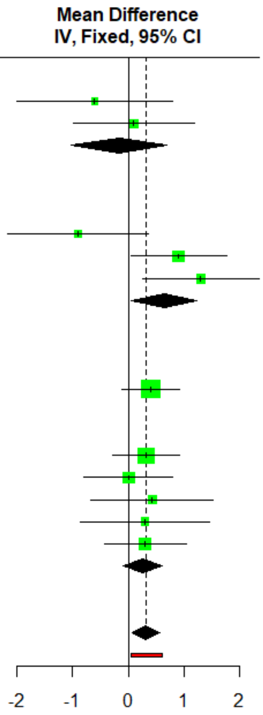

Change in GLS at Visit 2 in non-CTRCD patients

Study or Subgroup	Follow_up Mean	Follow_up SD	Follow_up Total	Baseline Mean	Baseline SD	Baseline Total	Weight	Mean Difference IV, Fixed, 95% CI
**CTRCD_Definition = GLS-only**								
Mele et al. 2016 (Non-CTRCD)	-21.00	1.60	15	-21.60	1.80	15	7.4%	0.60 [-0.62; 1.82]
Chang et al. 2021 (Non-CTRCD)	-19.60	2.70	38	-19.30	2.20	38	8.9%	-0.30 [-1.41; 0.81]
**Total (95% CI)**			**53**			**53**	**16.3%**	**0.11 [-0.71; 0.93]**
Heterogeneity: $Tau^2 = 0.0521$; $Chi^2 = 1.15$, df = 1 (P = 0.2841); $I^2 = 12.9\%$								
**CTRCD_Definition = Guideline-based composite**								
Moya et al. 2023 (Non-CTRCD)	-20.30	2.00	31	-20.40	2.50	31	8.6%	0.10 [-1.03; 1.23]
**CTRCD_Definition = LVEF-only**								
El□Sherbeny et al. 2019 (Non-CTRCD)	-19.63	1.25	43	-20.44	1.44	43	33.6%	0.81 [0.24; 1.38]
Charbonnel et al. 2017 (Non-CTRCD)	-21.20	2.11	80	-21.33	2.56	80	20.7%	0.13 [-0.60; 0.86]
Caballero-Valderrama et al. 2024 (Non-CTRCD)	-20.63	1.63	28	-21.51	1.72	28	14.2%	0.88 [0.00; 1.76]
Fallah-Rad et al. 2011 (Non-CTRCD)	-19.40	2.80	32	-20.20	2.40	32	6.7%	0.80 [-0.48; 2.08]
**Total (95% CI)**			**183**			**183**	**75.1%**	**0.64 [0.25; 1.02]**
Heterogeneity: $Tau^2 = 0.0089$; $Chi^2 = 2.58$, df = 3 (P = 0.4611); $I^2 = 0\%$								
**Total (95% CI)**			**267**			**267**	**100.0%**	**0.50 [0.17; 0.83]**
**Prediction interval**								**[-0.06; 1.04]**

Heterogeneity: $Tau^2 = 0.0185$; $Chi^2 = 5.58$, df = 6 (P = 0.4721); $I^2 = 0.0\%$
Test for overall effect: Z = 2.99 (P = 0.0028)
Test for subgroup differences: $Chi^2 = 1.85$, df = 2 (P = 0.3964)

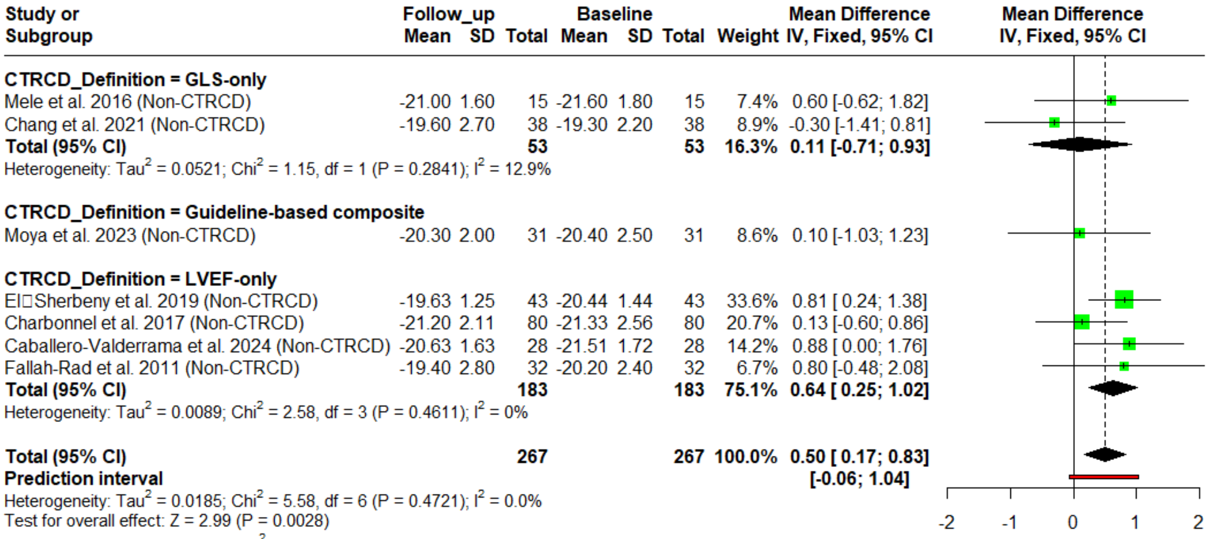

Change in GLS at Visit 3 in non-CTRCD patients

Study or Subgroup	Follow_up Mean	SD	Total	Baseline Mean	SD	Total	Weight	Mean Difference IV, Random, 95% CI
**CTRCD_Definition = LVEF-only**								
El□Sherbeny et al. 2019 (Non-CTRCD)	-19.48	1.00	43	-20.44	1.44	43	40.1%	0.96 [0.44; 1.48]
Charbonnel et al. 2017 (Non-CTRCD)	-21.56	2.34	80	-21.33	2.56	80	33.3%	-0.23 [-0.99; 0.53]
Fallah-Rad et al. 2011 (Non-CTRCD)	-20.10	1.70	32	-20.20	2.40	32	26.5%	0.10 [-0.92; 1.12]
**Total (95% CI)**			**155**			**155**	**100.0%**	**0.34 [-1.27; 1.94]**
Heterogeneity: Tau^2 = 0.3162; Chi^2 = 7.1, df = 2 (P = 0.0288); I^2 = 71.8%								
**Total (95% CI)**			**155**			**155**	**100.0%**	**0.34 [-1.27; 1.94]**
**Prediction interval**								**[-2.62; 3.29]**

Heterogeneity: Tau^2 = 0.3162; Chi^2 = 7.10, df = 2 (P = 0.0288); I^2 = 71.8%
Test for overall effect: t_2 = 0.90 (P = 0.4635)
Test for subgroup differences: Chi^2 = 0.00, df = 0 (P = NA)

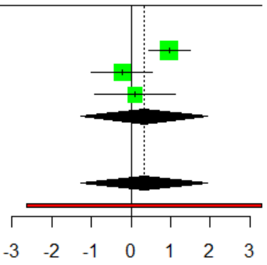

Change in GLS at Last Visit in non-CTRCD patients

Study or Subgroup	Follow_up Mean	SD	Total	Baseline Mean	SD	Total	Weight	Mean Difference IV, Fixed, 95% CI
**CTRCD_Definition = GLS-only**								
Mele et al. 2016 (Non-CTRCD)	-21.30	1.50	15	-21.60	1.80	15	5.0%	0.30 [-0.89; 1.49]
Chang et al. 2021 (Non-CTRCD)	-18.50	2.50	38	-19.30	2.20	38	6.2%	0.80 [-0.26; 1.86]
**Total (95% CI)**			**53**			**53**	**11.2%**	**0.58 [-0.21; 1.37]**
Heterogeneity: Tau^2 = 0; Chi^2 = 0.38, df = 1 (P = 0.5376); I^2 = 0%								
**CTRCD_Definition = Guideline-based composite**								
Moya et al. 2023 (Non-CTRCD)	-20.40	2.90	31	-20.40	2.50	31	3.8%	0.00 [-1.35; 1.35]
Gripp et al. 2018 (Non-CTRCD)	-19.40	2.00	44	-20.50	2.00	44	10.0%	1.10 [0.26; 1.94]
Allam et al. 2023 (Non-CTRCD)	-17.20	2.80	70	-18.30	3.20	70	7.0%	1.10 [0.10; 2.10]
**Total (95% CI)**			**145**			**145**	**20.9%**	**0.90 [0.32; 1.48]**
Heterogeneity: Tau^2 = < 0.0001; Chi^2 = 2.09, df = 2 (P = 0.3521); I^2 = 4.2%								
**CTRCD_Definition = Imaging plus biomarkers**								
DiLisi et al. 2023 (Non-CTRCD)	-19.60	2.30	141	-20.40	2.00	141	27.6%	0.80 [0.30; 1.30]
**CTRCD_Definition = LVEF-only**								
El□Sherbeny et al. 2019 (Non-CTRCD)	-17.77	8.42	43	-20.44	1.44	43	1.1%	2.67 [0.12; 5.22]
Charbonnel et al. 2017 (Non-CTRCD)	-20.53	1.58	80	-21.33	2.56	80	16.1%	0.80 [0.14; 1.46]
Caballero-Valderrama et al. 2024 (Non-CTRCD)	-20.71	2.27	28	-21.51	1.72	28	6.3%	0.80 [-0.25; 1.85]
Fallah-Rad et al. 2011 (Non-CTRCD)	-19.80	1.90	32	-20.20	2.40	32	6.2%	0.40 [-0.66; 1.46]
Negishi 2013 (Non-CTRCD)	-19.50	2.40	57	-20.00	2.00	57	10.6%	0.50 [-0.31; 1.31]
**Total (95% CI)**			**240**			**240**	**40.3%**	**0.71 [0.29; 1.13]**
Heterogeneity: Tau^2 = < 0.0001; Chi^2 = 2.95, df = 4 (P = 0.5664); I^2 = 0%								
**Total (95% CI)**			**579**			**579**	**100.0%**	**0.76 [0.49; 1.02]**
**Prediction interval**								**[0.46; 1.06]**

Heterogeneity: Tau^2 < 0.0001; Chi^2 = 5.92, df = 10 (P = 0.8220); I^2 = 0.0%
Test for overall effect: Z = 5.63 (P < 0.0001)
Test for subgroup differences: Chi^2 = 0.50, df = 3 (P = 0.9183)

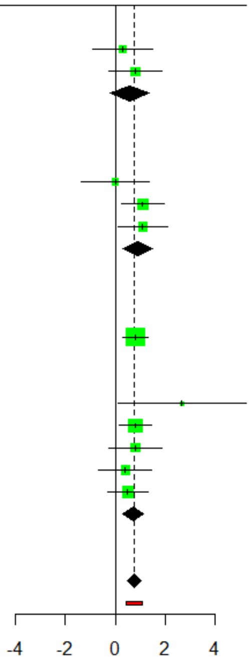

Change in LVEF at Visit 1 in non-CTRCD patients

Study or Subgroup	Follow_up Mean	SD	Total	Baseline Mean	SD	Total	Weight	Mean Difference IV, Fixed, 95% CI
**CTRCD_Definition = GLS-only**								
Mele et al. 2016 (Non-CTRCD)	63.00	3.00	15	63.00	3.00	15	3.1%	0.00 [-2.15; 2.15]
Chang et al. 2021 (Non-CTRCD)	71.00	7.30	38	67.80	7.90	38	1.2%	3.20 [-0.22; 6.62]
**Total (95% CI)**			**53**			**53**	**4.3%**	**0.90 [-0.91; 2.72]**
Heterogeneity: $Tau^2 = 2.9976$; $Chi^2 = 2.41$, df = 1 (P = 0.1204); $I^2 = 58.5\%$								
**CTRCD_Definition = Guideline-based composite**								
Moya et al. 2023 (Non-CTRCD)	57.50	5.40	31	57.60	4.30	31	2.4%	-0.10 [-2.53; 2.33]
Gripp et al. 2018 (Non-CTRCD)	67.20	6.40	44	68.30	7.70	44	1.6%	-1.10 [-4.06; 1.86]
Allam et al. 2023 (Non-CTRCD)	58.70	6.30	70	61.70	4.85	70	4.1%	-3.00 [-4.86; -1.14]
**Total (95% CI)**			**145**			**145**	**8.1%**	**-1.76 [-3.08; -0.44]**
Heterogeneity: $Tau^2 = 1.2916$; $Chi^2 = 3.69$, df = 2 (P = 0.1583); $I^2 = 45.7\%$								
**CTRCD_Definition = Imaging plus biomarkers**								
DiLisi et al. 2023 (Non-CTRCD)	60.00	2.30	141	60.00	1.80	141	60.7%	0.00 [-0.48; 0.48]
**CTRCD_Definition = LVEF-only**								
El□Sherbeny et al. 2019 (Non-CTRCD)	61.02	2.89	43	62.19	2.95	43	9.3%	-1.17 [-2.40; 0.06]
Charbonnel et al. 2017 (Non-CTRCD)	66.00	4.52	80	65.66	5.28	80	6.1%	0.34 [-1.18; 1.86]
Caballero-Valderrama et al. 2024 (Non-CTRCD)	60.80	4.00	28	61.30	5.10	28	2.4%	-0.50 [-2.90; 1.90]
Fallah-Rad et al. 2011 (Non-CTRCD)	60.00	8.00	32	62.00	5.00	32	1.3%	-2.00 [-5.27; 1.27]
Negishi 2013 (Non-CTRCD)	60.00	3.70	57	62.00	3.60	57	7.9%	-2.00 [-3.34; -0.66]
**Total (95% CI)**			**240**			**240**	**27.0%**	**-1.05 [-1.77; -0.33]**
Heterogeneity: $Tau^2 = 0.4220$; $Chi^2 = 5.69$, df = 4 (P = 0.2233); $I^2 = 29.7\%$								
**Total (95% CI)**			**579**			**579**	**100.0%**	**-0.39 [-0.76; -0.01]**
**Prediction interval**								**[-2.88; 1.48]**

Heterogeneity: $Tau^2 = 0.7993$; $Chi^2 = 23.59$, df = 10 (P = 0.0088); $I^2 = 57.6\%$
Test for overall effect: Z = -2.02 (P = 0.0435)
Test for subgroup differences: $Chi^2 = 11.80$, df = 3 (P = 0.0081)

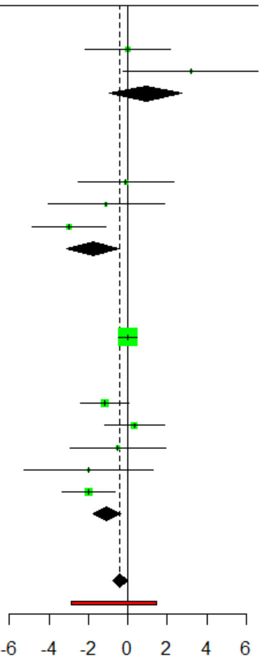

Change in LVEF at Visit 2 in non-CTRCD patients

Study or Subgroup	Follow_up Mean	SD	Total	Baseline Mean	SD	Total	Weight	Mean Difference IV, Fixed, 95% CI
**CTRCD_Definition = GLS-only**								
Mele et al. 2016 (Non-CTRCD)	64.00	3.00	15	63.00	3.00	15	11.3%	1.00 [-1.15; 3.15]
Chang et al. 2021 (Non-CTRCD)	70.50	5.90	38	67.80	7.90	38	5.3%	2.70 [-0.43; 5.83]
**Total (95% CI)**			**53**			**53**	**16.7%**	**1.54 [-0.23; 3.31]**
Heterogeneity: $Tau^2 = 0$; $Chi^2 = 0.77$, df = 1 (P = 0.3805); $I^2 = 0\%$								
**CTRCD_Definition = Guideline-based composite**								
Moya et al. 2023 (Non-CTRCD)	56.00	4.60	31	57.60	4.30	31	10.6%	-1.60 [-3.82; 0.62]
**CTRCD_Definition = LVEF-only**								
El□Sherbeny et al. 2019 (Non-CTRCD)	61.67	3.11	43	62.19	2.95	43	31.9%	-0.52 [-1.80; 0.76]
Charbonnel et al. 2017 (Non-CTRCD)	65.00	4.52	80	65.66	5.28	80	22.6%	-0.66 [-2.18; 0.86]
Caballero-Valderrama et al. 2024 (Non-CTRCD)	61.50	4.90	28	61.30	5.10	28	7.6%	0.20 [-2.42; 2.82]
Fallah-Rad et al. 2011 (Non-CTRCD)	64.00	4.00	32	62.00	5.00	32	10.6%	2.00 [-0.22; 4.22]
**Total (95% CI)**			**183**			**183**	**72.7%**	**-0.12 [-0.97; 0.73]**
Heterogeneity: $Tau^2 = 0.3390$; $Chi^2 = 4.42$, df = 3 (P = 0.2193); $I^2 = 32.2\%$								
**Total (95% CI)**			**267**			**267**	**100.0%**	**0.00 [-0.72; 0.72]**
**Prediction interval**								**[-2.18; 2.51]**

Heterogeneity: $Tau^2 = 0.6655$; $Chi^2 = 10.18$, df = 6 (P = 0.1172); $I^2 = 41.1\%$
Test for overall effect: Z = 0.00 (P = 0.9998)
Test for subgroup differences: $Chi^2 = 4.99$, df = 2 (P = 0.0824)

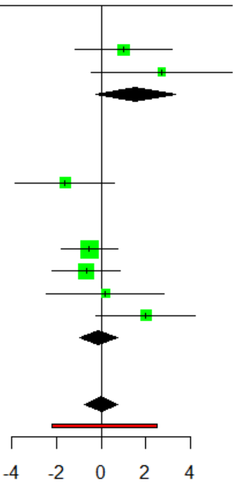

Change in LVEF at Visit 3 in non-CTRCD patients

Study or Subgroup	Follow_up Mean	SD	Total	Baseline Mean	SD	Total	Weight	Mean Difference IV, Random, 95% CI
**CTRCD_Definition = LVEF-only**								
El□Sherbeny et al. 2019 (Non-CTRCD)	60.95	2.52	43	62.19	2.95	43	35.8%	-1.24 [-2.40; -0.08]
Charbonnel et al. 2017 (Non-CTRCD)	64.50	4.52	80	65.66	5.28	80	34.1%	-1.16 [-2.68; 0.36]
Fallah-Rad et al. 2011 (Non-CTRCD)	65.00	4.00	32	62.00	5.00	32	30.1%	3.00 [0.78; 5.22]
**Total (95% CI)**			**155**			**155**	**100.0%**	**0.06 [-5.80; 5.93]**
Heterogeneity: $Tau^2 = 4.5806$; $Chi^2 = 11.8$, df = 2 (P = 0.0027); $I^2 = 83.1\%$								
**Total (95% CI)**			**155**			**155**	**100.0%**	**0.06 [-5.80; 5.93]**
**Prediction interval**								**[-10.77; 10.90]**

Heterogeneity: $Tau^2 = 4.5806$; $Chi^2 = 11.80$, df = 2 (P = 0.0027); $I^2 = 83.1\%$
Test for overall effect: $t_2 = 0.05$ (P = 0.9665)
Test for subgroup differences: $Chi^2 = 0.00$, df = 0 (P = NA)

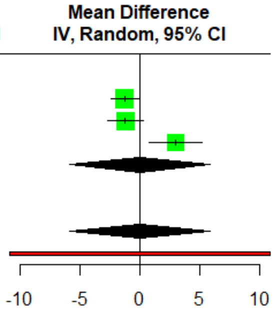

Change in LVEF at Last Visit in non-CTRCD patients

Study or Subgroup	Follow_up Mean	SD	Total	Baseline Mean	SD	Total	Weight	Mean Difference IV, Fixed, 95% CI
**CTRCD_Definition = GLS-only**								
Mele et al. 2016 (Non-CTRCD)	63.00	2.00	15	63.00	3.00	15	5.1%	0.00 [-1.82; 1.82]
Chang et al. 2021 (Non-CTRCD)	69.70	6.90	38	67.80	7.90	38	1.5%	1.90 [-1.43; 5.23]
**Total (95% CI)**			**53**			**53**	**6.6%**	**0.44 [-1.16; 2.04]**
Heterogeneity: $Tau^2 = 0$; $Chi^2 = 0.96$, df = 1 (P = 0.3273); $I^2 = 0\%$								
**CTRCD_Definition = Guideline-based composite**								
Moya et al. 2023 (Non-CTRCD)	55.90	4.70	31	57.60	4.30	31	3.4%	-1.70 [-3.94; 0.54]
Gripp et al. 2018 (Non-CTRCD)	67.40	6.60	44	68.30	7.70	44	1.9%	-0.90 [-3.90; 2.10]
Allam et al. 2023 (Non-CTRCD)	58.70	6.30	70	61.70	4.85	70	4.9%	-3.00 [-4.86; -1.14]
**Total (95% CI)**			**145**			**145**	**10.2%**	**-2.18 [-3.47; -0.88]**
Heterogeneity: $Tau^2 = 0$; $Chi^2 = 1.62$, df = 2 (P = 0.4445); $I^2 = 0\%$								
**CTRCD_Definition = Imaging plus biomarkers**								
DiLisi et al. 2023 (Non-CTRCD)	59.00	3.20	141	60.00	1.80	141	46.3%	-1.00 [-1.61; -0.39]
**CTRCD_Definition = LVEF-only**								
El□Sherbeny et al. 2019 (Non-CTRCD)	60.84	2.22	43	62.19	2.95	43	14.0%	-1.35 [-2.45; -0.25]
Charbonnel et al. 2017 (Non-CTRCD)	63.33	3.77	80	65.66	5.28	80	8.4%	-2.33 [-3.75; -0.91]
Caballero-Valderrama et al. 2024 (Non-CTRCD)	59.70	3.70	28	61.30	5.10	28	3.1%	-1.60 [-3.93; 0.73]
Fallah-Rad et al. 2011 (Non-CTRCD)	61.00	9.00	32	62.00	5.00	32	1.3%	-1.00 [-4.57; 2.57]
Negishi 2013 (Non-CTRCD)	60.00	3.50	57	62.00	3.60	57	10.0%	-2.00 [-3.30; -0.70]
**Total (95% CI)**			**240**			**240**	**36.9%**	**-1.76 [-2.44; -1.08]**
Heterogeneity: $Tau^2 = 0$; $Chi^2 = 1.47$, df = 4 (P = 0.8319); $I^2 = 0\%$								
**Total (95% CI)**			**579**			**579**	**100.0%**	**-1.30 [-1.72; -0.89]**
**Prediction interval**								**[-2.34; -0.40]**

Heterogeneity: $Tau^2 = 0.1204$; $Chi^2 = 13.04$, df = 10 (P = 0.2214); $I^2 = 23.3\%$
Test for overall effect: Z = -6.20 (P < 0.0001)
Test for subgroup differences: $Chi^2 = 8.99$, df = 3 (P = 0.0294)

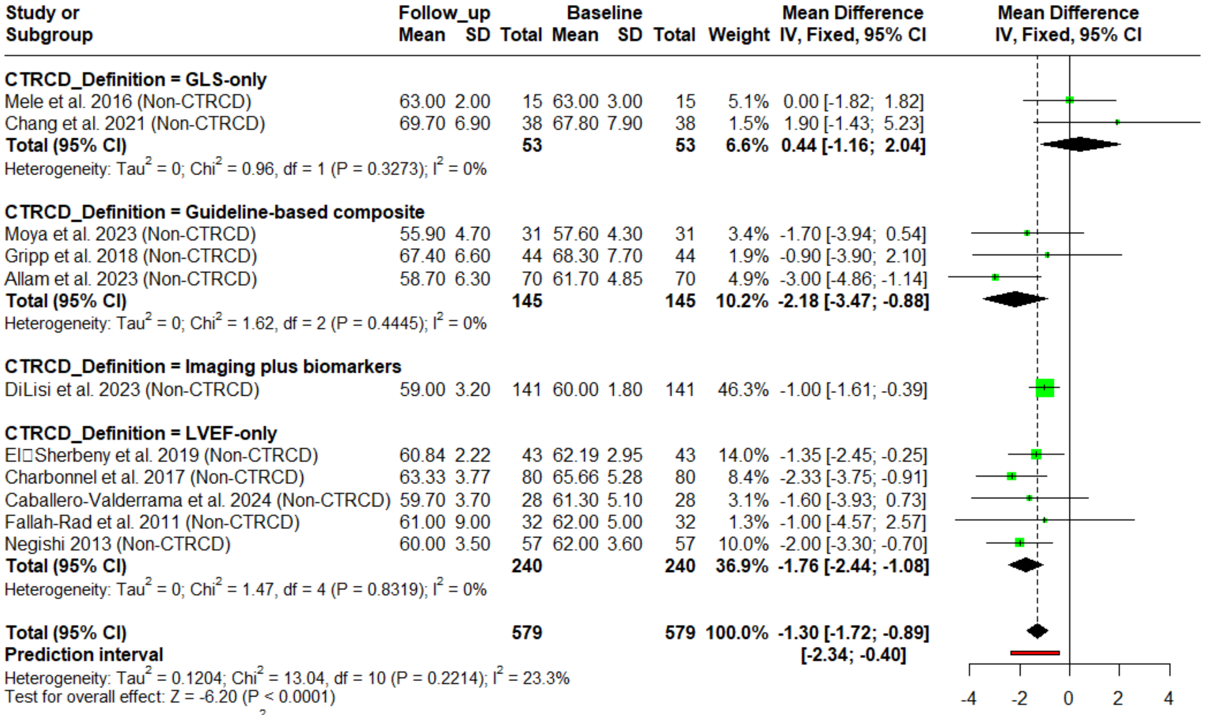

CMR-derived GLS Change at Visit 1 in CTRCD patients

Study or Subgroup	Follow_up Mean	Follow_up SD	Follow_up Total	Baseline Mean	Baseline SD	Baseline Total	Weight	Mean Difference IV, Random, 95% CI
**CTRCD_Definition = Combined imaging definition**								
Tahir et al. 2022 (CTRCD, Sub-Clinical) (Epirubicin□based (CMR))(Mixture)	-17.00	2.00	39	-18.00	2.00	39	22.2%	1.00 [0.11; 1.89]
Tahir et al. 2022 (CTRCD, Sub-Clinical) (Radiotherapy (CMR)(Mixture)	-18.00	2.00	27	-18.00	2.00	27	20.2%	0.00 [-1.07; 1.07]
**Total (95% CI)**			**66**			**66**	**42.4%**	**0.55 [-5.78; 6.87]**
Heterogeneity: Tau^2 = 0.2493; Chi^2 = 1.99, df = 1 (P = 0.1579); I^2 = 49.9%								
**CTRCD_Definition = Guideline-based composite**								
Kar etal. 2023 (CTRCD, Subclinical) (CMR)	-16.10	2.40	32	-18.80	1.50	32	21.2%	2.70 [1.72; 3.68]
**CTRCD_Definition = Not reported**								
Huynh etal. 2025 (CTRCD, Subclinical) (CMR)	-18.40	1.44	11	-19.16	2.71	16	15.1%	0.76 [-0.82; 2.34]
Kersten et al. 2023 (CTRCD, Clinical)(Mixture)	-13.60	1.90	33	-14.90	2.10	34	21.4%	1.30 [0.34; 2.26]
**Total (95% CI)**			**44**			**50**	**36.5%**	**1.15 [-1.89; 4.20]**
Heterogeneity: Tau^2 = 0; Chi^2 = 0.33, df = 1 (P = 0.5663); I^2 = 0%								
**Total (95% CI)**			**142**			**148**	**100.0%**	**1.19 [-0.06; 2.43]**
**Prediction interval**								**[-1.52; 3.89]**

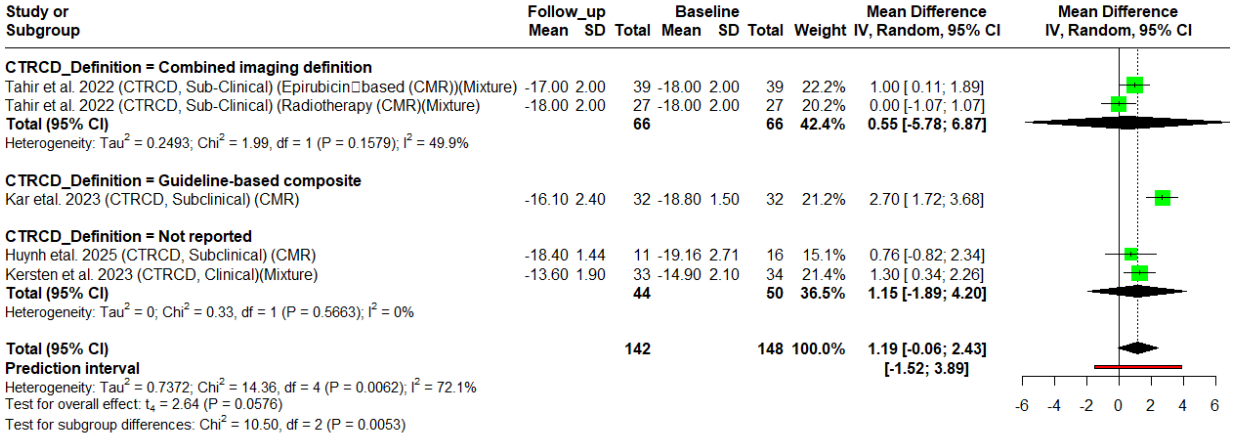

Heterogeneity: Tau^2 = 0.7372; Chi^2 = 14.36, df = 4 (P = 0.0062); I^2 = 72.1%
Test for overall effect: t_4 = 2.64 (P = 0.0576)
Test for subgroup differences: Chi^2 = 10.50, df = 2 (P = 0.0053)

CMR-derived GLS Change at Last Visit in CTRCD patients

Study or Subgroup	Follow_up Mean	Follow_up SD	Follow_up Total	Baseline Mean	Baseline SD	Baseline Total	Weight	Mean Difference IV, Random, 95% CI
**CTRCD_Definition = Combined imaging definition**								
Tahir et al. 2022 (CTRCD, Sub-Clinical) (Epirubicin□based (CMR))(Mixture)	-17.00	2.00	38	-18.00	2.00	39	14.0%	1.00 [0.11; 1.89]
Tahir et al. 2022 (CTRCD, Sub-Clinical) (Radiotherapy (CMR)(Mixture)	-18.00	1.00	27	-18.00	2.00	27	14.5%	0.00 [-0.84; 0.84]
Cheng et al. 2023 (CTRCD, Clinical)(Mixture)	-15.00	2.00	24	-16.00	1.60	24	12.9%	1.00 [-0.02; 2.02]
**Total (95% CI)**			**89**			**90**	**41.5%**	**0.63 [-0.83; 2.10]**
Heterogeneity: Tau^2 = 0.1481; Chi^2 = 3.3, df = 2 (P = 0.1923); I^2 = 39.4%								
**CTRCD_Definition = Guideline-based composite**								
Kar etal. 2023 (CTRCD, Subclinical) (CMR)	-16.00	2.50	32	-18.80	1.50	32	13.1%	2.80 [1.79; 3.81]
**CTRCD_Definition = Imaging plus biomarkers**								
Lenihan et al. 2025 (CTRCD, Clinical) Sub-CTX	-18.00	1.00	24	-20.00	2.00	24	14.0%	2.00 [1.11; 2.89]
Lenihan et al. 2025 (CTRCD, Clinical) CTX	-17.00	1.00	9	-19.00	2.00	9	9.6%	2.00 [0.54; 3.46]
**Total (95% CI)**			**33**			**33**	**23.7%**	**2.00 [2.00; 2.00]**
Heterogeneity: Tau^2 = 0; Chi^2 = 0, df = 1 (P = 1.0000); I^2 = 0%								
**CTRCD_Definition = Not reported**								
Huynh etal. 2025 (CTRCD, Subclinical) (CMR)	-17.53	1.00	11	-19.16	2.71	16	9.7%	1.63 [0.18; 3.08]
Kersten et al. 2023 (CTRCD, Clinical)(Mixture)	-13.80	2.50	32	-14.90	2.10	34	12.2%	1.10 [-0.02; 2.22]
**Total (95% CI)**			**43**			**50**	**21.8%**	**1.30 [-1.96; 4.55]**
Heterogeneity: Tau^2 = 0; Chi^2 = 0.32, df = 1 (P = 0.5710); I^2 = 0%								
**Total (95% CI)**			**197**			**205**	**100.0%**	**1.40 [0.66; 2.14]**
**Prediction interval**								**[-0.51; 3.31]**

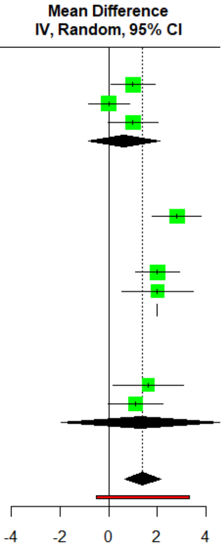

Heterogeneity: Tau^2 = 0.5475; Chi^2 = 21.90, df = 7 (P = 0.0026); I^2 = 68.0%
Test for overall effect: t_7 = 4.48 (P = 0.0029)
Test for subgroup differences: Chi^2 = 12.30, df = 3 (P = 0.0064)

CMR-derived LVEF Change at Visit 1 in CTRCD patients

Study or Subgroup	Follow_up Mean	Follow_up SD	Follow_up Total	Baseline Mean	Baseline SD	Baseline Total	Weight	Mean Difference IV, Fixed, 95% CI
**CTRCD_Definition = Combined imaging definition**								
Tahir et al. 2022 (CTRCD, Sub-Clinical) (Epirubicin□based (CMR))(Mixture)	60.00	6.00	39	60.00	5.00	39	31.9%	0.00 [-2.45; 2.45]
Tahir et al. 2022 (CTRCD, Sub-Clinical) (Radiotherapy (CMR)(Mixture)	64.00	6.00	27	62.00	5.00	27	22.1%	2.00 [-0.95; 4.95]
**Total (95% CI)**			**66**			**66**	**54.0%**	**0.82 [-1.07; 2.70]**
Heterogeneity: $Tau^2 = 0.0883$; $Chi^2 = 1.05$, df = 1 (P = 0.3064); $I^2 = 4.4\%$								
**CTRCD_Definition = Guideline-based composite**								
Kar etal. 2023 (CTRCD, Subclinical) (CMR)	57.30	6.60	32	58.00	5.60	32	21.3%	-0.70 [-3.70; 2.30]
**CTRCD_Definition = Not reported**								
Huynh etal. 2025 (CTRCD, Subclinical) (CMR)	70.30	7.63	11	72.50	6.36	16	6.4%	-2.20 [-7.68; 3.28]
Kersten et al. 2023 (CTRCD, Clinical)(Mixture)	61.20	6.70	33	65.20	6.80	34	18.3%	-4.00 [-7.23; -0.77]
**Total (95% CI)**			**44**			**50**	**24.7%**	**-3.54 [-6.32; -0.75]**
Heterogeneity: $Tau^2 = 0$; $Chi^2 = 0.31$, df = 1 (P = 0.5793); $I^2 = 0\%$								
**Total (95% CI)**			**142**			**148**	**100.0%**	**-0.58 [-1.97; 0.80]**
**Prediction interval**								**[-6.14; 4.65]**

Heterogeneity: $Tau^2 = 2.6824$; $Chi^2 = 7.80$, df = 4 (P = 0.0991); $I^2 = 48.7\%$
Test for overall effect: Z = -0.82 (P = 0.4104)
Test for subgroup differences: $Chi^2 = 6.45$, df = 2 (P = 0.0398)

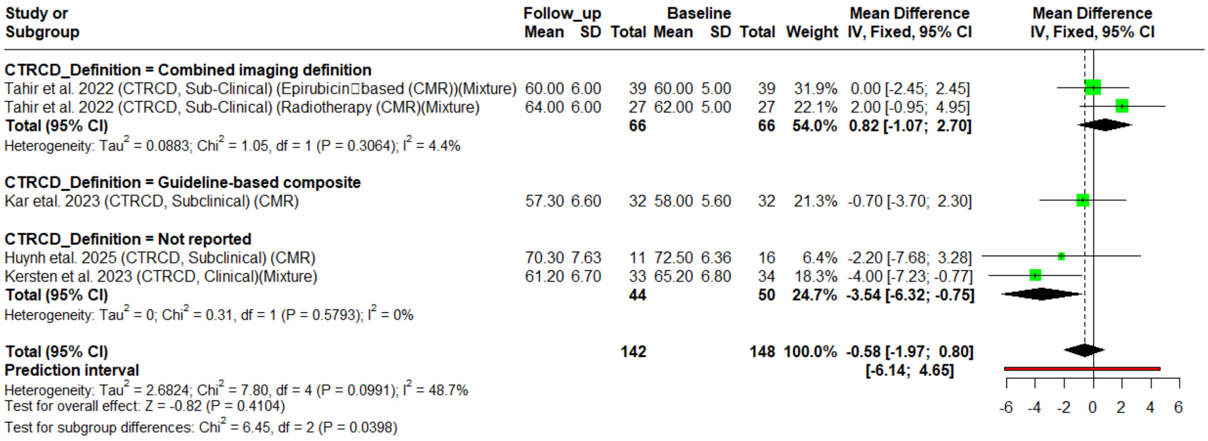

CMR-derived LVEF Change at Last Visit in CTRCD patients

Study or Subgroup	Follow_up Mean	Follow_up SD	Follow_up Total	Baseline Mean	Baseline SD	Baseline Total	Weight	Mean Difference IV, Random, 95% CI
**CTRCD_Definition = Combined imaging definition**								
Tahir et al. 2022 (CTRCD, Sub-Clinical) (Epirubicin□based (CMR))(Mixture)	60.00	2.00	38	60.00	5.00	39	15.3%	0.00 [-1.69; 1.69]
Tahir et al. 2022 (CTRCD, Sub-Clinical) (Radiotherapy (CMR)(Mixture)	62.00	5.00	27	62.00	5.00	27	13.6%	0.00 [-2.67; 2.67]
Cheng et al. 2023 (CTRCD, Clinical)(Mixture)	58.00	3.94	24	63.70	4.33	24	14.2%	-5.70 [-8.04; -3.36]
**Total (95% CI)**			**89**			**90**	**43.2%**	**-1.88 [-10.04; 6.27]**
Heterogeneity: $Tau^2 = 9.4208$; $Chi^2 = 16.58$, df = 2 (P = 0.0003); $I^2 = 87.9\%$								
**CTRCD_Definition = Guideline-based composite**								
Kar etal. 2023 (CTRCD, Subclinical) (CMR)	57.80	6.10	32	58.00	5.60	32	13.2%	-0.20 [-3.07; 2.67]
**CTRCD_Definition = Imaging plus biomarkers**								
Lenihan et al. 2025 (CTRCD, Clinical) Sub-CTX	57.00	6.00	24	62.00	5.00	24	12.7%	-5.00 [-8.12; -1.88]
Lenihan et al. 2025 (CTRCD, Clinical) CTX	48.00	7.00	9	57.00	5.00	9	8.3%	-9.00 [-14.62; -3.38]
**Total (95% CI)**			**33**			**33**	**21.0%**	**-6.29 [-30.05; 17.47]**
Heterogeneity: $Tau^2 = 2.6181$; $Chi^2 = 1.49$, df = 1 (P = 0.2228); $I^2 = 32.7\%$								
**CTRCD_Definition = Not reported**								
Huynh etal. 2025 (CTRCD, Subclinical) (CMR)	65.33	5.93	11	72.50	6.36	16	9.8%	-7.17 [-11.86; -2.48]
Kersten et al. 2023 (CTRCD, Clinical)(Mixture)	61.10	6.10	32	65.20	6.80	34	12.8%	-4.10 [-7.21; -0.99]
**Total (95% CI)**			**43**			**50**	**22.6%**	**-5.11 [-23.46; 13.23]**
Heterogeneity: $Tau^2 = 0.5886$; $Chi^2 = 1.14$, df = 1 (P = 0.2851); $I^2 = 12.5\%$								
**Total (95% CI)**			**197**			**205**	**100.0%**	**-3.45 [-6.25; -0.64]**
**Prediction interval**								**[-10.71; 3.81]**

Heterogeneity: $Tau^2 = 8.0784$; $Chi^2 = 33.79$, df = 7 (P < 0.0001); $I^2 = 79.3\%$
Test for overall effect: $t_7 = -2.91$ (P = 0.0227)
Test for subgroup differences: $Chi^2 = 9.17$, df = 3 (P = 0.0271)

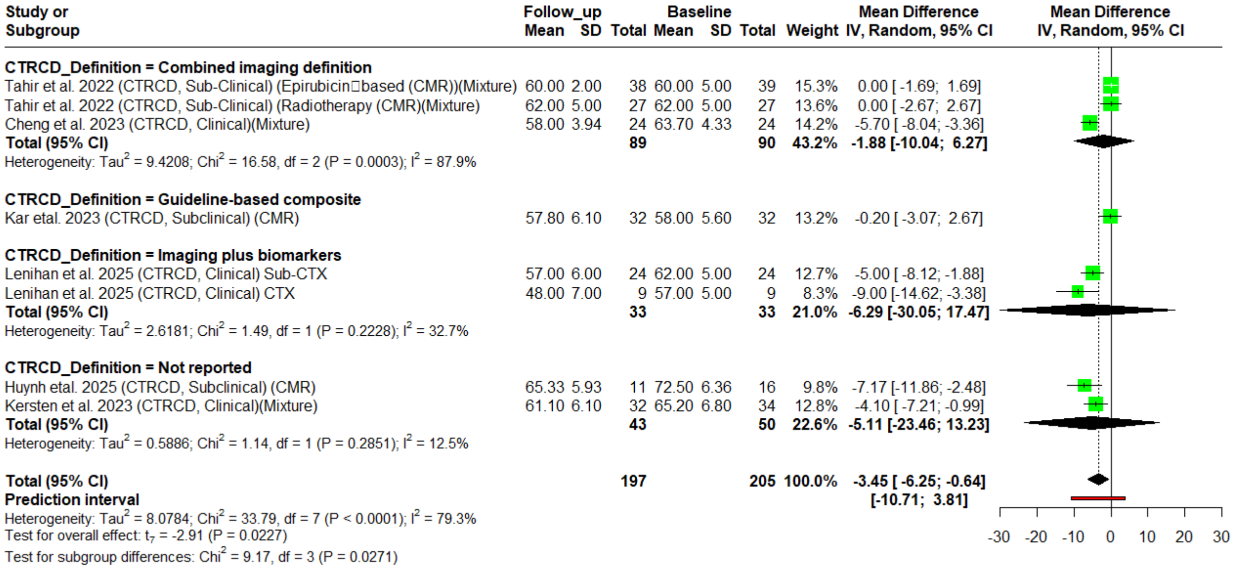

Supplement: Supplementary file 1 [file jcm-15-04520-s001.zip › jcm-4304318-supplementary.pdf]
